# Integrated single‐cell and spatial transcriptomic profiling reveals higher intratumour heterogeneity and epithelial–fibroblast interactions in recurrent bladder cancer

**DOI:** 10.1002/ctm2.1338

**Published:** 2023-07-24

**Authors:** Zhen‐Duo Shi, Zhuo Sun, Zuo‐Bin Zhu, Xing Liu, Jun‐Zhi Chen, Lin Hao, Jie‐Fei Zhu, Kun Pang, Di Wu, Yang Dong, Yu‐Fei Liu, Wei‐Hua Chen, Qing Liang, Shi‐Chao Zhuo, Cong‐Hui Han

**Affiliations:** ^1^ Department of Urology Xuzhou Clinical School of Xuzhou Medical University Xuzhou Central Hospital Xuzhou Jiangsu China; ^2^ School of Life Sciences Jiangsu Normal University Jiangsu China; ^3^ Department of Urology Heilongjiang Provincial Hospital Heilongjiang China; ^4^ Department of Urology Peixian People's Hospital Jiangsu China; ^5^ Department of Pathology, Laboratory of Clinical and Experimental Pathology Xuzhou Medical University Xuzhou Jiangsu China; ^6^ Department of Genetics Xuzhou Engineering Research Center of Medical Genetics and Transformation, Key Laboratory of Genetic Foundation and Clinical Application Xuzhou Medical University Xuzhou China; ^7^ Department of Pathology Xuzhou Central Hospital Xuzhou Jiangsu China; ^8^ Department of Urology Huashan Hospital Fudan University Shanghai China; ^9^ Department of Urology Shanghai East Hospital, Tongji University School of Medicine Shanghai China

**Keywords:** bladder cancer, fibroblast cell, single‐cell sequencing, spatial transcriptome, tumour recurrence

## Abstract

**Background:**

Recurrent bladder cancer is the most common type of urinary tract malignancy; nevertheless, the mechanistic basis for its recurrence is uncertain. Innovative technologies such as single‐cell transcriptomics and spatial transcriptomics (ST) offer new avenues for studying recurrent tumour progression at the single‐cell level while preserving spatial data.

**Method:**

This study integrated single‐cell RNA (scRNA) sequencing and ST profiling to examine the tumour microenvironment (TME) of six bladder cancer tissues (three from primary tumours and three from recurrent tumours).

**Findings:**

scRNA data‐based ST deconvolution analysis revealed a much higher tumour heterogeneity along with TME in recurrent tumours than in primary tumours. High‐resolution ST analysis further identified that while the overall natural killer/T cell and malignant cell count or the ratio of total cells was similar or even lower in the recurrent tumours, a higher interaction between epithelial and immune cells was detected. Moreover, the analysis of spatial communication reveals a marked increase in activity between cancer‐associated fibroblasts (CAFs) and malignant cells, as well as other immune cells in recurrent tumours.

**Interpretation:**

We observed an enhanced interplay between CAFs and malignant cells in bladder recurrent tumours. These findings were first observed at the spatial level.

## Graphical abstract

- Our results provide the first transcriptomics‐based cell–cell interaction landscape of recurrent bladder cancer without losing spatial information.
- ST deconvolution analysis revealed a much higher tumour heterogeneity in recurrent tumours.
- Stronger interactions between epithelial cells and CAFs could be considered as a prognosis indicator for recurrent bladder tumours.
- Increased ligand–receptor interaction, such as COL4A1–SDC1 interaction activity, was identified between CAFs and other immune cells in recurrent bladder tumours.

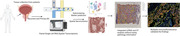

## 1 INTRODUCTION

Bladder cancer (BLCA), one of the most prevalent malignancies of the urinary tract, is a leading contributor to fatalities linked to cancer globally.^1^ The majority (75%) of BLCA cases are non‐muscle invasive and can be resected transurethrally as a treatment strategy.^2^ However, there is a high chance of recurrence (50%−70%) and progression into muscle‐invasive BLCA (20%).^3^, ^4^ Currently, the mechanism of BLCA development and recurrence remains unclear; therefore, it is necessary to further explore its pathogenesis at the cellular level.

Using single‐cell RNA sequencing (scRNA‐seq), researchers may analyse transcriptomes at the single‐cell level, explore the diversity of cells within the tumour microenvironment (TME) and observe cellular interactions.^5^, ^6^, ^7^ Using this, Oh et al.^8^ revealed heterogeneity in known CD4+ T cells in BLCA tissues. The scRNA‐seq analysis highlighted cytotoxic CD4+ T cells exhibiting tumour‐specific states, clonally expanding in tumours and presenting lethality to autologous tumours. Another scRNA‐seq analysis performed in BLCA also uncovered the underlying cellular heterogeneity, as indicated by the prominent expression of a pro‐tumourigenic inflammation signature against antitumour immunity in myeloid phagocytic cells. The TME's cellular diversity in BLCA is linked to PD‐1/PD‐L1 resistance.^9^ With regard to BLCA recurrence, Wang et al.^10^ identified an enriched subpopulation of cancer stem cells with elevated EZH2 expression and elucidated different epithelial–mesenchymal transition features in BLCA subtypes.

Although scRNA‐seq provides transcriptional information based on individual cells, the spatial cellular information is typically disrupted when tissue samples are homogenised before being sequenced, thereby neglecting the organisation and interaction between cells in their native tissue landscape. Under such circumstances, spatial transcriptomics (ST) is adopted to resolve the loss of spatial information. The histological context of the area of interest might be best retained using spatial mapping approaches,^11^ which could help locate different functional regions inside the tumour and evaluate changes in stromal or immune cells inside and outside the tumour.^12^ Combining ST with scRNA‐seq aids in visualising thousands of genes across a tissue section and mapping analytes in their physical location, hence making it possible to analyse the interactions between single cells.^13^ Notably, Gouin et al.^14^ applied scRNA‐seq with ST analysis in BLCA and identified that cadherin 12‐enriched bladder tumours exhibited a poor therapeutic response to neoadjuvant chemotherapy but greater sensitivity to immune checkpoint treatment. Although scRNA‐seq in conjunction with ST is crucial for a profound comprehension of recurrent BLCA as well as for differentiating between primary and recurrent BLCA, our insights into the molecular landscape of recurrent BLCA are currently lacking.

We sought to analyse whole‐transcriptome data across tissue sections among individuals with recurrent BLCA in the current investigation. This research could advance biomarker‐guided surveillance and management of individuals with recurrent BLCA and contribute to a better comprehension of the pathogenic mechanism of the disease.

## 2 MATERIALS AND METHODS

### 2.1 Patient sample collection

The research was conducted at Xuzhou Central Hospital's Urology Surgery Department (Xuzhou, China). Six participants with BLCA were recruited for the study from June 2021 to August 2021. Full‐layer bladder tumour tissues were obtained from BLCA patients who had undergone partial cystectomy or laparoscopic radical cystectomy. Postoperatively, the tissues were thoroughly washed with sterile normal saline before being placed in precooled MACS tissue storage solution at 4°C for analysis. The Xuzhou Central Hospital's Ethical Committee approved all the protocols (EC. XZXY‐LI‐20200708‐024). Additionally, written informed consent was obtained from each subject.

### 2.2 Single‐cell RNA‐seq sequencing experiment

To prepare the bladder tissues for scRNA‐seq, they were dissociated into single‐cell suspensions. After dissection, cold Dulbecco's phosphate‐buffered saline (PBS, Gibco) was used to wash the tissues three times before being cautiously transferred to digesting buffer (Dulbecco's modified Eagle medium [DMEM], 1.5 mL; trypsin, .5 mL; .9 U dispase, .5 mL; 2 mg/mL collagenase I, 1 mL; 2 mg/mL collagenase II, 1 mL; preheated to 37°C). After being cut into the proper small fragments in the digesting buffer, the tissues were gently shaken for 15 min in a shaker with a metal heater set at 37°C. Then, 10 μL of the resuspension was collected following the treatment, and a hemocytometer was used to observe the results. After filtering the cell suspension through a 70 μm mesh filter, 5 mL of DMEM was used to rinse the filter. The cells were concentrated by centrifugation (500 × *g*, 5 min at 4°C), and the supernatant was removed. Afterward, 50–100 μL DMEM with 10% PBS was used to resuspend the cells. Before loading the cells onto a 10× Genomics Chromium machine, the cell density was set to 700−1200 cells/μL. To accomplish reverse transcription for barcoding, Gel Bead‐In Emulsions were generated and obtained, and magnetic beads were employed to purify the first‐strand cDNA. The cDNA was utilised to construct the library utilising the 10× Chromium Single Cell 3′ Reagent kit's standard technique following quality control and measurement (v3, 10× Genomics, Pleasanton). The Illumina NovaSeq platform was utilised to sequence the library.

### 2.3 Processing and annotating scRNA‐seq data

By employing Cell Ranger, the clean reads that had been demultiplexed were aligned against the UCSC human GRCh38 reference genome. For downstream analysis in the R program (v4.1.2), we utilised Seurat v4.0^15^ after acquiring the single‐cell gene expression count matrix. Genes with low expression and possible noisy cells were initially eliminated for data quality control using multiple criteria, such as a maximum expression of 300 genes for each cell and a mitochondrial read percentage of >10%. To align all cells passing quality control, we utilised Seurat canonical correlation analysis‐based integration to identify the anchor genes across several data batches. Each matrix's top 3000 variable genes were chosen and imported into Seurat's *FindIntegrationAnchors* function. During integration analysis, the expression value of each gene was normalised by the *SCTransform* function, followed by the *IntegrateData* function. Analysing the integrated data using principal component analysis (PCA) reduced the dimension. To provide a two‐dimensional depiction of the cellular states, a scaled matrix (containing only the most variable genes) was subjected to uniform manifold approximation and projection (UMAP) dimensional reduction analysis.^16^ The *FindClusters* function was subsequently employed to cluster the cells utilising the shared closest neighbour modularity optimisation‐based clustering approach, which has a resolution of .6. Following the detection of the top hits per cluster, annotation of the types of cells was done using several reference panels from the deCS correlation analysis^17^ including BlueprintEncode, human cell landscape and monacoImmune, followed by canonical marker gene validation from the literature.^18^, ^19^, ^20^

### 2.4 Detection of cluster‐specific genes

The Seurat *FindAllMarkers* function was used to conduct the Wilcoxon rank‐sum test for the assessment of differentially expressed genes (DEGs) across various cellular clusters. A DEG was defined as one whose expression was above 25% in every cluster, with a log2 fold change (FC) >.25 relative to the background, and a false discovery rate <.05. In addition, for each cell type, DEG analysis between primary and recurrent tumours was conducted using the *FindMarkers* function. All the parameters were set as default. The UpSetR program was used to determine the DEG distribution.^21^

### 2.5 Functional enrichment analysis

The WEB‐based Gene Set Analysis Toolkit (WebGestalt R) (v0.4.4)^22^ was applied to conduct a functional enrichment analysis of DEGs. All human protein‐coding genes were used as background gene sets. Multiple test corrections were made utilising the Benjamini–Hochberg method.^23^ An adjusted *p*‐value <.05 was set as the criterion for significant pathways.

### 2.6 Verifying potential tumour cell clusters using InferCNV

To explore large‐scale chromosomal copy number variations (CNVs), we randomly down‐sampled 500 cells for each cell type. The raw expression matrix was used to run InferCNV (InferCNV of the Trinity CTAT Project, https://github.com/broadinstitute/inferCNV). Five different immune cell types were chosen from the primary tumours and served as reference groups. The cutoff for running InferCNV was set as .1, according to the author's guide for running 10× Genomics single‐cell data.

### 2.7 Cell–cell communication analysis

We used the R program CellChat^24^ to infer different cell‐to‐cell interplay across primary and recurrent cancers, thus detecting and visualising cell‐state specific cell–cell interplay.

Briefly, in compliance with the official procedure, we entered the standardised counts into CellChat and followed the standard preprocessing steps, including the functions *identifyOverExpressedGenes*, *identifyOverExpressedInteractions* and *projectData*, with standard parameter sets. The priori network information was selectively drawn from 2021 pre‐validated ligand–receptor (L–R) interplays. We used the functions *aggregateNet*, *computeCommunProbPathway* and *computeCommunProb* to calculate the information flow strength and communication probability between different cell groups for each L–R pair.^24^ A three‐dimensional tensor P and (*K* × *K* × *N*) was created by combining the total communication probabilities between all paired cellular groups throughout all L–R interplay, wherein *K* denotes six distinct cell types and *N* denotes L–R pairings or signalling pathways.^24^ To determine whether there would be substantial intercellular crosstalk between the primary and recurrent tumour groups, we subjected each L–R pair to a one‐sided permutation test (*n* = 100), which determines the probability that two cell groups will communicate by randomly permuting the group labels of the cells.^24^ A *p*‐value <.05 was chosen to denote the significance threshold.

### 2.8 Spatial transcriptome processing and annotation

Six BLCA patient capture areas were printed on ST slides (same patients from scRNA‐seq). The 10× Genomics Visium Spatial Gene Expression platform was used to record the gene expression data for ST slides, with the default technique employing mRNA‐binding oligonucleotides with spatial barcoding. Quality‐checking and mapping were conducted on the ST raw sequencing reads and Space Ranger (v1.3) was applied to align the demultiplexed clean reads to the UCSC human GRCh38 reference genome. To perform the downstream analysis on the R program (v4.1.2), we utilised Seurat v4.0 after acquiring the single‐cell gene expression count matrix. To align all spots from different individuals, we utilised the same method as described above for the scRNA‐seq data section, including *FindIntegrationAnchors*, *SCTransform* and *IntegrateData* functions. The integrated data were dimensionally reduced using PCA. The ‘RunUMAP’ function was subsequently employed to execute UMAP with the top 30 PCA components. The ‘FindClusters’ function was applied to perform barcoded spot clustering, with the resolution set at .2. For the prediction of the major cell type for each spot, we performed label transfer by utilising the *FindTransferAnchors* and *TransferData* functions. In addition, we conducted a parametric analysis of gene set enrichment (PAGE)^25^ between the gene signatures of each type of cell and the pattern of expression for every spatial location using the Giotto suite.^26^ The cell‐type deconvolution results were further submitted to SPOTlight^27^ for visualisation. Finally, gene imputation was conducted using the Bayesian statistical model in BayesSpace.^28^

### 2.9 Spatial proximal and communication analysis

To identify cell types that were selectively situated in a spatially close location, we used a random permutation strategy implemented in the Giotto Analyser's *cellProximityEnrichment* function. In addition, we applied the Giotto *spatCellCellcom* function to identify if a pair of genes are expressed at a higher level than expected depending on a reshuffled null distribution of the gene expression levels in cells that are spatially in proximity to each other. Interactions with an adjusted *p*‐value <.05 and abs(log2 FC) >.1 were judged significant. To identify the proximal and communication pattern differences between primary and recurrent tumours, we applied a *t*‐test to compare the PI significance score for each pair of cell types (proximity enrichment) or genes (L–R communication), defined as log2 FC × –log_10_ (adjusted *p*‐value). A *p*‐value <.05 was established as the significance criterion. Nichenet was also used to validate the interplay across various types of cells.^29^ In addition, we utilised the CellTrek package (Delaunay triangulation approach)^30^ to co‐embedding the scRNA‐seq data at the spatial level and further investigate the colocalisation patterns between different cell types.

### 2.10 Multiplex immunofluorescence experiment

We performed the multiplex immunofluorescence experiments by using the CD8 anti‐human antibody (Ab) (ab101500), EPCAM anti‐human Ab (Abcam, ab223582), cytokeratin‐8 anti‐human Ab (Abcam, ab53280), cytokeratin‐19 anti‐human Ab (Abcam, ab76539), collagen‐IV (COL4A1) anti‐human Ab (Abcam, ab214417), SDC1 anti‐human Ab (Abcam, ab128936), CD3 anti‐human Ab (Abcam, ab16669), CD56 anti‐human Ab (Abcam, ab75813), CCL22 anti‐human Ab (Abcam, ab23772) and 4′,6‐diamidino‐2‐phenylindole staining solution (Abcam, ab228549). Following the manufacturer's instructions (Akoya, Opal Polaris 7 Color Automation IHC Detection Kit), we scanned the slides with the Akoya Vectra Polaris Automated Quantitative Pathology Imaging System and quantified the results by using Akoya Inform software.

## 3 RESULTS

### 3.1 The transcriptomic heterogeneity of bladder tumour is shown by single‐cell sequencing

**FIGURE 1:**
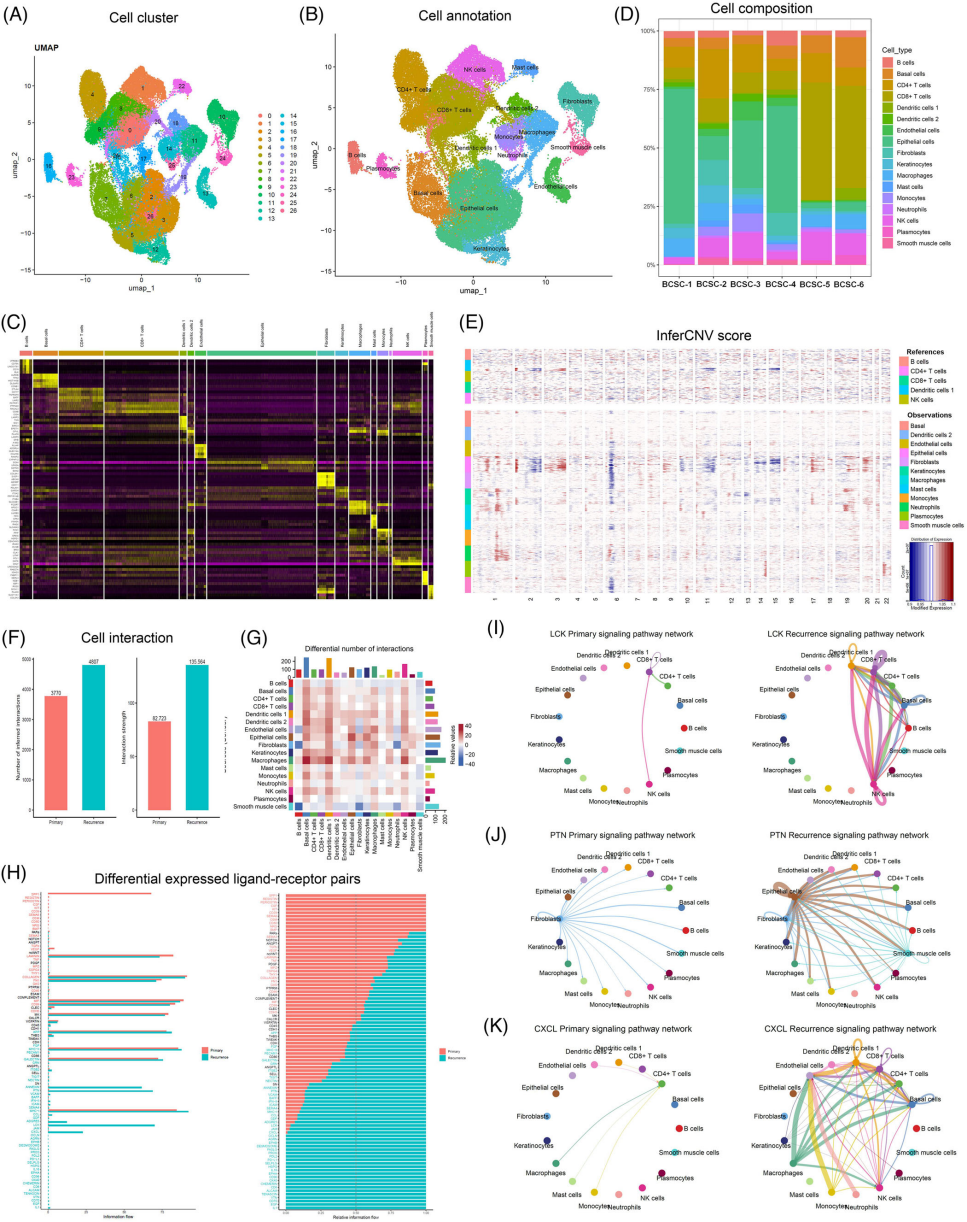
Single‐cell RNA sequencing integrative and communication analysis between primary and recurrent bladder tumour. (A) Scheme of this integrated omics study. BC: bladder cancer; ST: spatial transcriptomics. (B) Uniform manifold approximation and projection (UMAP) and 27 cell clusters were annotated into (C) heatmap of canonical marker gene expression across 17 major cell types. (D) The relative composition of cell types in each primary and recurrent tumour. (E) Copy number variation analysis between recurrent and primary bladder tumours. (F) The predicted cell–cell crosstalk networks between primary and recurrent tumours are depicted in a bar plot along with the total number of interactions and interaction strength. (G) Heatmap displaying the general flow of information of diverse interactions across various types of cells. The total of the values shown in the heatmap's columns is depicted by the coloured bar plot above (incoming signalling). The total of the row of values is depicted by the right‐coloured bar plot (outgoing signalling). (H) Top representative differentially expressed (DE) ligand–receptor signalling pathways between primary and recurrent tumour. (I–K) Circle plots showing three representative DE ligand–receptor signal pathways between primary and recurrent tumours.

To study the cell diversity and transcriptomic heterogeneity between primary and recurrent bladder tumours, we collected tissues from six patients (Table 1) and performed paired 10× single‐cell seq and spatial transcriptome seq using the 10× Genomics platform. The study scheme is described in Figure 1A. According to the patient's clinical classification, the tissues were divided into two subgroups: primary tumours and recurrent tumours (details in Section 2). We screened and processed the data before performing the single‐cell transcriptome analysis. After batch integration, 59 858 cells with an average of 6947 UMI (2041 features) were collected for downstream reduction of dimensionality and cell clustering. UMAP was used to visualise the data. This analysis identified 27 initial cell clusters (Figure 1B). Next, we applied the deCS software and canonical marker genes from the literature to assign these clusters to 17 major cell clusters (Figure 1C). Diverse cell clusters were detected, including lymphoid lineage: B cells (*VPREB3*), plasma cells (*CD79A*), CD4^+^ and CD8^+^ T cells (*PTPN22*) and natural killer (NK) cells (*GNLY*); myeloid lineage: dendritic cells (DCs) (*LAMP3*), macrophages (*APOC1*), monocytes (*FCN1*) and neutrophils (*EREG*); and epithelium lineage: basal cells (*CENPA*), epithelial cells (*SOX4*) and keratinocytes (*ITGA2*). According to the UMAP coordinates, we noticed that lymphoid lineage cell types, notably NK cells and CD4^+^ and CD8^+^ T cells, were grouped; myeloid lineage macrophages, monocytes, neutrophils and DCs were grouped; and basal cells, epithelial cells and keratinocytes were grouped. In addition, we identified many fibroblasts (*ABCA8*), smooth muscle cells (*RGS5*), endothelial cells (*PLVAP*) and mast cells (*HDC*). The fibroblast and smooth muscle cells were also relatively close to UMAP. These results support the accuracy of the proposed clustering and annotation methods.

**TABLE 1:** Clinical information of patients included in this study and summary of single‐cell RNA sequencing and spatial transcriptome data.

Sample	Status	Gender	Age	Organisation type	Incidence of bladder cancer	Pathological type (high grade)	Notes	Operative method	scRNA‐seq/ST name	Cell counts	Spot counts	Batch
Bladder cancer 1	Primary	Male	65	Bladder carcinoma tissues and para‐carcinoma tissues	Emerging cancer	High‐grade invasive urothelial carcinoma	High‐grade invasive urothelial carcinoma has severe local tissue compression	Partial cystectomy	BLSC1/ST1	14105	3544	1
Bladder cancer 2	Primary	Male	70	Bladder carcinoma tissues and para‐carcinoma tissues	Emerging cancer	High‐grade invasive urothelial carcinoma	High‐grade invasive urothelial carcinoma of the bladder with more eosinophil infiltration in the stroma	Laparoscopic radical cystectomy	BLSC2/ST2	10070	4134	2
Bladder cancer 3	Primary	Male	77	Bladder carcinoma tissues and para‐carcinoma tissues	Emerging cancer	High‐grade invasive urothelial carcinoma	High‐grade invasive urothelial carcinoma of the bladder, invading the muscularis propria	Laparoscopic radical cystectomy	BLSC3/ST3	10601	4271	3
Bladder cancer 4	Recurrent	Female	55	Bladder carcinoma tissues and para‐carcinoma tissues	Recurrent cancer	High‐grade invasive urothelial carcinoma	High‐grade papillary urothelial carcinoma of the bladder, invading the whole layer of the bladder, invading the myometrium, vascular and nerve invasion, and cancer invasion of the right ureter and ovary	Laparoscopic radical cystectomy	BLSC4/ST4	8002	4421	1
Bladder cancer 5	Recurrent	Female	68	Bladder carcinoma tissues and para‐carcinoma tissues	Recurrent cancer	High‐grade invasive urothelial carcinoma	High‐grade invasive urothelial carcinoma of the bladder, size 3.2 × 2.5 × 1.5 cm, infiltrating the whole layer of bladder wall, nerve (–), vessel (–), sending ureteral margin (–), immunohistochemistry: CK7 (+), CK20 (–), S100P (+), p53 (+), CD44 (+), CK5/6 (+), GATA3 (–), Ki‐67 (60%)	Laparoscopic radical cystectomy	BLSC5/ST5	6796	4589	2
Bladder cancer 6	Recurrent	Male	77	Bladder carcinoma tissues and para‐carcinoma tissues	Recurrent cancer	Poorly differentiated bladder adenocarcinoma	Poorly differentiated bladder adenocarcinoma, invading the muscularis propria	Partial cystectomy	BLSC6/ST6	10284	4518	3

We noted some cell subpopulations that had a strong preference for batches, even though UMAP analysis illustrated that the batch impacts were negligible among the three distinct batches (Figures 1D and S1); for example, most detected epithelial cells were enriched in batch 1 (1 and 4) and CD8^+^ T cells in batch 3 (3 and 6). Therefore, the relative proportion of broad cell type changes in three different batches was assessed independently. As depicted in Table S1, during the immune cell type analysis, we observed that CD4^+^ T cells and type‐2 DCs decreased, while CD8^+^ T cells and plasmacytes significantly increased in all three batches of recurrent bladder tumours, indicating an expected stronger immune response in recurrent tumours. In addition, we found that the relative abundance of epithelial cells and keratinocytes decreased in all three batches of recurrent tumours, while the relative abundance of basal cells increased by 1.51–3.39‐fold. Based on CNV analysis, we applied InferCNV to verify that the epithelial cells have the strongest CNV alterations, suggesting a limited presence of normal epithelial cells (Figure 1E); therefore, we treated epithelial and tumour cells as synonymous during downstream analysis. Figure S2 presents a thorough summary of cancer‐related cell types. However, a few common features were observed among the three batches of recurrent tumours, indicating a highly heterogeneous nature of cancer cells among different patients.

Next, we compared the DEGs between the primary and recurrent tumours. Collectively, we identified 2699 DEGs among 17 cell types (median 483), especially in NK cells (914) and CD4^+^ (857) and CD8^+^ (813) T cells. Among these genes, 2208 of 2699 showed differential expression levels in at least two cell types. In addition, the regulation of 1060 DEGs varied in opposing directions among distinct cell types. Therefore, we also produced two distinct UpSetR plots to display the number of common or cell type‐specific down‐ and upregulated genes (Figure S3). Furthermore, pathway enrichment results of the upregulated genes in recurrent tumours demonstrated strong cell‐type specificity (Figure S4). For example, the upregulated genes in basal and endothelial cells showed primary enrichment in ‘actin binding’, while those in NK cells were primarily enriched in ‘cytokine receptor binding’. However, some pathways enriched in upregulated genes were also enriched in downregulated genes in multiple cell types. Therefore, we suspect that the transition of primary and recurrent tumours comprises a fine‐tuned cascade of interactions across multiple types of cells.

### 3.2 Difference between cell–cell interaction analysis of primary and recurrent bladder tumour tissues

Next, we investigated the cellular communication differences to reveal dysregulated pathways between primary and recurrent tumours. Figure 1F demonstrates that in recurrent bladder tumours, both the total number and the strength of cellular interplay increased. As depicted in the results of Figure 1G, most differential interactions were detected in incoming signalling of basal cells, type‐1 DCs (plasmacytoid DCs), and NK cells and outgoing signalling of macrophages, endothelial cells and epithelial cells. For each pair of L–R signalling pathways, we also evaluated the flow of information. A total of 92 L–R pairs were dysregulated in recurrent tumours (Table S2). The majority of the signalling pathways implicated in inflammatory and immune responses were remarkably upregulated in recurrent tumours, as illustrated in Figure 1H. For example, the *LCK* pathway is activated among basal cells, T cells and NK cells in recurrent tumours (Figure 1I). A recent report demonstrated that *LCK* is implicated in the activation of T‐cell receptor signalling in both naive and effector T cells. Similarly, it has been demonstrated that *PTN* expression induces leukocyte response by causing their migration through the expression of inflammatory cytokines,^31^ demonstrating a stronger communication pattern in recurrent epithelial cells (Figure 1J). In addition, *CXCL1*, which has a role in modulating immunological and inflammatory reactions, was significantly enhanced in recurrent macrophages and endothelial cells (Figure 1K). Nevertheless, given that proximity is required for the majority of physical interactions of two or more types of cells, scRNA‐seq data at the transcriptomic level may not provide a fully accurate view of intercellular crosstalk.

### 3.3 Spatial transcriptomics resolved the transcriptomic heterogeneity in bladder tumours

**FIGURE 2:**
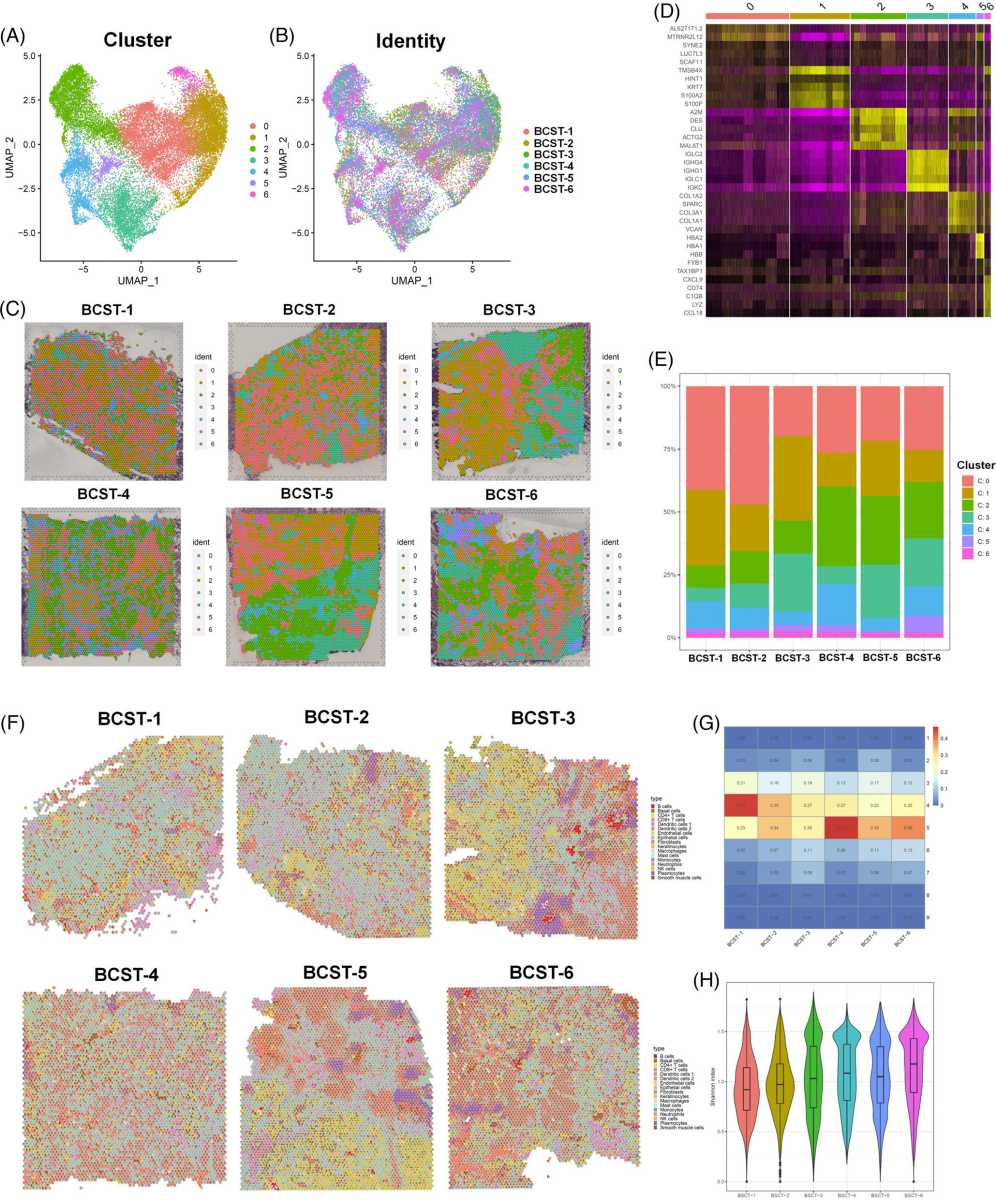
Basic and deconvolution analysis of 10× Visium spatial transcriptome of primary and recurrent bladder tumour. (A and B) Our integrative methodology effectively overcomes batch effects, as shown by the uniform manifold approximation and projection (UMAP), seven‐point clusters and UMAP breakdown according to sample origin. Bladder cancer spatial transcritpome (BCST) 1−3: primary, 4−6: recurrent. (C) Spatial localisation of individual clusters among different samples. (D) Heatmap of canonical marker gene expression across seven spot clusters. (E) The relative cell‐type composition of each primary and recurrent tumour. BCST 1−3: primary, 4−6: recurrent. (F) Pie chart visualisation of the cell type proportions as sections of a for each spot. (G) The percentage of cell type contributed to each spot among each spatial transcriptome. (H) α‐Diversity (Shannon index) comparison between primary and recurrent bladder tumour at the spot level.

scRNA‐seq offers transcriptomic profiles of highly heterogeneous cell populations and makes it easier to find genes that distinguish different cell subtypes. This new ST technology enables us to examine the patterns of spatial gene expression in tissues and characterise local structures and microenvironments, thereby mediating the detection of cell–cell interplay across different spatial locations. Using 10× Visium ST technology, we generated 25 477 barcoded spots with an average of 6230 UMI (2148 features) among six spatial samples. For a better representation of the spatial heterogeneity of primary and recurrent bladder tumours at the spatial level (Table 1), we conducted data preprocessing and batch effect removal using Seurat v4.0 anchor‐based integration (see details in Section 2). After unsupervised clustering of spatially barcoded spots and UMAP visualisation, seven different clusters were identified among the six different samples (Figure 2A,B). Subdividing the total pool of spots into six individual ones based on donor identity showed that all clusters appeared in all the samples (Figure 2C), indicating that batch effects had been minimised. To detect region‐specific marker genes, we performed DEG analysis (Figure 2D) and applied Giotto PAGE (integrative analysis of region‐specific marker genes and cell type‐specific genes from scRNA‐seq) to annotate the dominant cell type within each spot cluster. Detailed results are shown in Table S3 and Figure S5. For each cluster, we also presented the top marker gene at the histological image level (Figure S6). Additionally, we discovered an elevation in the proportion of C2 cells (fibroblasts and smooth muscle cells) in all three recurrent tumours (Figure 2E), indicating a more complex microenvironment.

### 3.4 Spatial transcriptome deconvolution analysis reveals recurrent tumours with higher heterogeneity

To quantify the cell type contribution within each spot, we merged our ST data with the scRNA‐seq data using Seurat v4.0 anchor‐based integration^32^ since 10× Visium ST cannot provide single‐cell resolution. First, the Seurat anchor method finds pairwise correspondences between the elements in the two datasets as anchors between the datasets. These components seem to originate from the same biological state. Each location in the spatial data was seen as a weighted mixture of the various cell types discovered by scRNA‐seq. Therefore, to determine weights for every type of cell resulting from scRNA‐seq for each site, we employed the label transfer prediction scores. For a better understanding, we generated the prediction scores of the target cell types with aligned histology images, as shown in Figure 2F. As shown in Supporting Information S1, two to seven cell types contributed a total of 99.5% transcriptome spots (prediction score >1% as a positive contribution). As expected, most spots annotated as epithelial cells, keratinocytes, and fibroblasts overlapped significantly with tumour cells (Figure S7). A network graph and heatmap representing spatial interactions where cell types with stronger edges between them are more often found within the same spot were also generated (Figures S8 and S9). As shown in Figures S8 and S9, the epithelial cells, keratinocytes, fibroblasts, smooth muscle cells and CD8^+^ T cells demonstrated strong consistency. Additionally, we noted that a certain number of CD8+ T cells and plasmacytes were proximal to the tumour boundary, indicating an increased immune response in recurrent tumours. In contrast, only a few transcriptome spots were found to be dominated by other immune types, including monocytes, DCs and mast cells. This is consistent with their low abundance at the single‐cell level.

We further studied local cell‐type heterogeneity (number of contributed cell types) between the primary and recurrent tumours. As illustrated in Figure 2G, we found that the local cell‐type heterogeneity was greater (>5‐cell types) in all three recurrent tumours (56.72%) than in primary tumours (40.96%, *p*‐value = .026, *t*‐test) (Table S4). We also examined their relative compositions. The recurrent group's Shannon diversity index was remarkably larger relative to that of the primary group, as depicted in Figure 2H. We presume this to be due to the higher heterogeneity and stronger immune response in recurrent tumours. Overall, it was possible for us to spatially clarify cell type‐specific gene expression in BLCA because of the integration of scRNA‐seq and ST. Deconvolution analysis reveals that spots of recurrent tumours are of overall higher heterogeneity.

### 3.5 Spatial proximity analysis reveals enhanced cell–cell interplay between epithelial/basal cells and NK and T cells in the recurrent group

**FIGURE 3:**
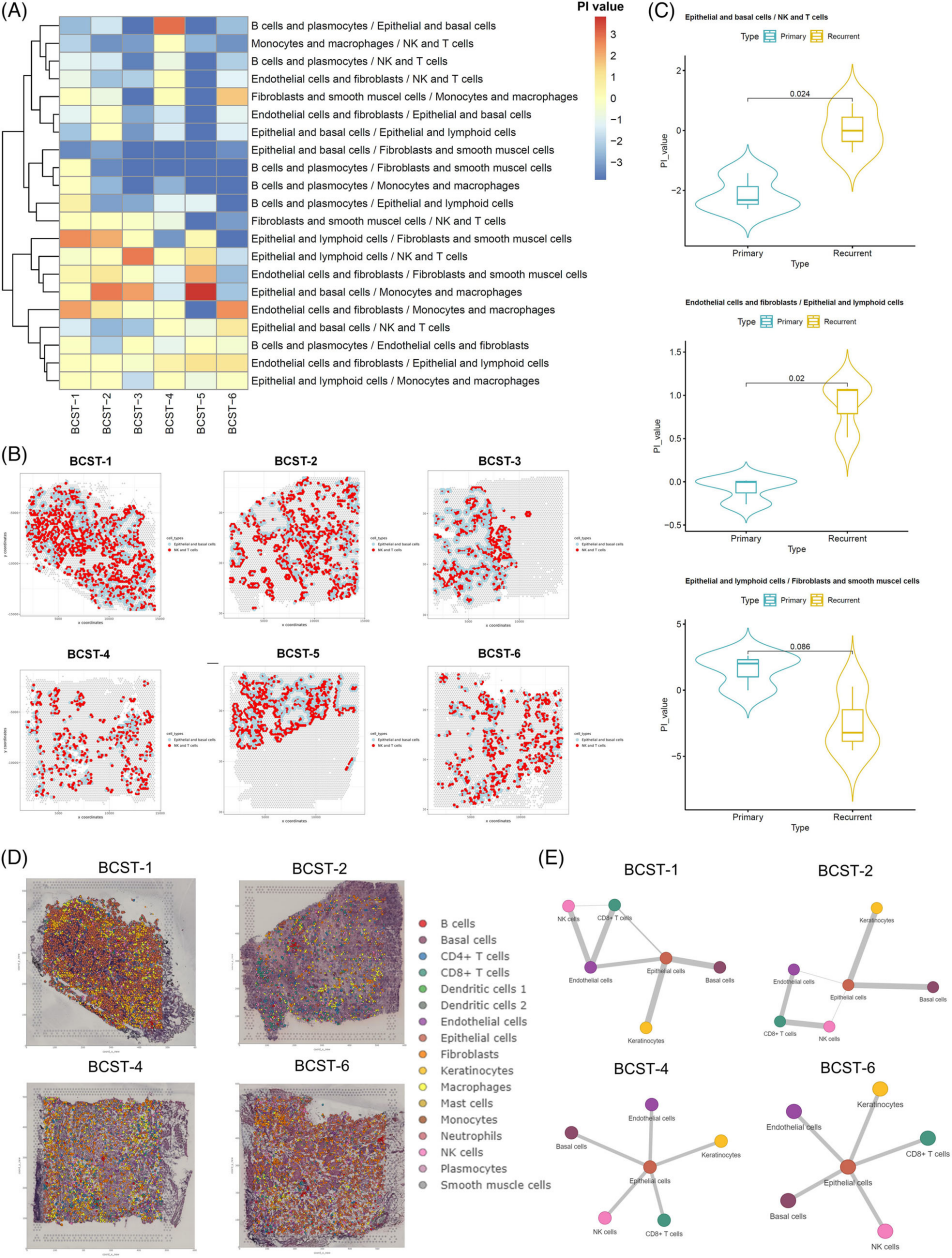
Cell–cell proximate comparison between primary and recurrent bladder tumour. (A) Heatmap of cell–cell proximity enrichment score of seven spot clusters among six samples. The PI value was defined as log2 fold change (FC) × –log_10_ (adjusted *p*‐value), simulated by Giotto *cellProximityEnrichment* function. (B) Violin plot of the PI value between the pair of epithelial and basal cells/natural killer (NK) cells and T cells, endothelial cells and fibroblasts/epithelial and lymphoid cells, epithelial and lymphoid cells/fibroblasts, and smooth muscle cells. (C) Cell–cell proximity visualisation of C0: NK and T cells and C1: epithelial and basal cells, all six patients NK, T cell and epithelial basal cell–cell were labelled by red and blue colour, plotted by Giotto cellProximitySpatPlot2D function. (D) Co‐embedding analysis of single‐cell and spatial transcriptomes by CellTrek. (E) Spatial colocalisation analysis of NK cells, T cells and epithelial cells by Delaunay triangulation approach. BCST 1 and 2: primary, 4 and 6: recurrent.

Cell‐type proximity maps may be employed to direct the identification of the crosstalk between cell types of the same or distinct lineages since proximity is required for two or more cells to engage in physical interaction. First, by comparing the observed frequency of cell–cell proximity interplay to the predicted frequency for every sample, the spatial proximity enrichment or depletion between distinct paired cell types was determined (Figure S10). Interestingly, spatially proximal differential analysis of cell types revealed that the interaction between C1 (epithelial and basal cells) and C0 (NK and T cells) was enhanced in the recurrent group as opposed to the primary group (Figure 3A and Table S5), while the content and ratio versus total spots of both C1 and C0 were lower in the recurrent group. This result is consistent with the single‐cell level results, demonstrating that recurrent cancers exhibit a considerable increase in immune and inflammatory signalling pathways (Table S1). ST results provide visible evidence for these cell–cell interactions (Figure 3B). In addition, we observed an interaction between C4 (fibroblasts and endothelial cells) and C5 (epithelial and lymphoid cells) that was enhanced in the recurrent group. However, the interaction between C5 (epithelial and lymphoid cells) and C2 (fibroblasts and smooth muscle cells) was decreased in the recurrent group (Figure 3C). To validate our findings, we further investigated the spatial colocalisation patterns of distinct cellular types by applying CellTrek,^30^ based on scRNA‐seq and ST data. We confirmed the upregulated colocalisation of epithelial cells, keratinocytes and basal cells (Figure 3D,F) with NK and CD8^+^ T cells in the recurrent group, implying more complex tumour microenvironments in the recurrent group.

**FIGURE 4:**
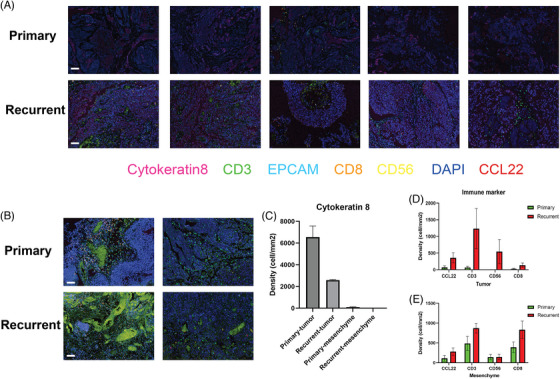
Validation of increased natural killer (NK)/T and epithelial/basal cell–cell interactions by multiplex immunofluorescence experiments. (A) Featured scanned slides of tumour regions of primary and recurrent tumours. Each slide corresponds to tissue samples from a distinct individual. *N* = 5. The indicated colours represent different markers. Bar = 50 μm. (B) Featured scanned slides of mesenchyme of primary and recurrent tumours. (C) Density quantification of cytokeratin‐8 for determining tumour and mesenchyme regions. The Akoya Vectra Polaris Automated Quantitative Pathology Imaging System was used to scan the slides, and Akoya Inform software was used to quantify the results. *N* = 5. (D and E) Quantification results of NK/T‐cell population in the tumour and mesenchyme regions.

Analysis through different bioinformatic approaches presented an increased NK/T cell and putative tumour cell interaction in the recurrent group. To further validate our findings, we performed multiplex immunofluorescence assays on 10 (five primary and five recurrent) tumour samples to examine the colocalisation of NK/T cells and epithelial cells. We used markers that were highly expressed in our scRNA‐seq datasets, such as EPCAM, cytokeratin‐8 and cytokeratin‐19, to mark the epithelial and basal cells. CD3 and CD8 were used to represent CD8^+^ T cells, and CD56/CCL22 was used to represent NK cells and DCs. As expected, we found that the cell densities of CD3/CD8 and CD56/CCL22 were significantly higher in the tumour region of the recurrent group (Figure 4A). In the mesenchyme, there were more CD8+ T cells than NK cells in the recurrent group (Figure 4B). Cytokeratin‐8 was used as a marker to determine the tumour region and mesenchyme (Figure 4C). Quantitative analysis was also performed to show the difference in colocalisation of NK/T and epithelial/basal cells between primary and recurrent groups (Figure 4D,E). Overall, these immunofluorescence results further demonstrated the increased immune complexity in recurrent tumours.

### 3.6 Spatial analysis reveals increased genes involved in immune response and cell adhesion changes in recurrent tumours

By applying the same approach, we systematically investigated the L–R combinations among different samples, which led to the identification of 930–4987 significant L–R pairs among the six spatial samples (Table S6). However, only 45 and 57 common L–R interactions could be identified in either the primary or recurrent group (all three samples), respectively. The details are shown in Figures S11 and S12. Interestingly, we found that three spatially differentially expressed L–R combinations, including *FN1*, *THY1* and *SPP1*, overlap with scRNA‐seq data, indicating that scRNA‐seq and ST analysis work in coordination to identify significant L–R interactions. These two methods can be used to validate the results.

**FIGURE 5:**
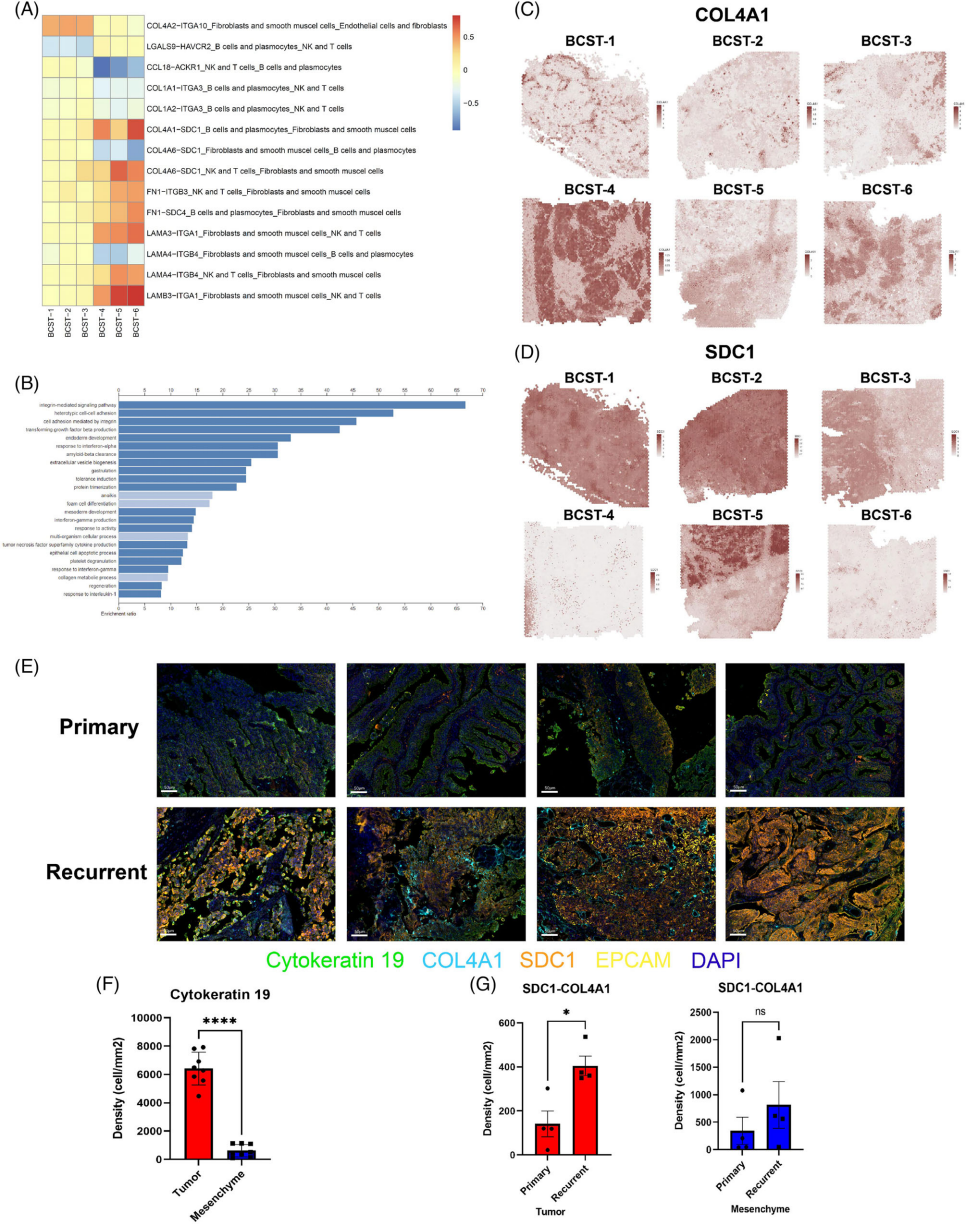
Spatial cell–cell communication differential analysis among seven spot clusters. (A) Fourteen spatially common differentially expressed ligand–receptor (L–R) signalling pathways between primary and recurrent bladder tumour. (B) Gene ontology functional enrichment analysis of spatially differentially expressed L–R genes. (C and D) Two representatively L–R genes (COL4A1 and SDC1) expression levels were imputed by BayesSpace. (E) Featured scanned slides of tumour regions of primary and recurrent tumours. The indicated colours represent different markers. Bar = 50 μm. (F) Cytokeratin‐19 was used to distinguish tumour and mesenchyme regions. (G) Quantification results of SDC1 and COL4A1 colocalisation in tumour and mesenchyme. Student's *t*‐test was conducted. ^*^ *p* < .05; ns: not significant.

We identified the most significant spatial differentially expressed L–R interactions between the primary and recurrent tumours in Figure 5A. These L–R proteins were primarily involved in integrin‐mediated signalling cascades and heterotypic cell–cell adhesion, according to functional enrichment analysis (Figure 5B). For example, we identified a significant increase in the COL4A1 and SDC1 interactions in recurrent tumours, of which, the expression levels of the two representative genes (*SDC1* and *COL4A1*) are shown in Figure 5C,D. Both genes played a role in extracellular matrix organisation and were previously associated with multiple cancer progression and drug resistance.^33^, ^34^, ^35^ Indeed, multiplex immunofluorescence experiments were also performed and validated the increased colocalisation of SDC1 and COL4A1 in the recurrent group (Figure 5E), mainly in the tumour region (Figure 5F,G). Altogether, spatially resolved transcriptomic and imaging data show that gene interactions involved in immune response and cell adhesion are ubiquitously enhanced in recurrent bladder tumours, especially in cancer‐associated fibroblasts (CAFs).

## 4 DISCUSSION

To examine the TME of recurrent BLCA tissues, we conducted an integrated analysis of scRNA‐seq and spatial transcriptome profiling in this work. First, we identified that scRNA data‐based ST deconvolution analysis revealed much higher tumour heterogeneity in recurrent tumours. Second, we found that while the overall NK/T cell and malignant cell number or the ratio of total cells was similar or even lower in the recurrent tumours, the interactions between these two cell types were much stronger. Finally, increased L–R interaction between CAFs and other immune cells was observed in recurrent tumours.

Higher heterogeneity, characterised by higher complexity of immune and stromal cells, was noted in recurrent BLCA than in primary BLCA. It was previously indicated that a pre‐existing intratumoural heterogeneity was associated with chromosomal instability, tumour aggressiveness and drug resistance in various tumour types.^36^, ^37^, ^38^ This higher level of heterogeneity may result in a poor prognosis and may be passed on to recurrent tumour tissues. However, this has not been validated for BLCA or other tumour types. In previous studies, recurrent tumour samples generally shared similar non‐malignant cell (immune and stromal) subtypes with primary tumour samples but with different proportions and functional states.^39^, ^40^ Wang et al.^10^ found that recurrent BLCA tissues contain a similar number of immune cells as low‐risk BLCAs but fewer immune cells than high‐risk BLCAs. Using integrated ST and scRNA‐seq analysis, we were the first to observe this higher complexity at the individual spot level.

Although the quantity and percentage of intratumoural immune cells were similar between recurrent and primary BLCA samples, intercellular communication was significantly upregulated in recurrent BLCA, especially for the interplay between NK/T cells and malignant cells (basal and epithelial cell clusters). Such elevated interactions between cells were consistent with other scRNA‐seq studies of recurrent cancer, suggesting a coupled activated and dysfunctional state of the immune response in recurrent tumour tissues.^39^, ^40^ According to Peng et al.,^39^ recurrent nasopharyngeal carcinoma showed increased regulation of immune cells and an enhanced ability to shape the immune microenvironment. The interaction and chemotaxis between Tregs and CD8+ T cells are active in recurrent nasopharyngeal carcinoma. Activation‐related pathways, including JAK/STAT, phospholipase D and nuclear factor‐kappa B, were upregulated in recurrent tumour‐derived CD8+ T cells, along with exhaustion‐related transcription factors. Recurrent hepatocellular carcinoma also exhibited similar phenotypes, which were hallmarked by decreased proportions of immunosuppressive Tregs and increased proportions of innate‐like CD8+ T cells and DCs. Notwithstanding the impaired antitumour immune response, the immune ecosystem of recurrent hepatocellular carcinoma also demonstrates that recurrent malignant cells and immune cells are actively communicating with one another.^40^, ^41^ In this study, we used spatial‐based interaction analysis to show the increased physical interactions of NK/T cells and malignant cells in recurrent bladder tumour tissues directly on experimental slides, providing solid evidence to support the previous findings regarding BLCAs.

Our data showed that the interplay between CAFs and immune cells was upregulated in recurrent BLCA. Numerous studies have demonstrated that CAFs can promote cancer or impair the sensitivity to immunotherapy in many types of malignancies.^42^, ^43^, ^44^, ^45^ The TME's main component, CAFs, may trigger immune cell escape and chemoresistance and impact cancer stem cell renewal.^46^, ^47^ A higher level of CAF infiltration was observed in recurrent renal cell carcinoma, concurrent with the upregulated expression of immunosuppressors produced by CAFs.^47^ With regard to BLCA promotion, inflammatory CAF, which is known for its cytokine‐secreting properties, has proven to be a key factor and is functionally related to extracellular matrix (ECM) organisation and focal adhesion.^6^, ^48^ In the current study, COL4A1 and SDC1 performed fundamental functions in the interplay between CAFs and immune cells in recurrent BLCA. The interplay between ECM receptors and the focal adhesion pathway was influenced by both COL4A1 and SDC1.^49^, ^50^, ^51^, ^52^ SDC1 was previously linked to a worse prognosis for BLCA individuals, but the exact mechanism is still uncertain.^35^ Similarly, *COL4A1* and *SDC1* expression levels have previously been linked to a dismal prognosis among patients with ovarian cancer.^53^ *COL4A1* promotes the growth and metastasis of liver cancers^34^ and increases drug resistance in gastric cancer.^33^ Additionally, research suggested that COL4A1's activation of the PI3K/AKT pathway may result in the recurrence of gastric cancer.^54^ First, our ST findings showed that this L–R could be crucial in defining the BLCA TME. Future studies should concentrate on exploring the interactions between COL4A1‐positive fibroblasts and SDC1‐positive immune cells, which could ultimately contribute to the poor prognosis of BLCA. Moreover, significant CAF‐immune cell‐based L–R interactions were also observed in the recurrent BCs, while most of these L–R were in the ECM and cell adhesion pathways. These signalling pathways integrated various signalling molecules regulating cancer cell survival, proliferation and motility and were highly possible to be linked to the recurrence of BLCA.^55^, ^56^ Future research should focus on figuring out the biological significance of specific increased L–R interplay in recurrent cancers.

## CONFLICT OF INTEREST STATEMENT

The authors declare they have no conflicts of interest.

## Supporting information

Supporting Information

Supporting Information

Supporting Information

Supporting Information

Supporting Information

Supporting Information

Supporting Information

Supporting Information

Supporting Information

Supporting Information

Supporting Information

Supporting Information

Supporting Information

Supporting Information

## Data Availability

The raw data that support the findings of this study are deposited in the NGDC/BMDC dataset (Project number: OEP004141). The processed data have been included in the manuscript (Supporting Information Tables).

## References

[1] Parker J , Spiess PE . Current and emerging bladder cancer urinary biomarkers. ScientificWorld J. 2011;11:1103‐1112. 10.1100/tsw.2011.104 PMC5720052 21623456

[2] Kamat AM , Hahn NM , Efstathiou JA , et al. Bladder cancer. Lancet. 2016;388(10061):2796‐2810. 27345655 10.1016/S0140-6736(16)30512-8

[3] Lenis AT , Lec PM , Chamie K . Bladder cancer: a review. JAMA. 2020;324(19):1980‐1991. 33201207 10.1001/jama.2020.17598

[4] Babjuk M , Böhle A , Burger M , et al. EAU guidelines on non‐muscle‐invasive urothelial carcinoma of the bladder: update 2016. Eur Urol. 2017;71(3):447‐461. 27324428 10.1016/j.eururo.2016.05.041

[5] Ahmed R , Zaman T , Chowdhury F , et al. Single‐cell RNA sequencing with spatial transcriptomics of cancer tissues. Int J Mol Sci. 2022;23(6):3042. 10.3390/ijms23063042 PMC8955933 35328458

[6] Chen Z , Zhou L , Liu L , et al. Single‐cell RNA sequencing highlights the role of inflammatory cancer‐associated fibroblasts in bladder urothelial carcinoma. Nat Commun. 2020;11(1):5077. 33033240 10.1038/s41467-020-18916-5 PMC7545162

[7] Lai H , Cheng X , Liu Q , et al. Single‐cell RNA sequencing reveals the epithelial cell heterogeneity and invasive subpopulation in human bladder cancer. Int J Cancer. 2021;149(12):2099‐2115. 34480339 10.1002/ijc.33794

[8] Oh DY , Kwek SS , Raju SS , et al. Intratumoral CD4+ T cells mediate anti‐tumor cytotoxicity in human bladder cancer. Cell. 2020;181(7):1612‐1625.e13. 32497499 10.1016/j.cell.2020.05.017 PMC7321885

[9] Wang L , Sfakianos JP , Beaumont KG , et al. Myeloid cell‐associated resistance to PD‐1/PD‐L1 blockade in urothelial cancer revealed through bulk and single‐cell RNA sequencing. Clin Cancer Res. 2021;27(15):4287‐4300. 33837006 10.1158/1078-0432.CCR-20-4574 PMC8338756

[10] Wang H , Mei Y , Luo C , et al. Single‐cell analyses reveal mechanisms of cancer stem cell maintenance and epithelial–mesenchymal transition in recurrent bladder cancer. Clin Cancer Res. 2021;27(22):6265‐6278. 34526362 10.1158/1078-0432.CCR-20-4796

[11] Chen G , Ning B , Shi T . Single‐cell RNA‐seq technologies and related computational data analysis. Front Genet. 2019;10:317. 31024627 10.3389/fgene.2019.00317 PMC6460256

[12] Rantalainen M . Application of single‐cell sequencing in human cancer. Brief Funct Genomics. 2018;17(4):273‐282. 29106464 10.1093/bfgp/elx036 PMC6063300

[13] Abdelaal T , Mourragui S , Mahfouz A , Reinders M . SpaGE: spatial gene enhancement using scRNA‐seq. Nucleic Acids Res. 2020;48(18):e107. 32955565 10.1093/nar/gkaa740 PMC7544237

[14] Gouin KR , Ing N , Plummer JT , et al. An N‐cadherin 2 expressing epithelial cell subpopulation predicts response to surgery, chemotherapy and immunotherapy in bladder cancer. Nat Commun. 2021;12(1):4906. 34385456 10.1038/s41467-021-25103-7 PMC8361097

[15] Butler A , Hoffman P , Smibert P , Papalexi E , Satija R . Integrating single‐cell transcriptomic data across different conditions, technologies, and species. Nat Biotechnol. 2018;36(5):411‐420. 29608179 10.1038/nbt.4096 PMC6700744

[16] McInnes L , Healy J , Saul N , Grossberger L . UMAP: uniform manifold approximation and projection. J Open Source Software. 2018;3(1):861.

[17] Pei G , Yan F , Simon LM , Dai Y , Jia P , Zhao Z . deCS: a tool for systematic cell type annotations of single‐cell RNA sequencing data among human tissues. Biorxiv. 2021. 2021.09.19.460993.10.1016/j.gpb.2022.04.00135470070

[18] Aran D , Looney AP , Liu L , et al. Reference‐based analysis of lung single‐cell sequencing reveals a transitional profibrotic macrophage. Nat Immunol. 2019;20(2):163‐172. 30643263 10.1038/s41590-018-0276-y PMC6340744

[19] Han X , Zhou Z , Fei L , et al. Construction of a human cell landscape at single‐cell level. Nature. 2020;581(7808):303‐309. 32214235 10.1038/s41586-020-2157-4

[20] Monaco G , Lee B , Xu W , et al. RNA‐seq signatures normalized by mRNA abundance allow absolute deconvolution of human immune cell types. Cell Rep. 2019;26(6):1627‐1640.e7. 30726743 10.1016/j.celrep.2019.01.041 PMC6367568

[21] Conway JR , Lex A , Gehlenborg N . UpSetR: an R package for the visualization of intersecting sets and their properties. Bioinformatics. 2017;33(18):2938‐2940. 28645171 10.1093/bioinformatics/btx364 PMC5870712

[22] Liao Y , Wang J , Jaehnig EJ , Shi Z , Zhang B . WebGestalt 2019: gene set analysis toolkit with revamped UIs and APIs. Nucleic Acids Res. 2019;47(W1):W199‐W205. 31114916 10.1093/nar/gkz401 PMC6602449

[23] Benjamini Y , Hochberg Y . Controlling the false discovery rate ‐ a practical and powerful approach to multiple testing. J R Stat Soc B. 1995;57:289‐300.

[24] Jin S , Guerrero‐Juarez CF , Zhang L , et al. Inference and analysis of cell–cell communication using CellChat. Nat Commun. 2021;12(1):1088. 33597522 10.1038/s41467-021-21246-9 PMC7889871

[25] Kim SY , Volsky DJ . PAGE: parametric analysis of gene set enrichment. BMC Bioinf. 2005;6:144. 10.1186/1471-2105-6-144 PMC1183189 15941488

[26] Dries R , Zhu Q , Dong R , et al. Giotto: a toolbox for integrative analysis and visualization of spatial expression data. Genome Biol. 2021;22(1):78. 33685491 10.1186/s13059-021-02286-2 PMC7938609

[27] Elosua‐Bayes M , Nieto P , Mereu E , Gut I , Heyn H . SPOTlight: seeded NMF regression to deconvolute spatial transcriptomics spots with single‐cell transcriptomes. Nucleic Acids Res. 2021;49(9):e50. 33544846 10.1093/nar/gkab043 PMC8136778

[28] Zhao E , Stone MR , Ren X , et al. Spatial transcriptomics at subspot resolution with BayesSpace. Nat Biotechnol. 2021;39(11):1375‐1384. 34083791 10.1038/s41587-021-00935-2 PMC8763026

[29] Browaeys R , Saelens W , Saeys Y . NicheNet: modeling intercellular communication by linking ligands to target genes. Nat Methods. 2020;17(2):159‐162. 31819264 10.1038/s41592-019-0667-5

[30] Wei R , He S , Bai S , et al. Spatial charting of single‐cell transcriptomes in tissues. Nat Biotechnol. 2022;40(8):1190‐1199. 35314812 10.1038/s41587-022-01233-1 PMC9673606

[31] Shen D , Podolnikova NP , Yakubenko VP , et al. Pleiotrophin, a multifunctional cytokine and growth factor, induces leukocyte responses through the integrin Mac‐1. J Biol Chem. 2017;292(46):18848‐18861. 28939773 10.1074/jbc.M116.773713 PMC5704470

[32] Stuart T , Butler A , Hoffman P , et al. Comprehensive integration of single‐cell data. Cell. 2019;177(7):1888‐1902.e21. 31178118 10.1016/j.cell.2019.05.031 PMC6687398

[33] Huang R , Gu W , Sun B , Gao L . Identification of COL4A1 as a potential gene conferring trastuzumab resistance in gastric cancer based on bioinformatics analysis. Mol Med Rep. 2018;17(5):6387‐6396. 29512712 10.3892/mmr.2018.8664 PMC5928613

[34] Wang T , Jin H , Hu J , et al. COL4A1 promotes the growth and metastasis of hepatocellular carcinoma cells by activating FAK‐Src signaling. J Exp Clin Cancer Res. 2020;39(1):1‐16. 32746865 10.1186/s13046-020-01650-7 PMC7398077

[35] Szarvas T , Reis H , Kramer G , et al. Enhanced stromal syndecan‐1 expression is an independent risk factor for poor survival in bladder cancer. Hum Pathol. 2014;45(4):674‐682. 24656090 10.1016/j.humpath.2013.10.036

[36] McGranahan N , Swanton C . Clonal heterogeneity and tumor evolution: past, present, and the future. Cell. 2017;168(4):613‐628. 28187284 10.1016/j.cell.2017.01.018

[37] Lindskrog SV , Prip F , Lamy P , et al. An integrated multi‐omics analysis identifies prognostic molecular subtypes of non‐muscle‐invasive bladder cancer. Nat Commun. 2021;12(1):2301. 33863885 10.1038/s41467-021-22465-w PMC8052448

[38] Gambardella G , Viscido G , Tumaini B , Isacchi A , Bosotti R , di Bernardo D . A single‐cell analysis of breast cancer cell lines to study tumour heterogeneity and drug response. Nat Commun. 2022;13(1):1714. 35361816 10.1038/s41467-022-29358-6 PMC8971486

[39] Peng WS , Zhou X , Yan WB , et al. Dissecting the heterogeneity of the microenvironment in primary and recurrent nasopharyngeal carcinomas using single‐cell RNA sequencing. Oncoimmunology. 2022;11(1):2026583. 35096485 10.1080/2162402X.2022.2026583 PMC8794254

[40] Sun Y , Wu L , Zhong Y , et al. Single‐cell landscape of the ecosystem in early‐relapse hepatocellular carcinoma. Cell. 2021;184(2):404‐421.e16. 33357445 10.1016/j.cell.2020.11.041

[41] Amsen D , van Gisbergen K , Hombrink P , van Lier RAW . Tissue‐resident memory T cells at the center of immunity to solid tumors. Nat Immunol. 2018;19(6):538‐546. 29777219 10.1038/s41590-018-0114-2

[42] Kato T , Noma K , Ohara T , et al. Cancer‐associated fibroblasts affect intratumoral CD8(+) and FoxP3(+) T cells via IL6 in the tumor microenvironment. Clin Cancer Res. 2018;24(19):4820‐4833. 29921731 10.1158/1078-0432.CCR-18-0205

[43] Liu T , Han C , Wang S , et al. Cancer‐associated fibroblasts: an emerging target of anti‐cancer immunotherapy. J Hematol Oncol. 2019;12(1):86. 31462327 10.1186/s13045-019-0770-1 PMC6714445

[44] Zhang M , Yang H , Wan L , et al. Single‐cell transcriptomic architecture and intercellular crosstalk of human intrahepatic cholangiocarcinoma. J Hepatol. 2020;73(5):1118‐1130. 32505533 10.1016/j.jhep.2020.05.039

[45] Chen X , Song E . Turning foes to friends: targeting cancer‐associated fibroblasts. Nat Rev Drug Discov. 2019;18(2):99‐115. 30470818 10.1038/s41573-018-0004-1

[46] Galbo PM Jr , Zang X , Zheng D . Molecular features of cancer‐associated fibroblast subtypes and their implication on cancer pathogenesis, prognosis, and immunotherapy resistance. Clin Cancer Res. 2021;27(9):2636‐2647. 33622705 10.1158/1078-0432.CCR-20-4226 PMC8102353

[47] Peng YL , Xiong LB , Zhou ZH , et al. Single‐cell transcriptomics reveals a low CD8(+) T cell infiltrating state mediated by fibroblasts in recurrent renal cell carcinoma. J Immunother Cancer. 2022;10(2):e004206. 10.1136/jitc-2021-004206 PMC8819783 35121646

[48] Ohlund D , Handly‐Santana A , Biffi G , et al. Distinct populations of inflammatory fibroblasts and myofibroblasts in pancreatic cancer. J Exp Med. 2017;214(3):579‐596. 28232471 10.1084/jem.20162024 PMC5339682

[49] Murakami K , Kamat AM , Dai Y , et al. Application of a multiplex urinalysis test for the prediction of intravesical BCG treatment response: a pilot study. Cancer Biomark. 2022;33(1):151‐157. 34511488 10.3233/CBM-210221 PMC8925124

[50] Liu PF , Cao YW , Jiang HP , et al. Heterogeneity research in muscle‐invasive bladder cancer based on differential protein expression analysis. Med Oncol. 2014;31(9):21. 25085780 10.1007/s12032-014-0021-9

[51] Cao H , Cheng L , Yu J , Zhang Z , Luo Z , Chen D . Identifying the mRNAs associated with bladder cancer recurrence. Cancer Biomark. 2020;28(4):429‐437. 32390597 10.3233/CBM-190617 PMC12662371

[52] Hassan H , Greve B , Pavao MS , Kiesel L , Ibrahim SA , Gotte M . Syndecan‐1 modulates beta‐integrin‐dependent and interleukin‐6‐dependent functions in breast cancer cell adhesion, migration, and resistance to irradiation. FEBS J. 2013;280(10):2216‐2227. 23289672 10.1111/febs.12111

[53] Li X , Wang Q , Wu Z , Zheng J , Ji L . Integrated bioinformatics analysis for identification of the hub genes linked with prognosis of ovarian cancer patients. Comput Math Methods Med. 2022;2022:5113447. 35047055 10.1155/2022/5113447 PMC8763496

[54] Ryu D , Lee C . Expression quantitative trait loci for PI3K/AKT pathway. Medicine. 2017;96(1):e5817. 10.1097/MD.0000000000005817 PMC5228698 28072738

[55] Eke I , Cordes N . Focal adhesion signaling and therapy resistance in cancer. Semin Cancer Biol. 2015;31:65‐75. 25117005 10.1016/j.semcancer.2014.07.009

[56] Tong S , Yin H , Fu J , Li Y . Niban apoptosis regulator 1 promotes gemcitabine resistance by activating the focal adhesion kinase signaling pathway in bladder cancer. J Cancer. 2022;13(4):1103‐1118. 35281857 10.7150/jca.66248 PMC8899363

