# Supplementary figures and images for: Integrated single‐cell and spatial transcriptomic profiling reveals higher intratumour heterogeneity and epithelial–fibroblast interactions in recurrent bladder cancer
Source: Clin Transl Med. 2023 Jul 24;13(7):e1338. doi: 10.1002/ctm2.1338 (PMC10366350; doi:10.1002/ctm2.1338)

**BCSC-1**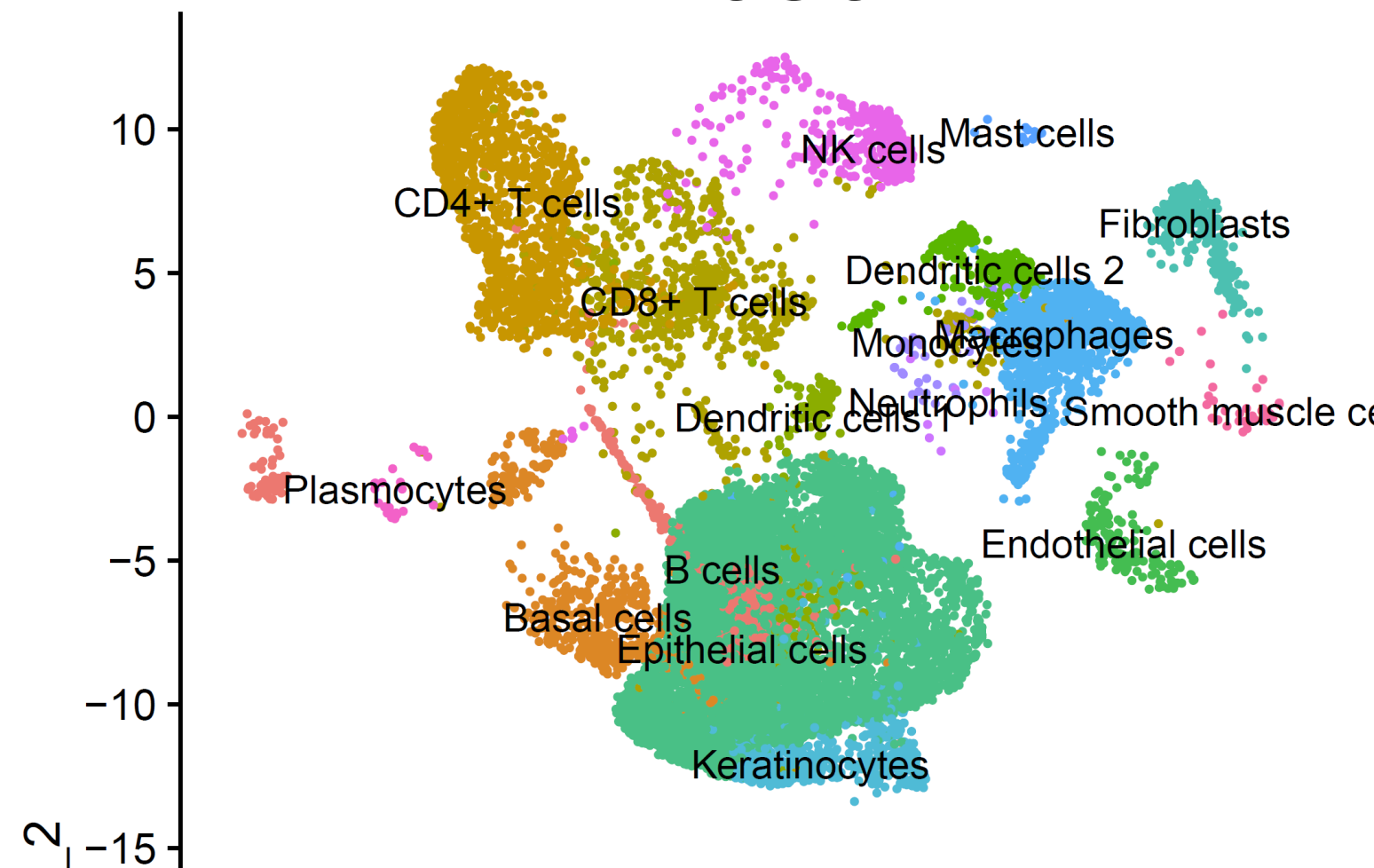**BCSC-2**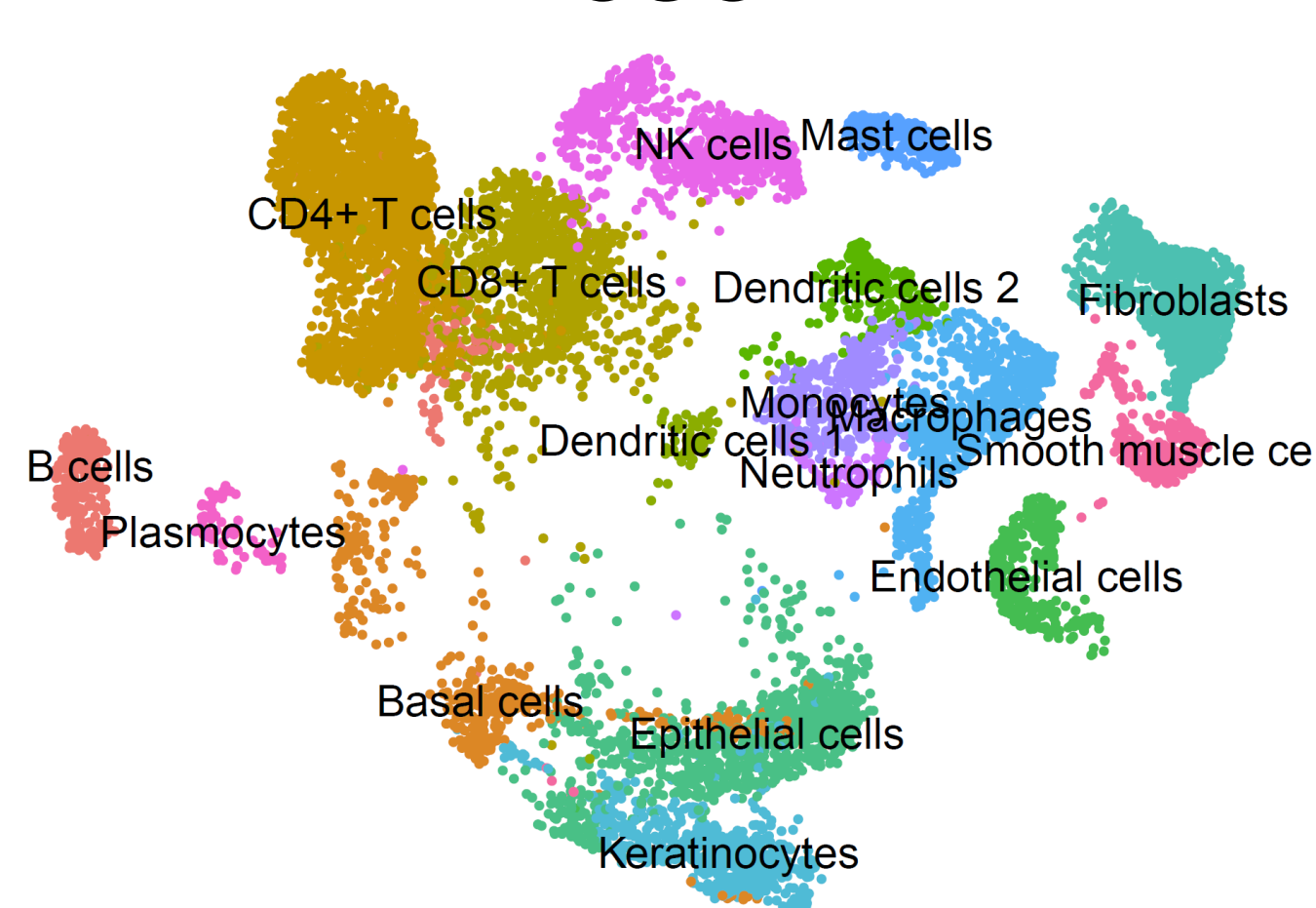**BCSC-3**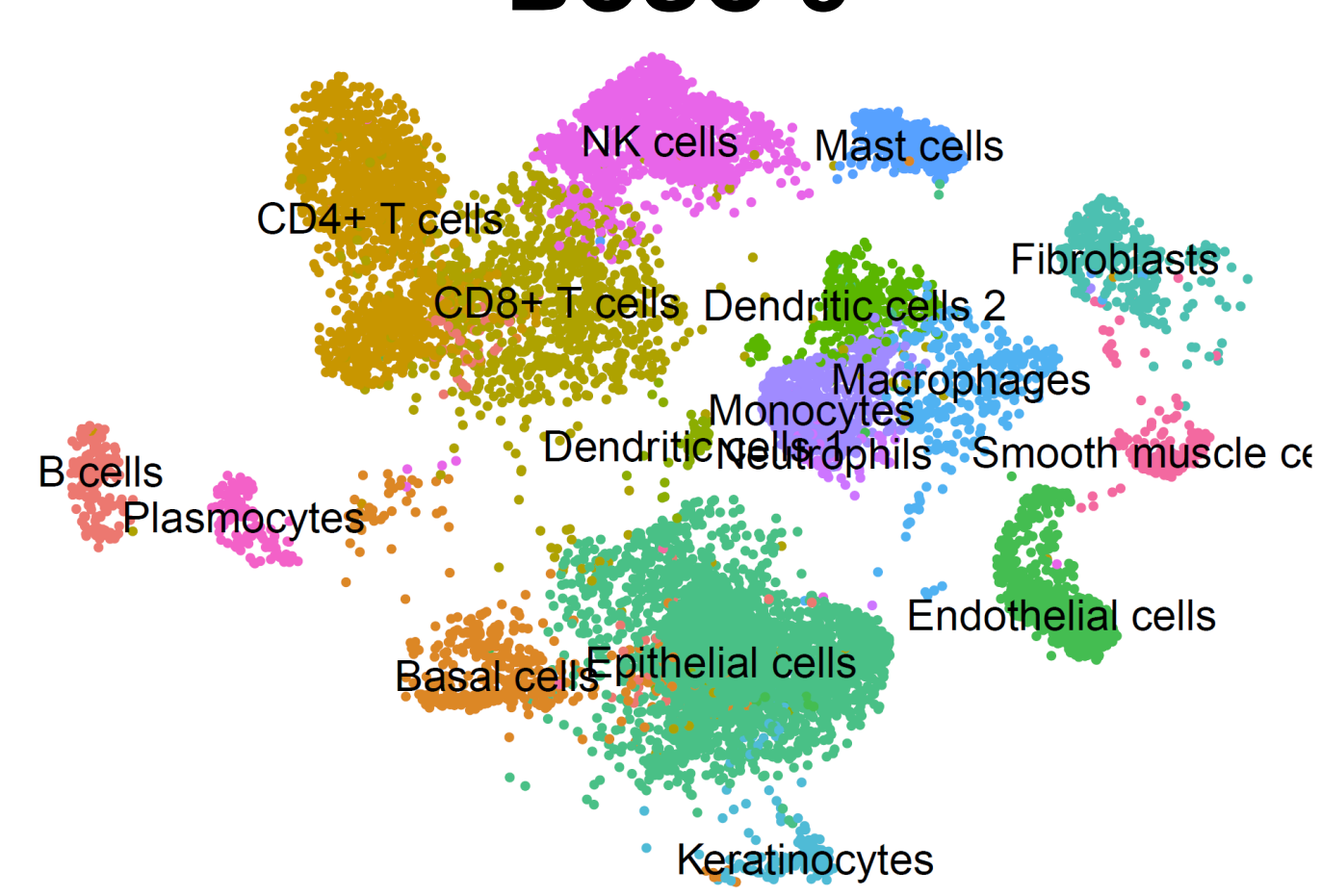**BCSC-4**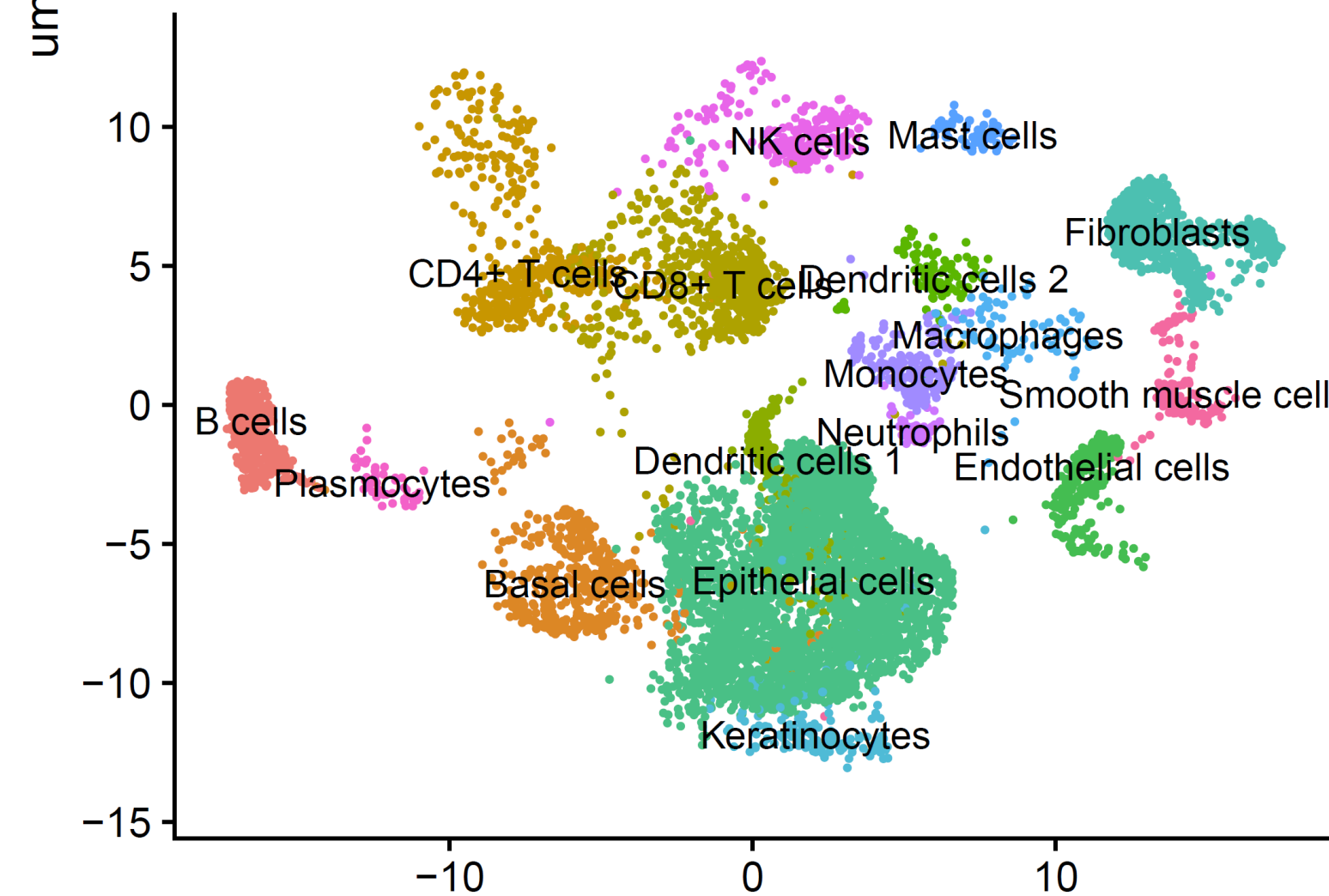**BCSC-5**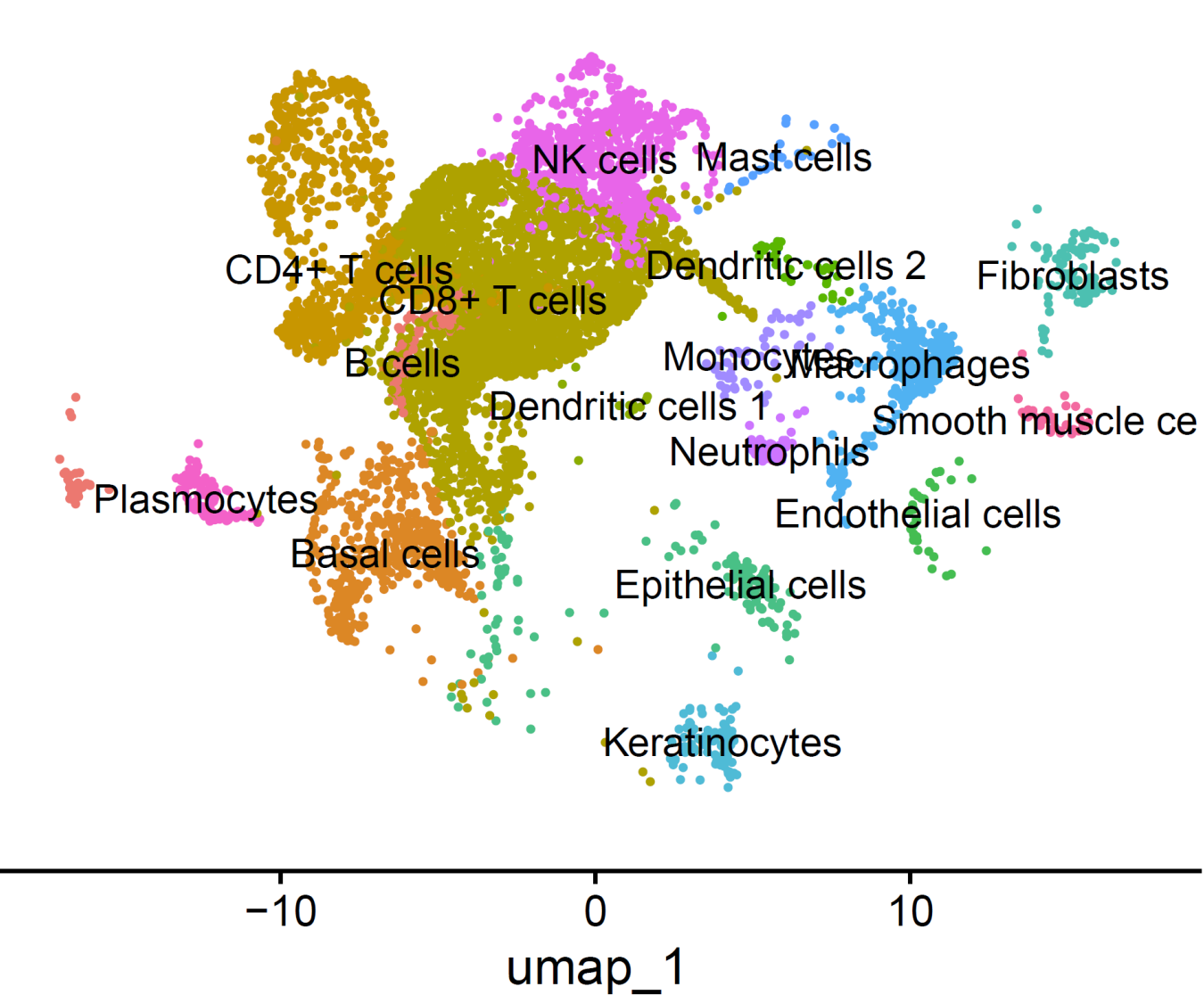**BCSC-6**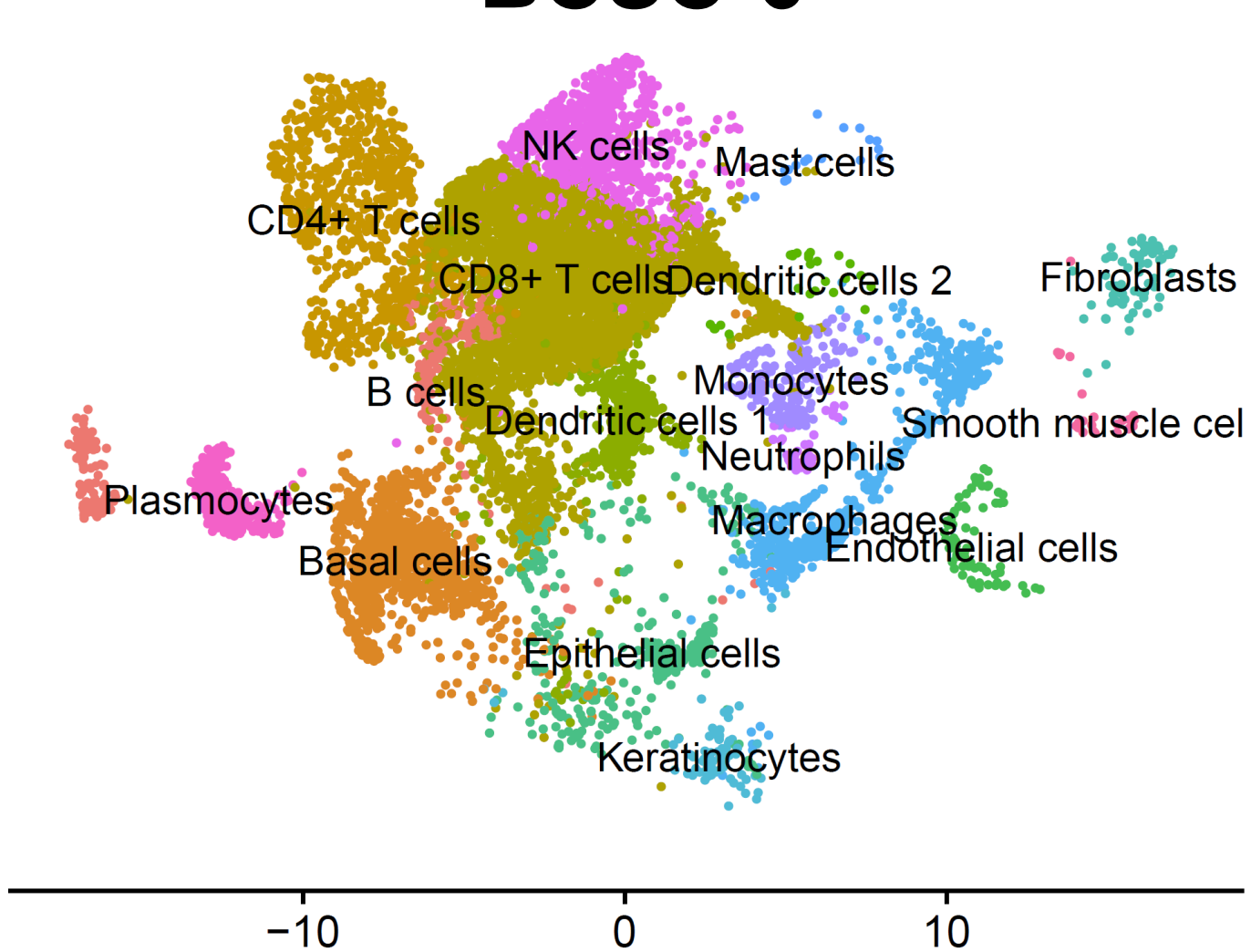

Supplement: Supplementary file 3 — Supporting Information [file CTM2-13-e1338-s003.pdf]

BCSC - 1

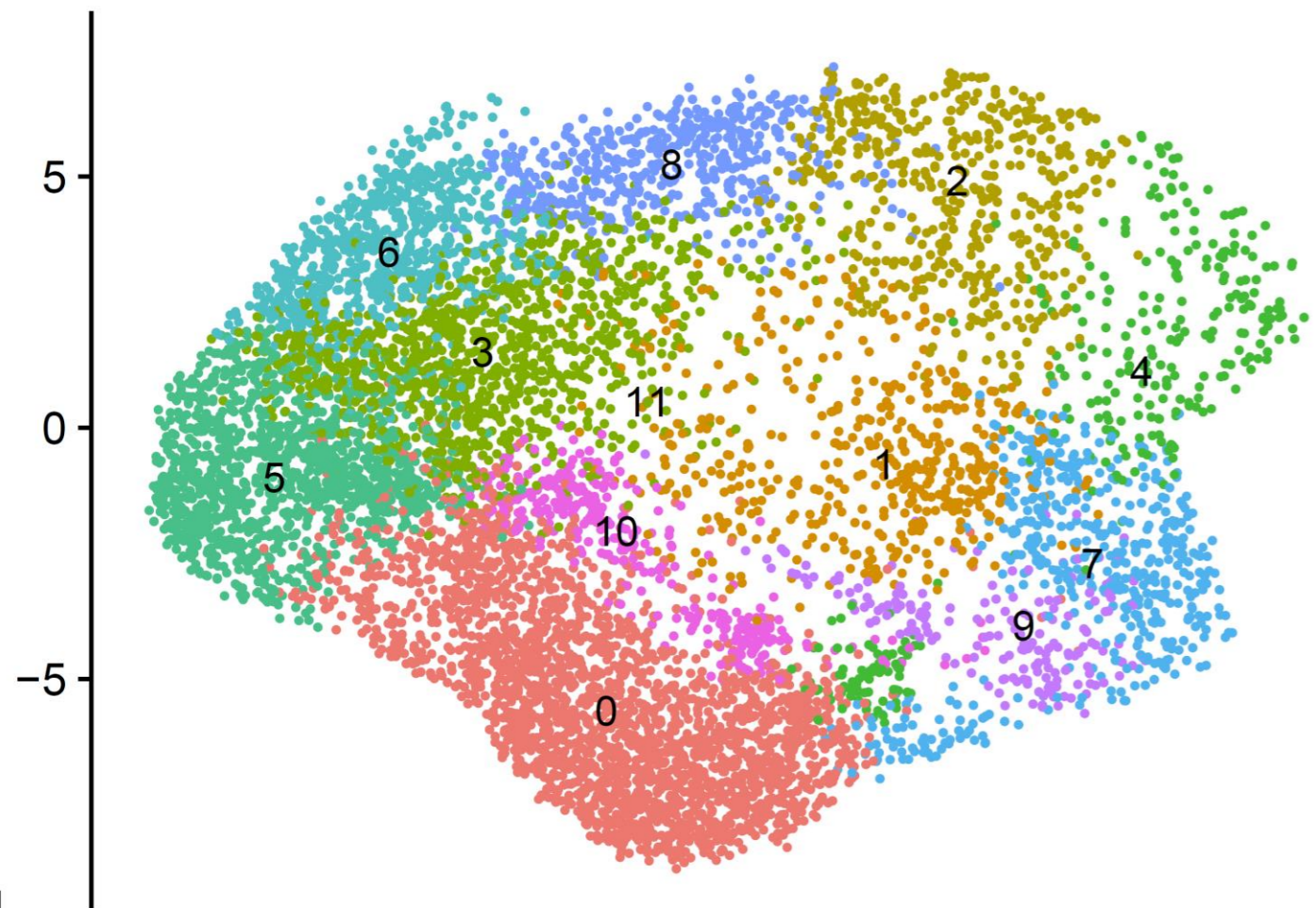

BCSC - 2

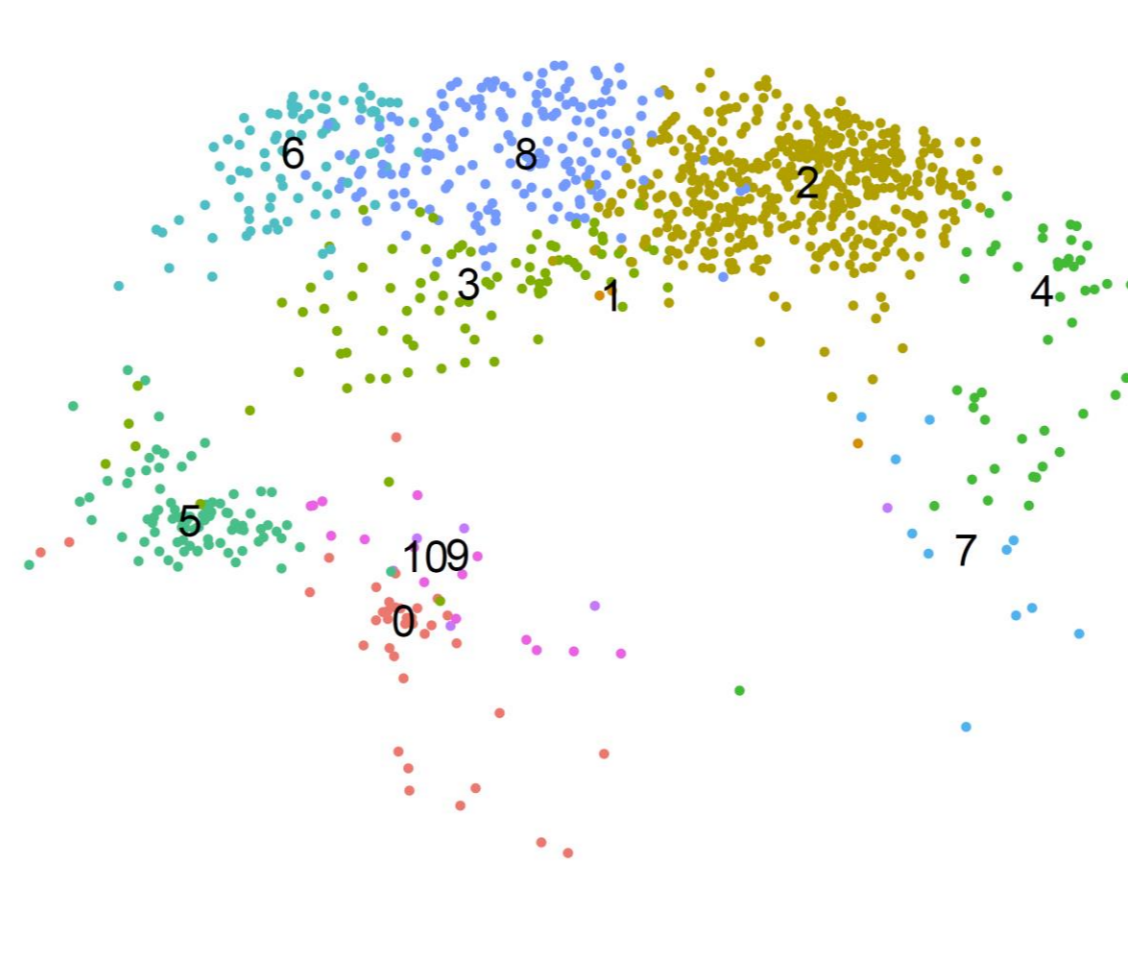

BCSC - 3

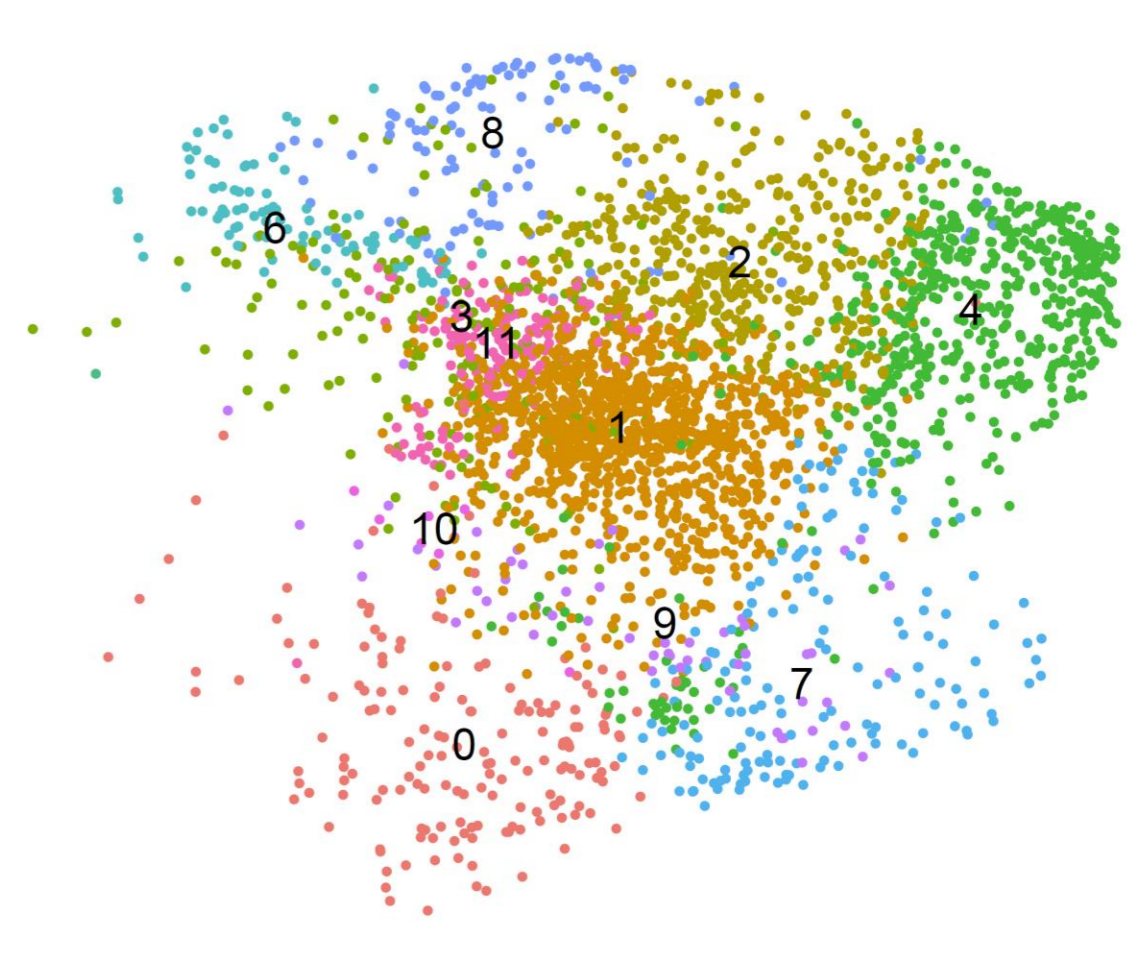

BCSC - 4

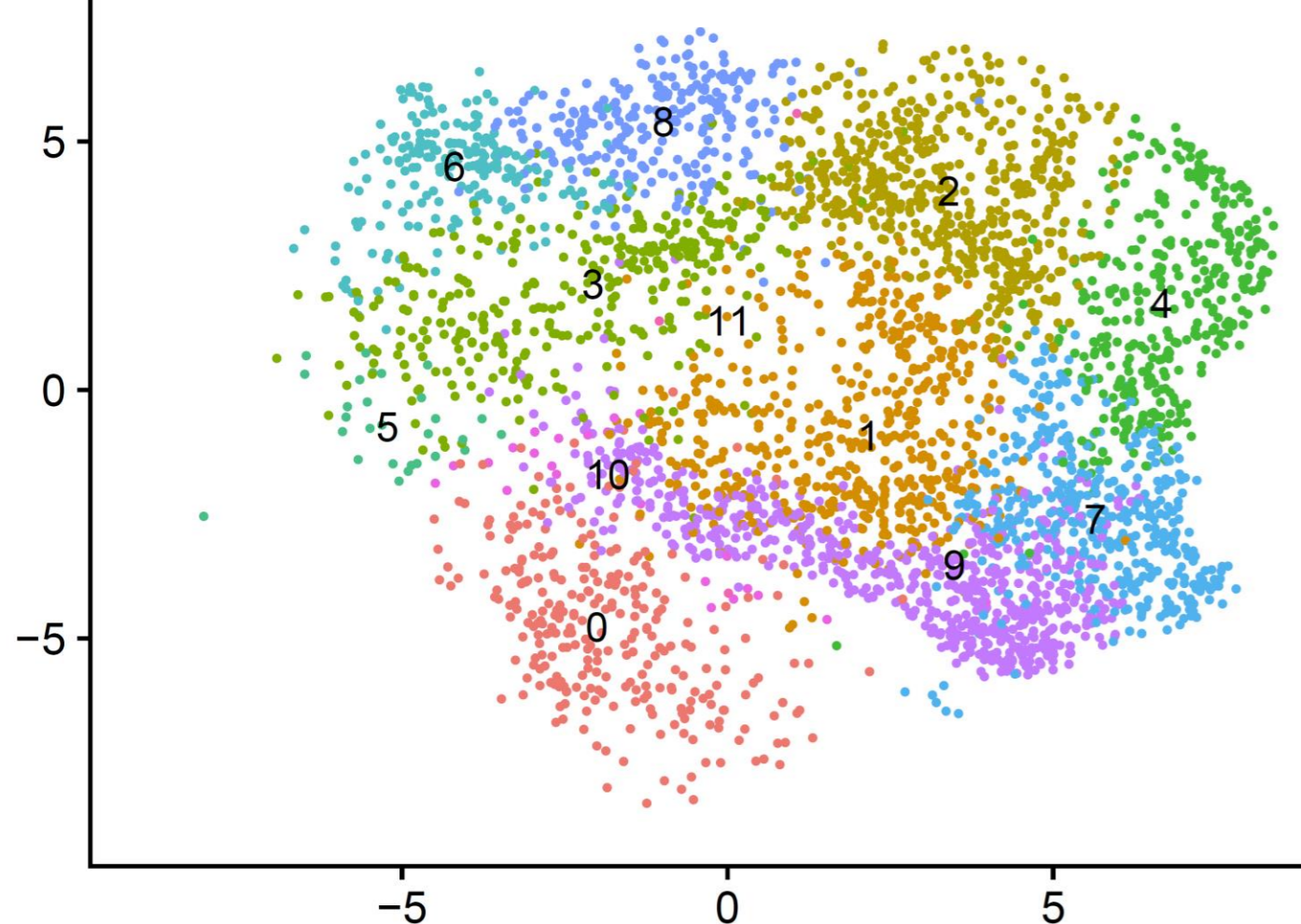

BCSC - 5

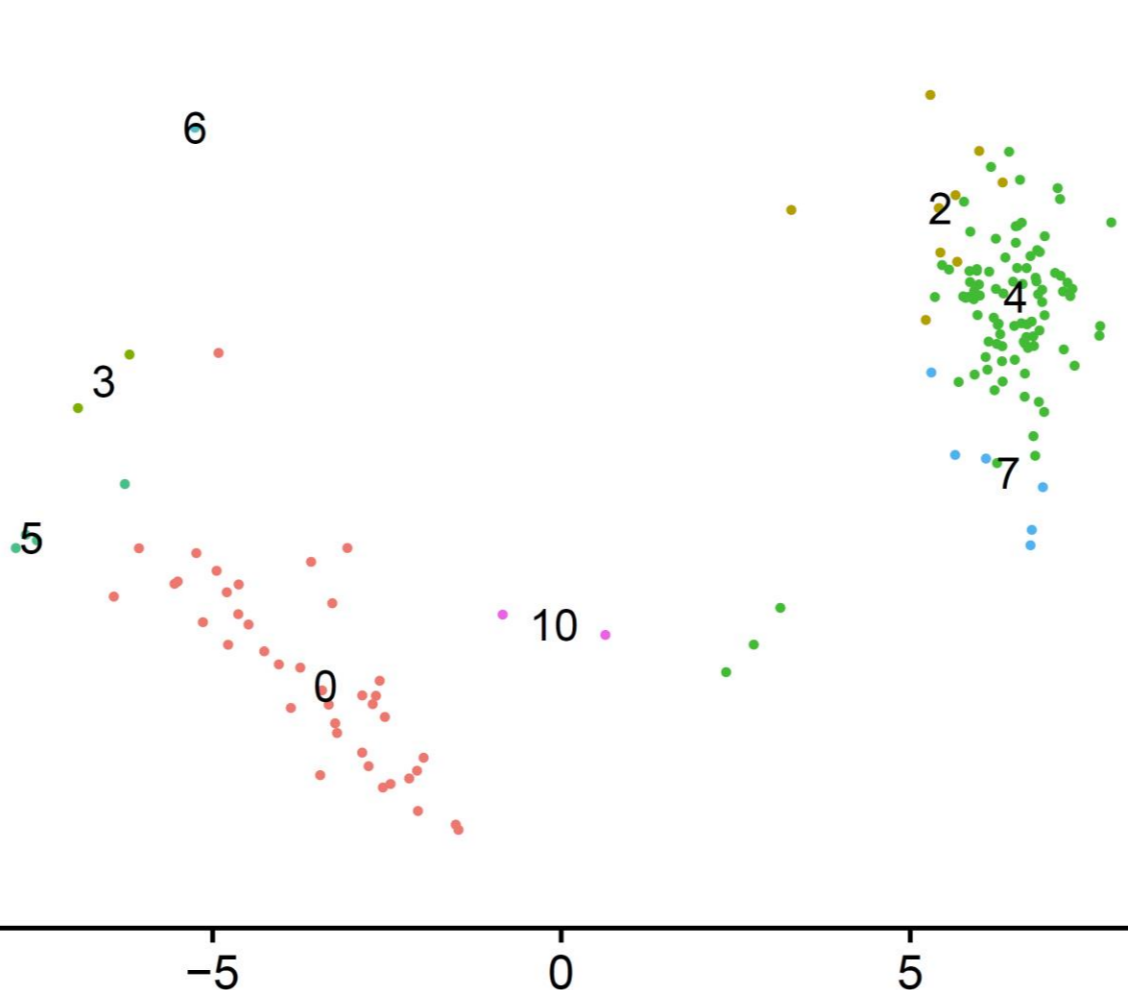

BCSC - 6

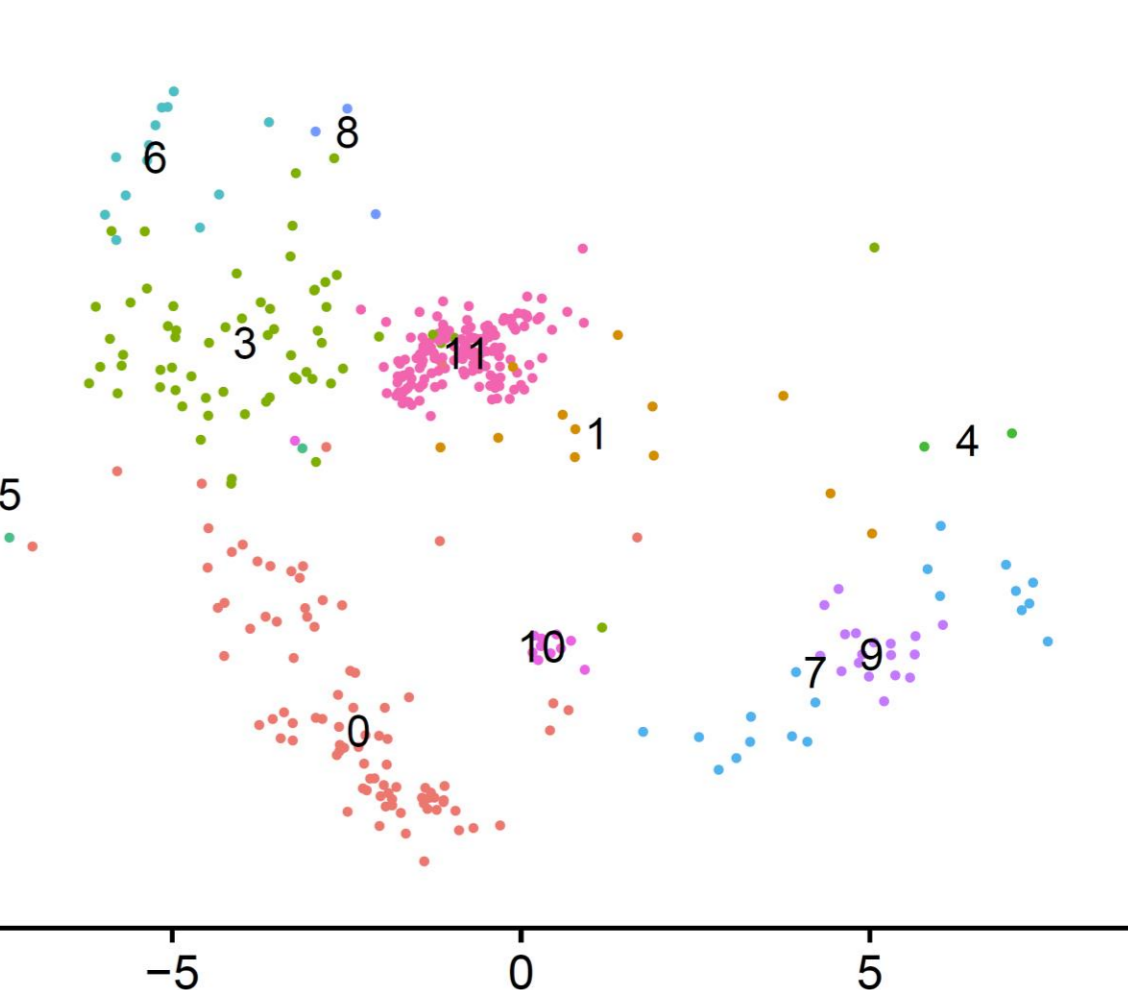

Supplement: Supplementary file 4 — Supporting Information [file CTM2-13-e1338-s005.pdf]

# A

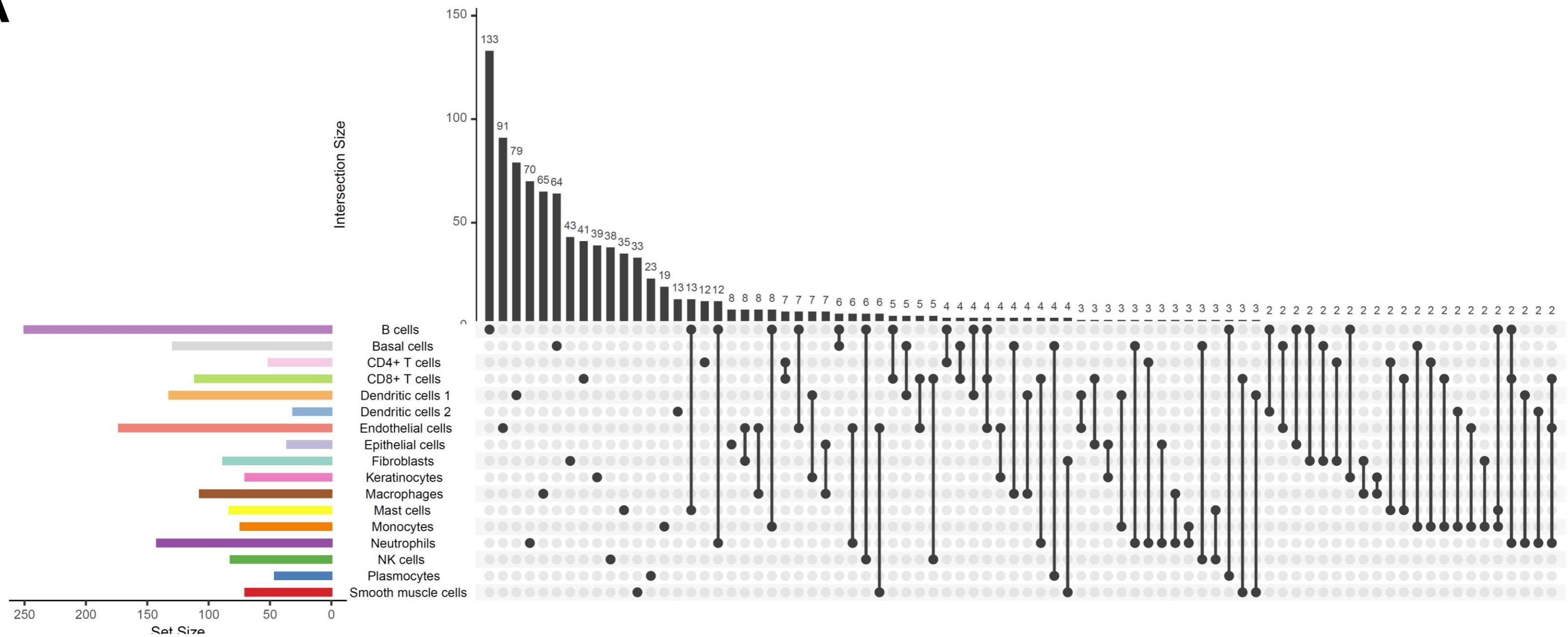

# B

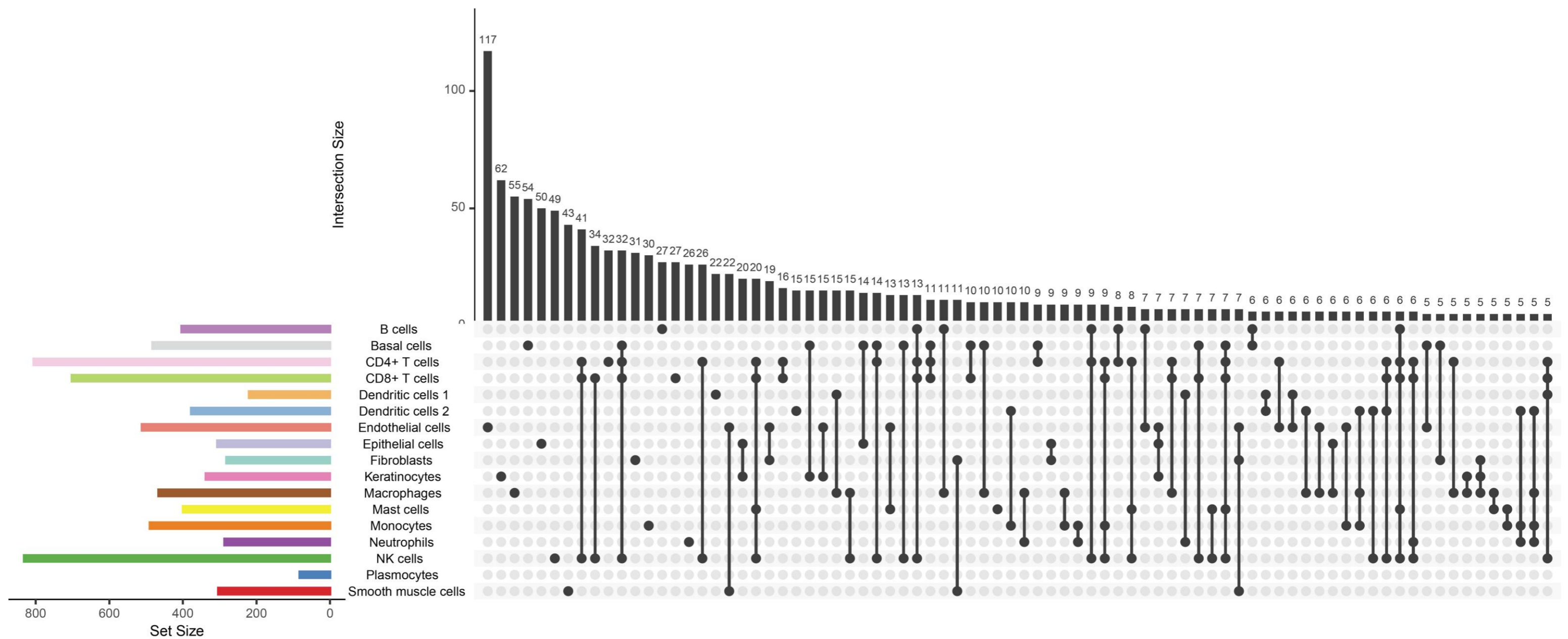

Supplement: Supplementary file 5 — Supporting Information [file CTM2-13-e1338-s010.pdf]

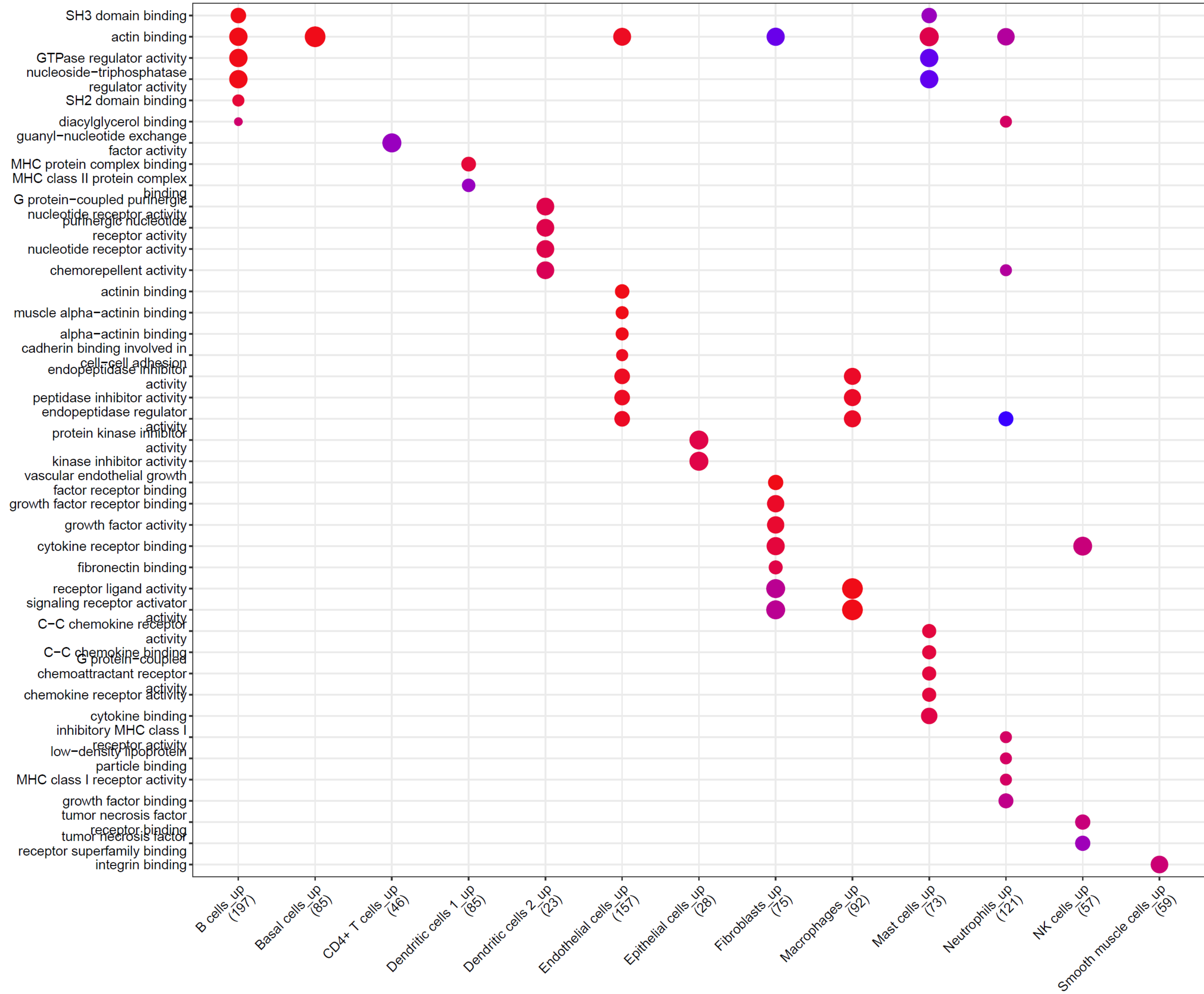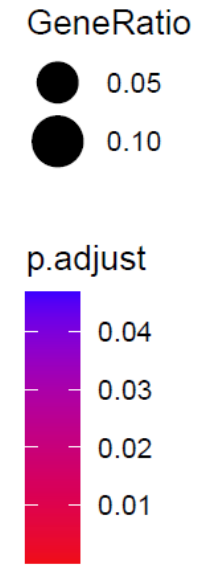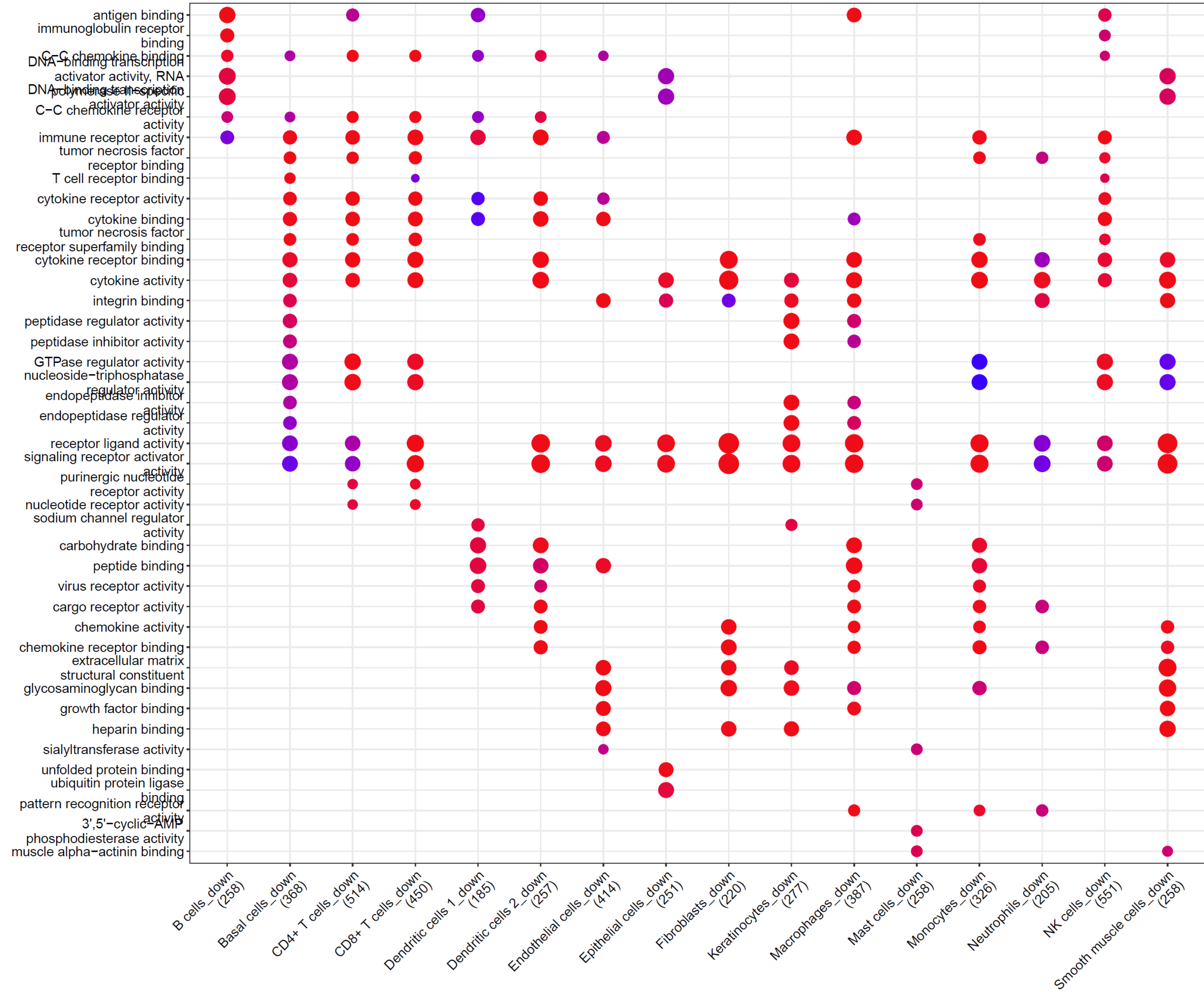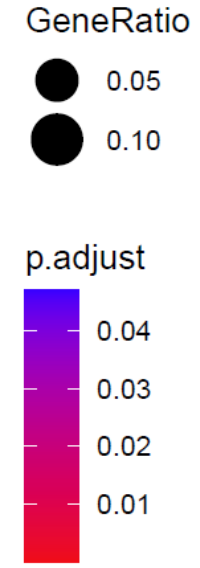

Supplement: Supplementary file 6 — Supporting Information [file CTM2-13-e1338-s001.pdf]

BCST-1

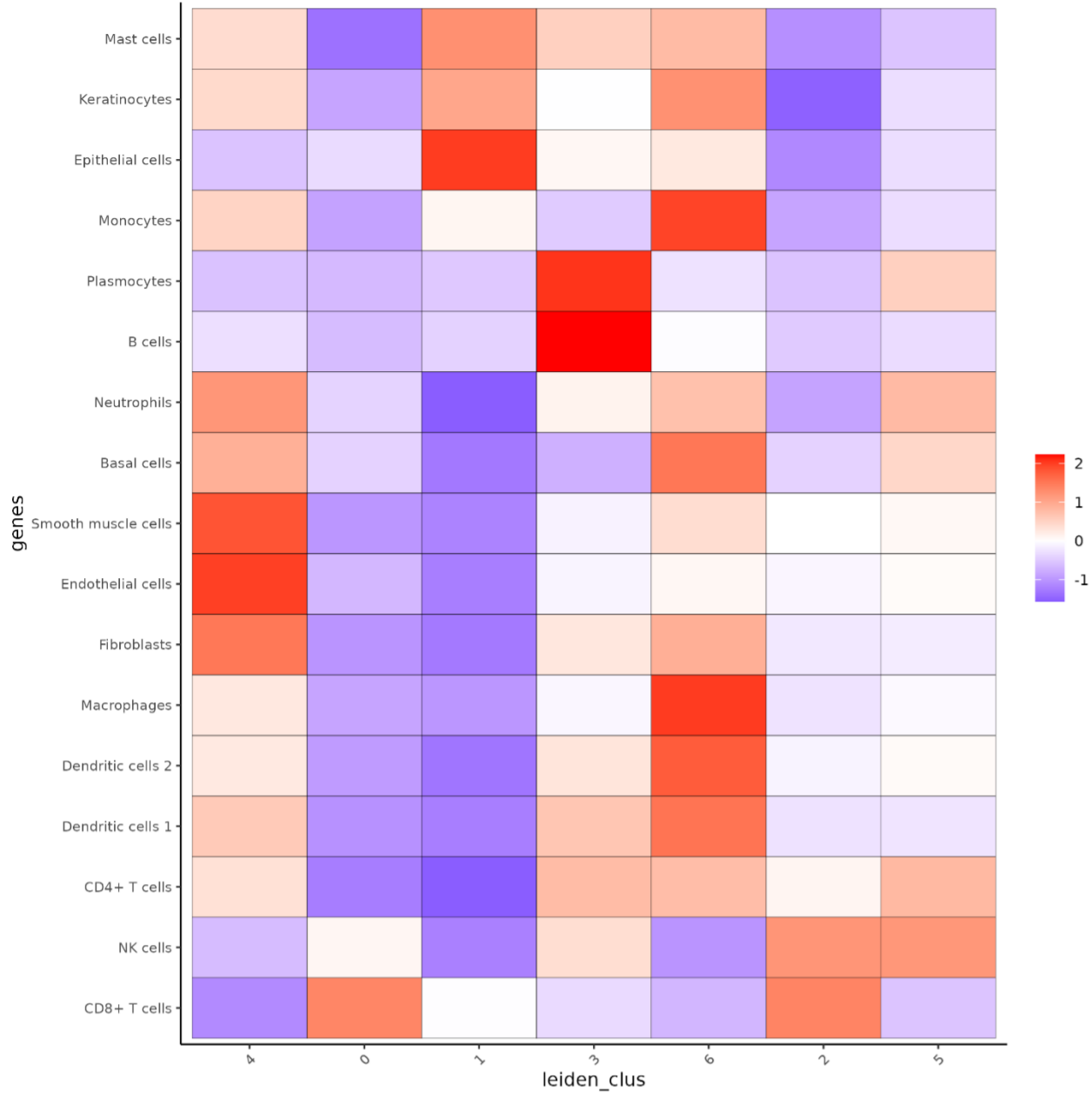

BCST-2

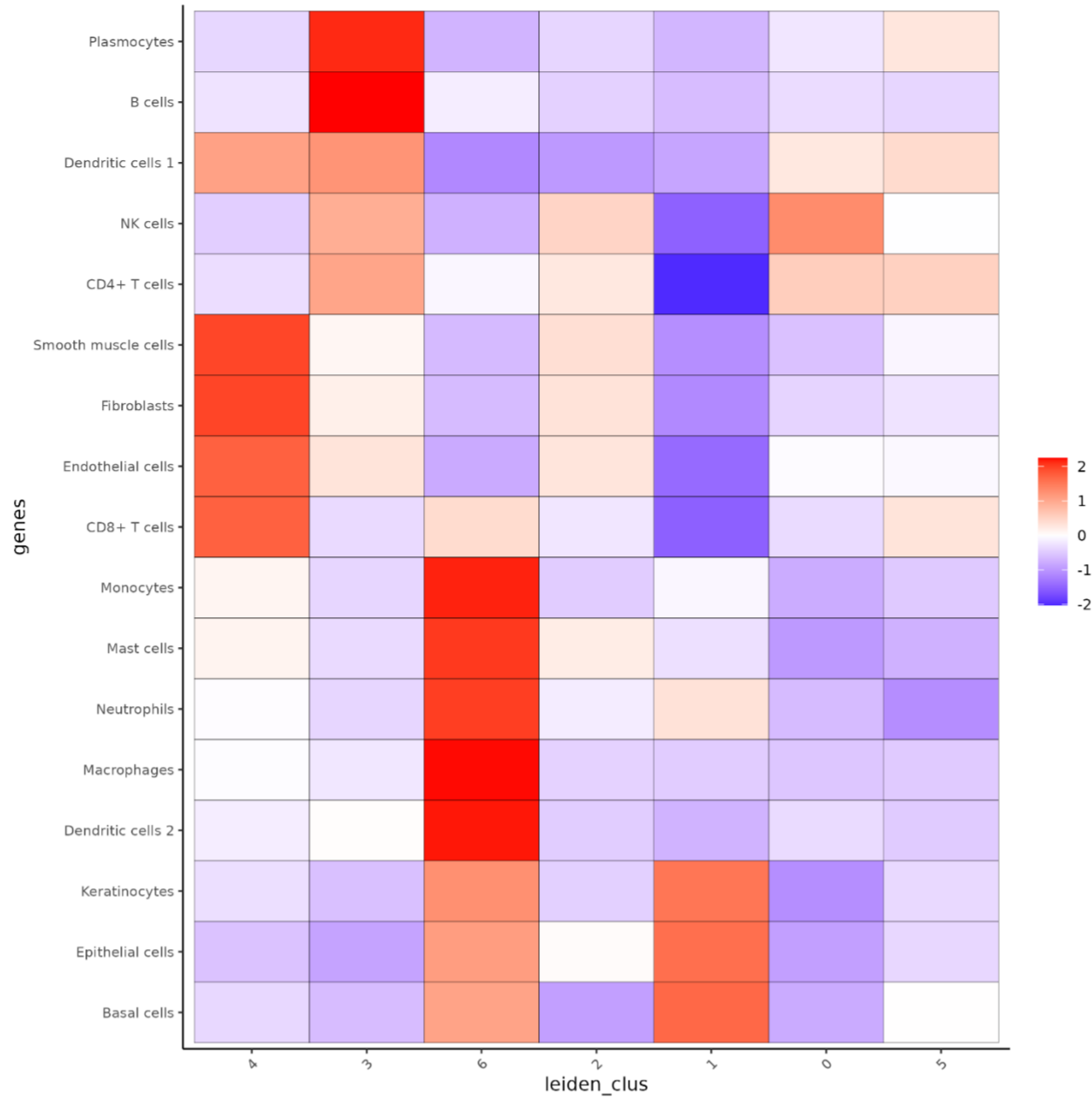

BCST-3

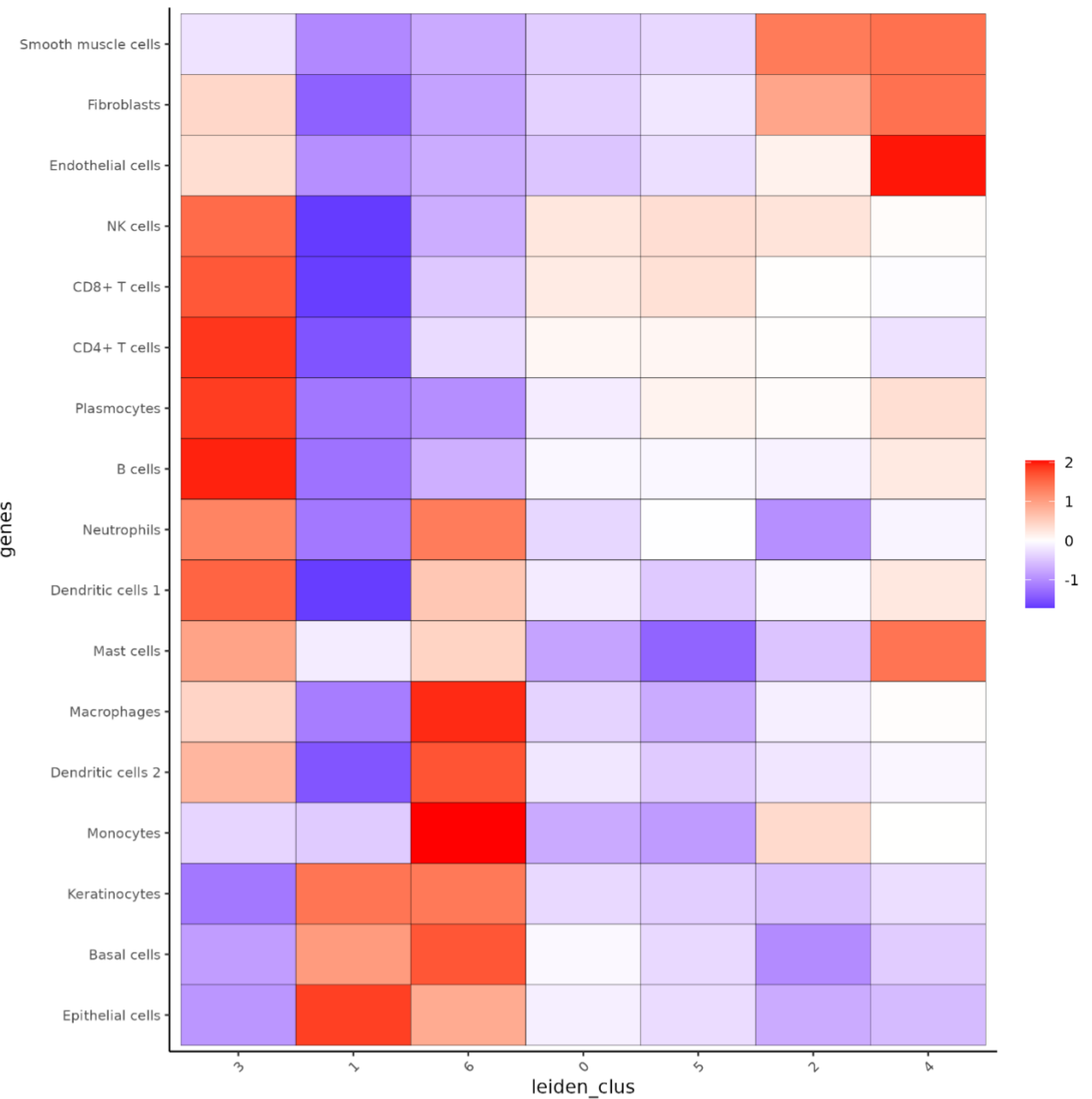

BCST-4

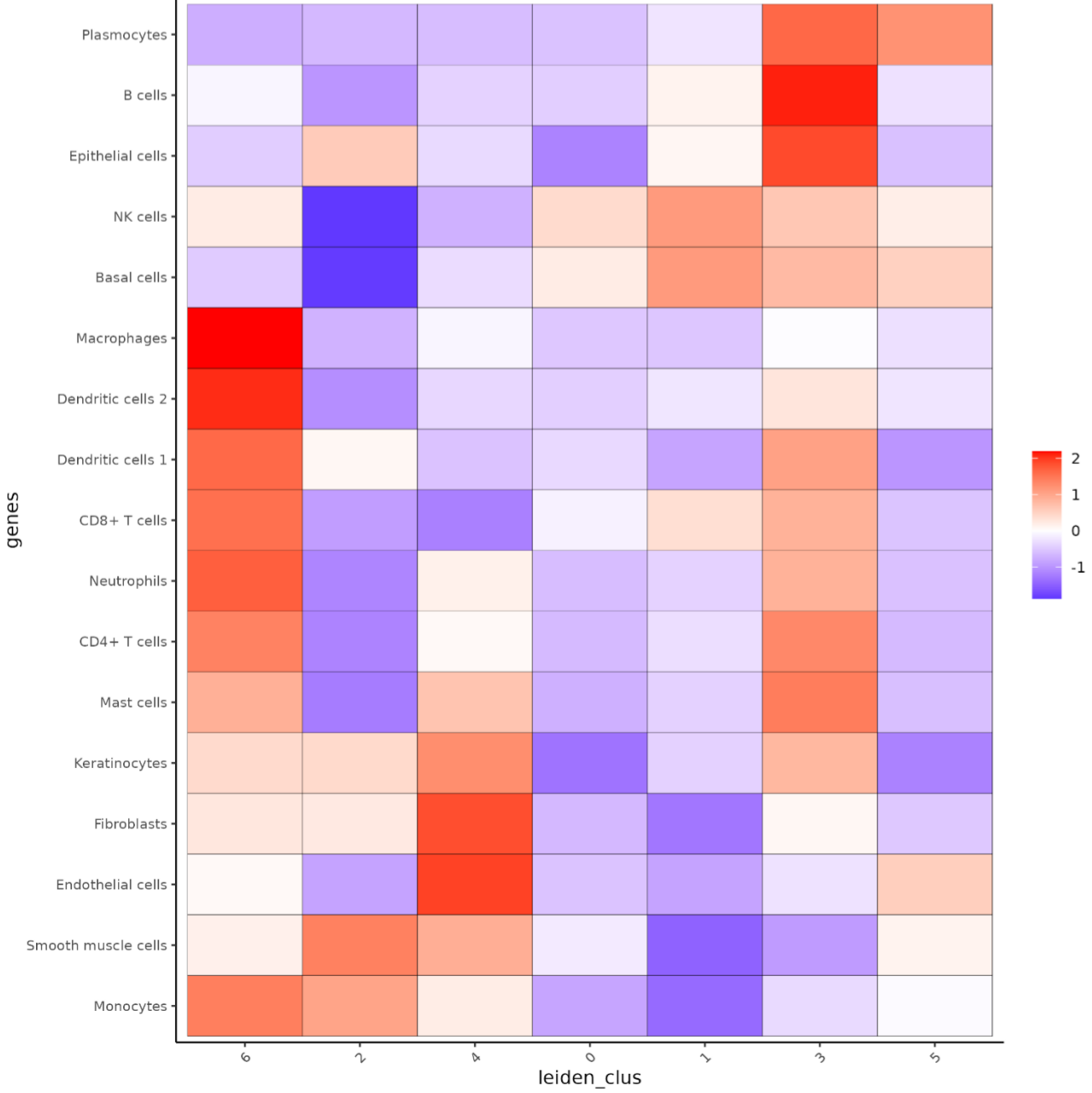

BCST-5

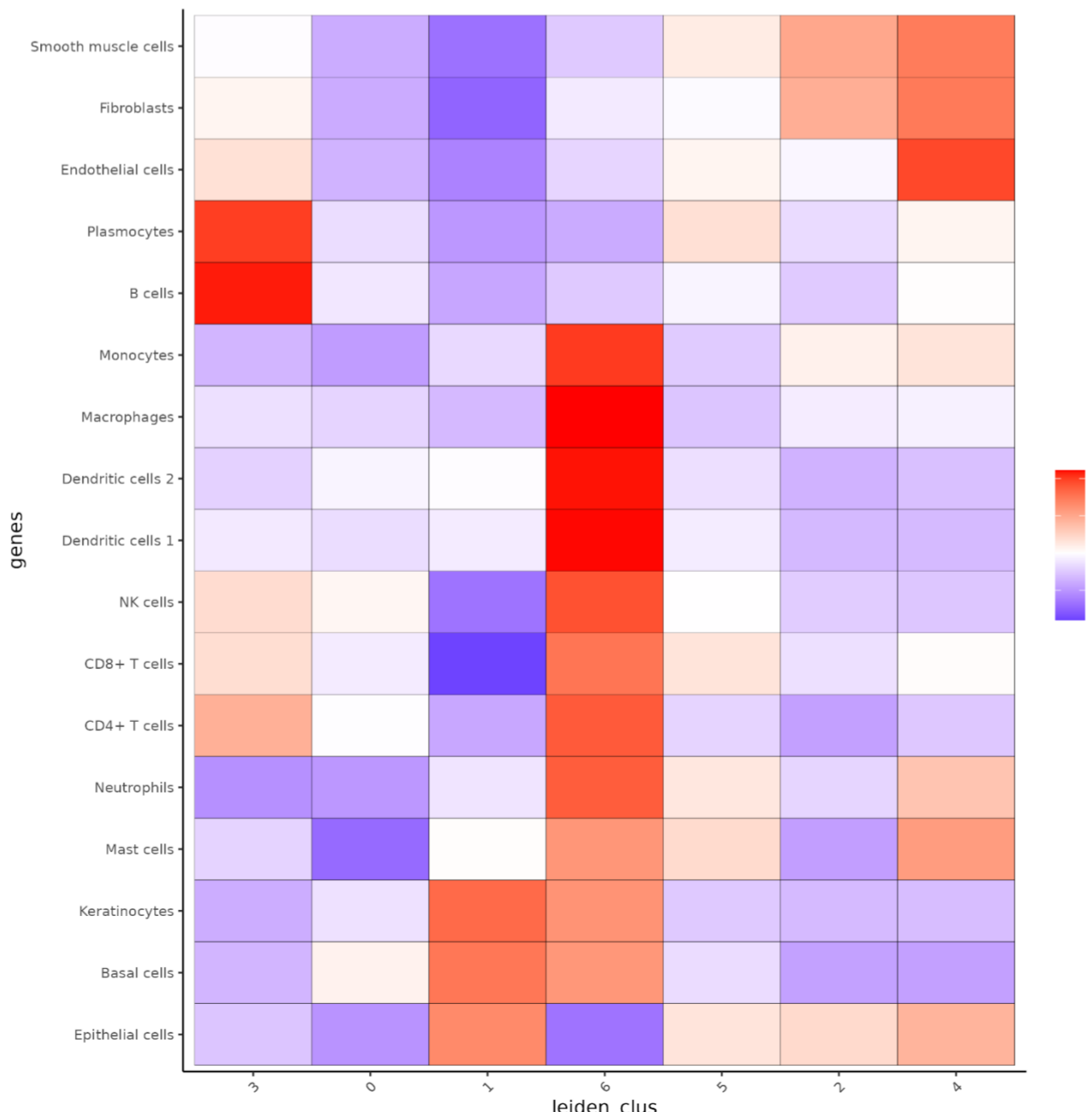

BCST-6

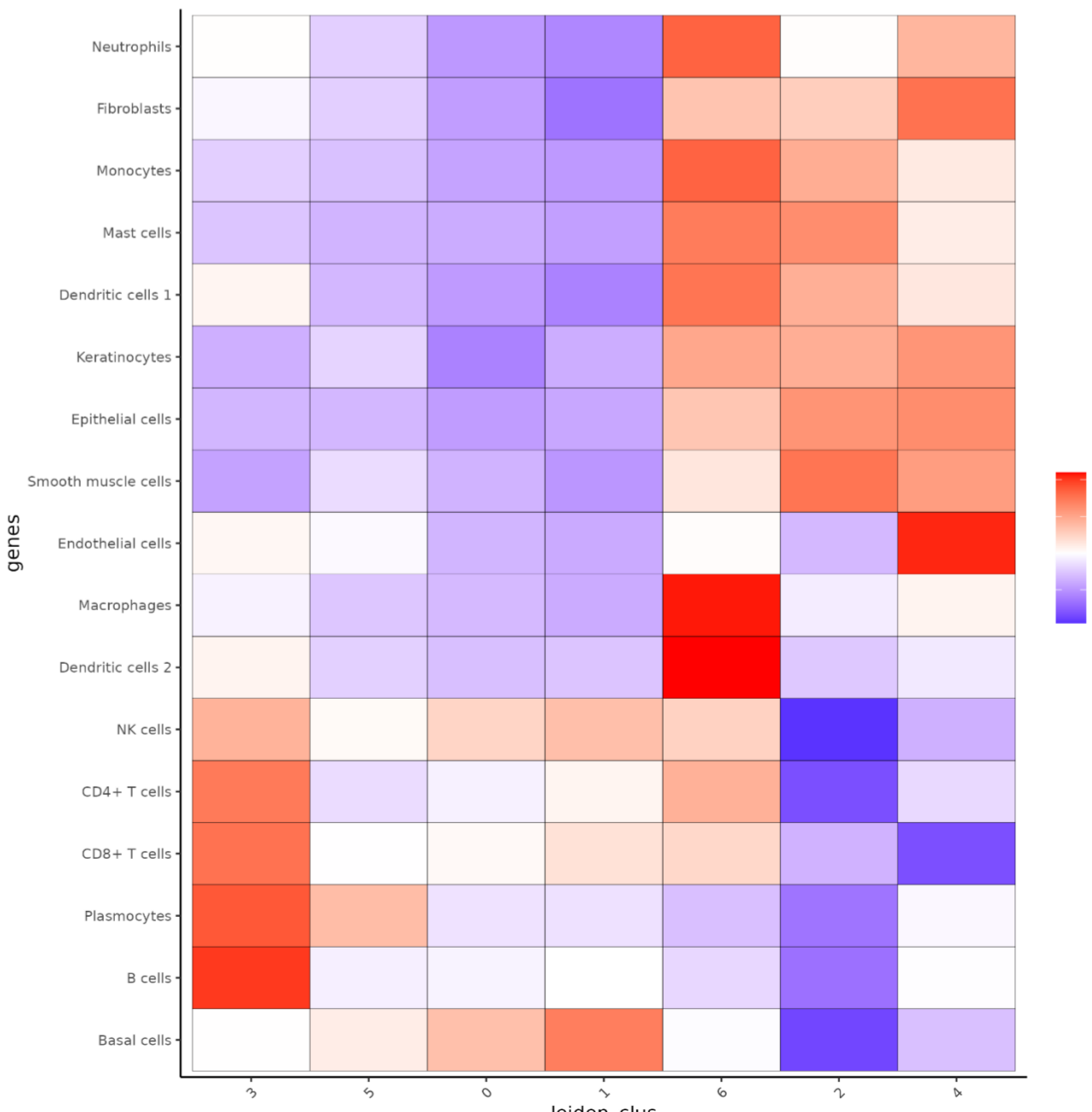

Supplement: Supplementary file 7 — Supporting Information [file CTM2-13-e1338-s007.pdf]

BCST-1

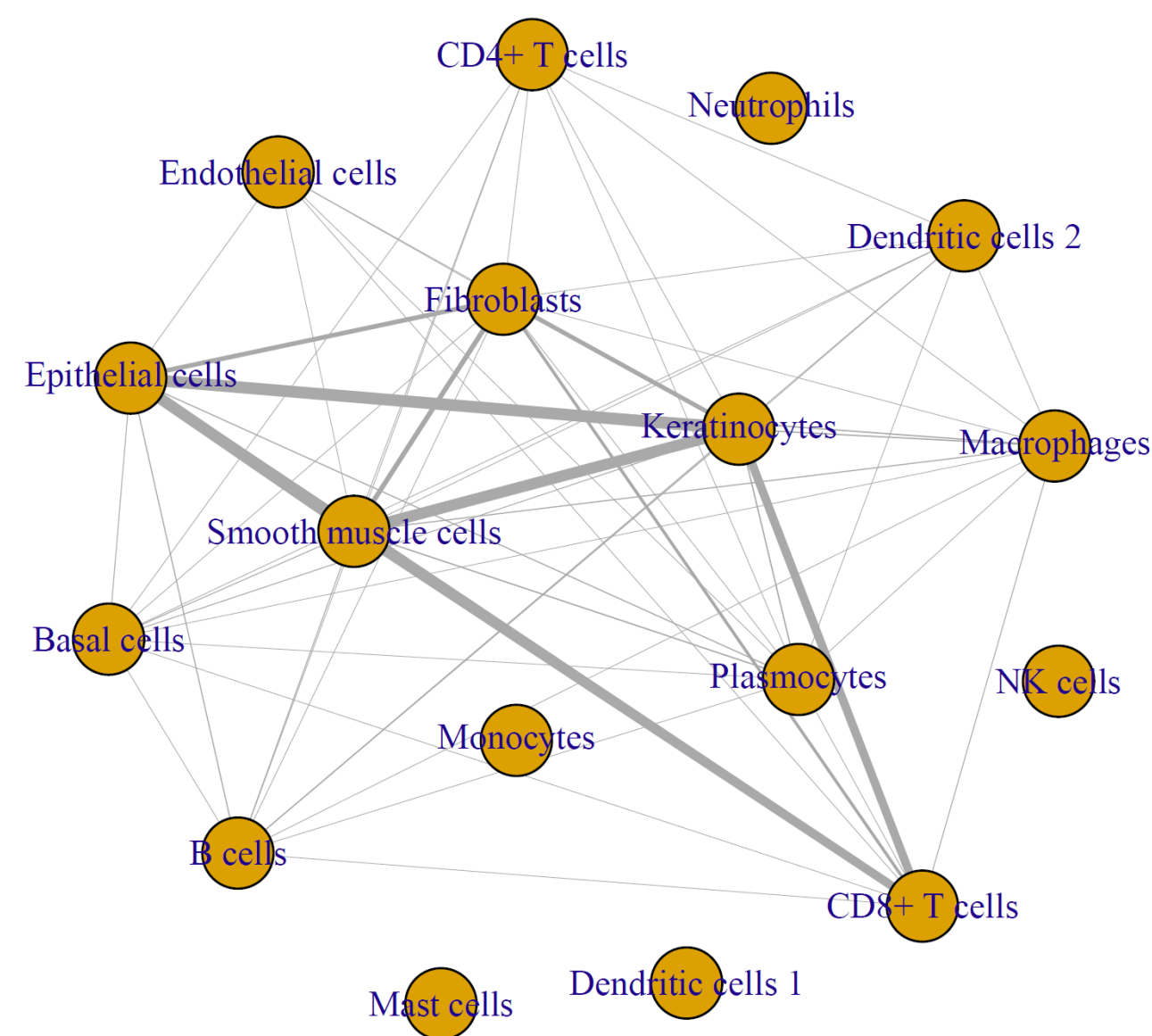

BCST-2

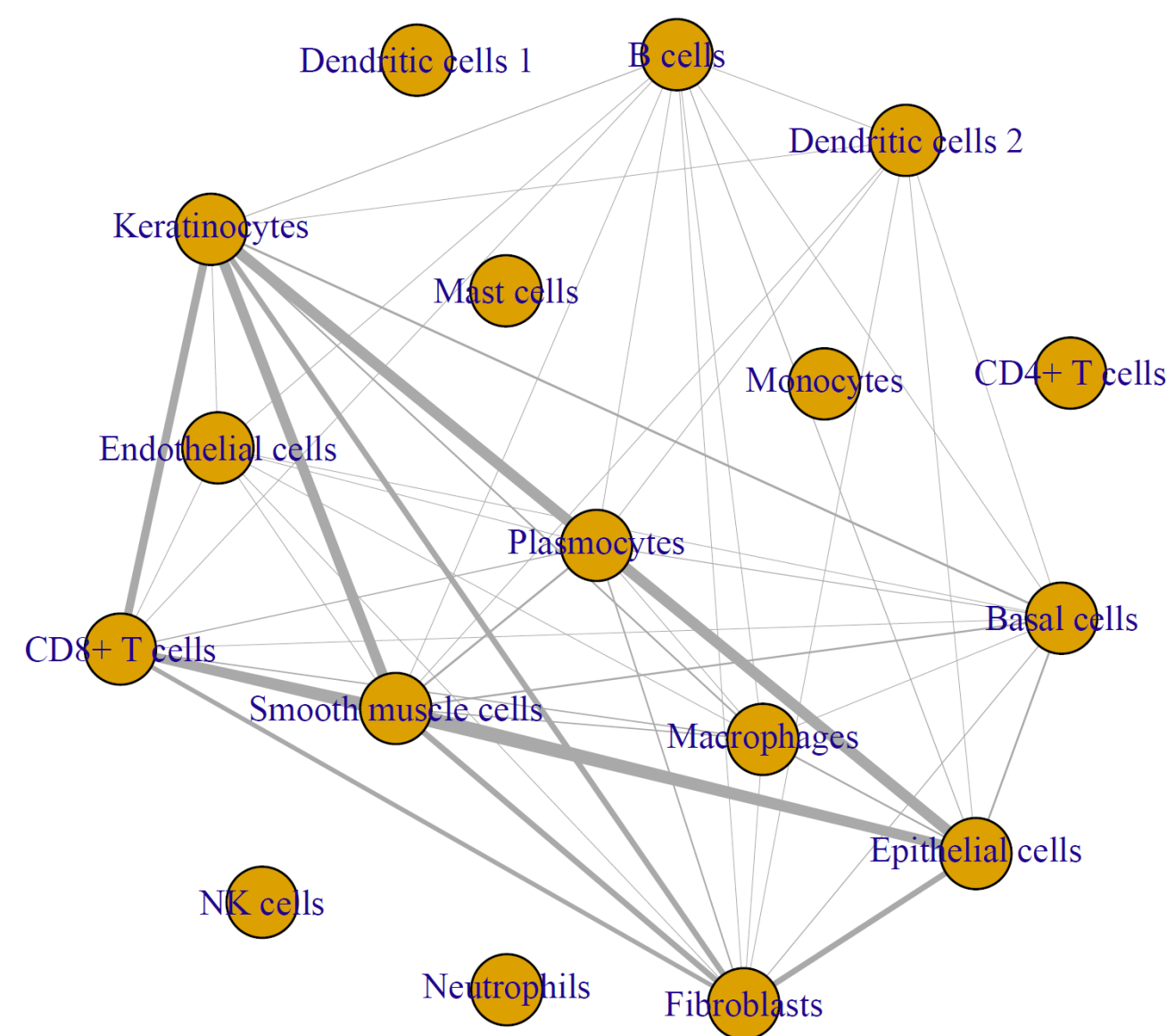

BCST-3

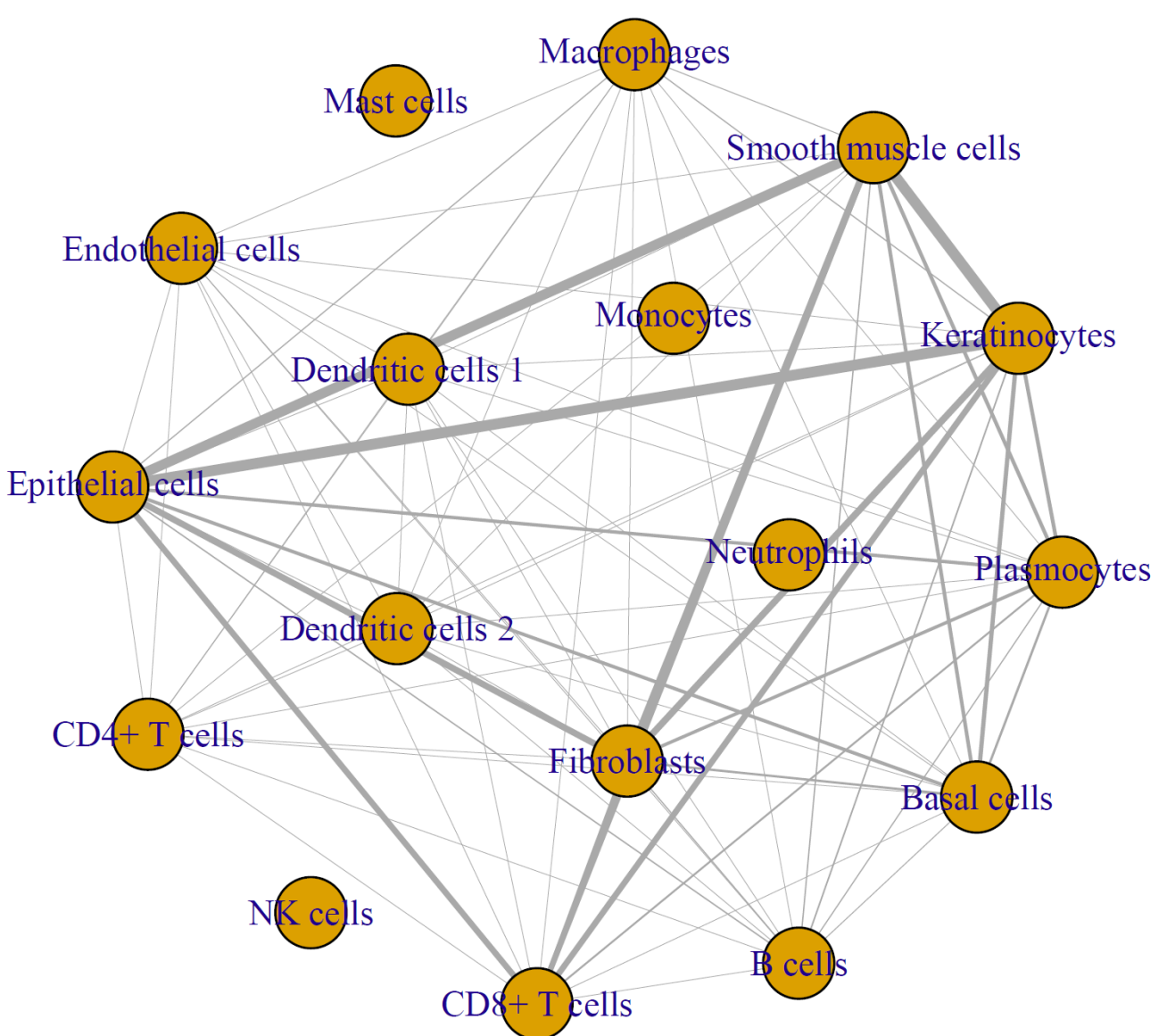

BCST-4

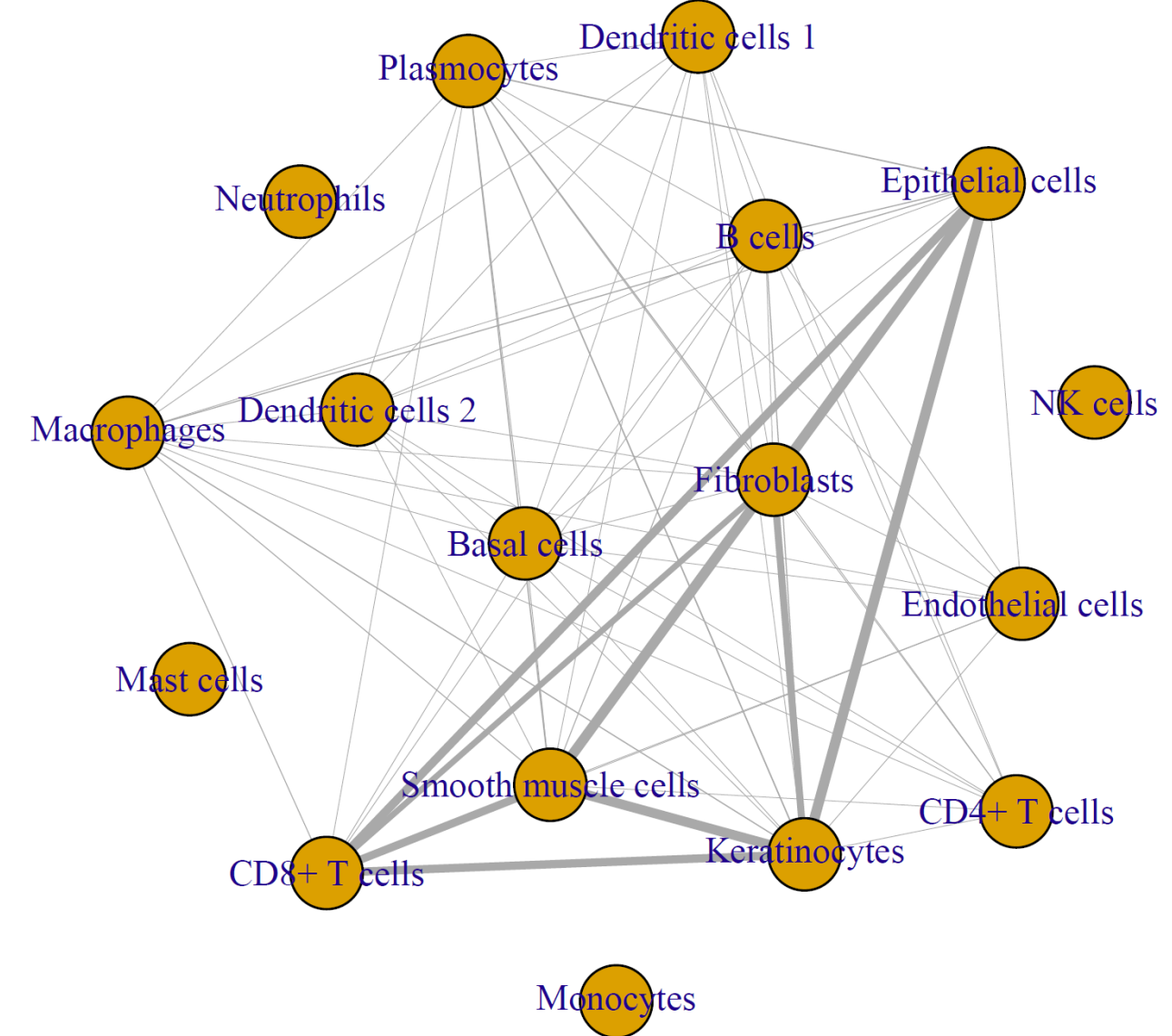

BCST-5

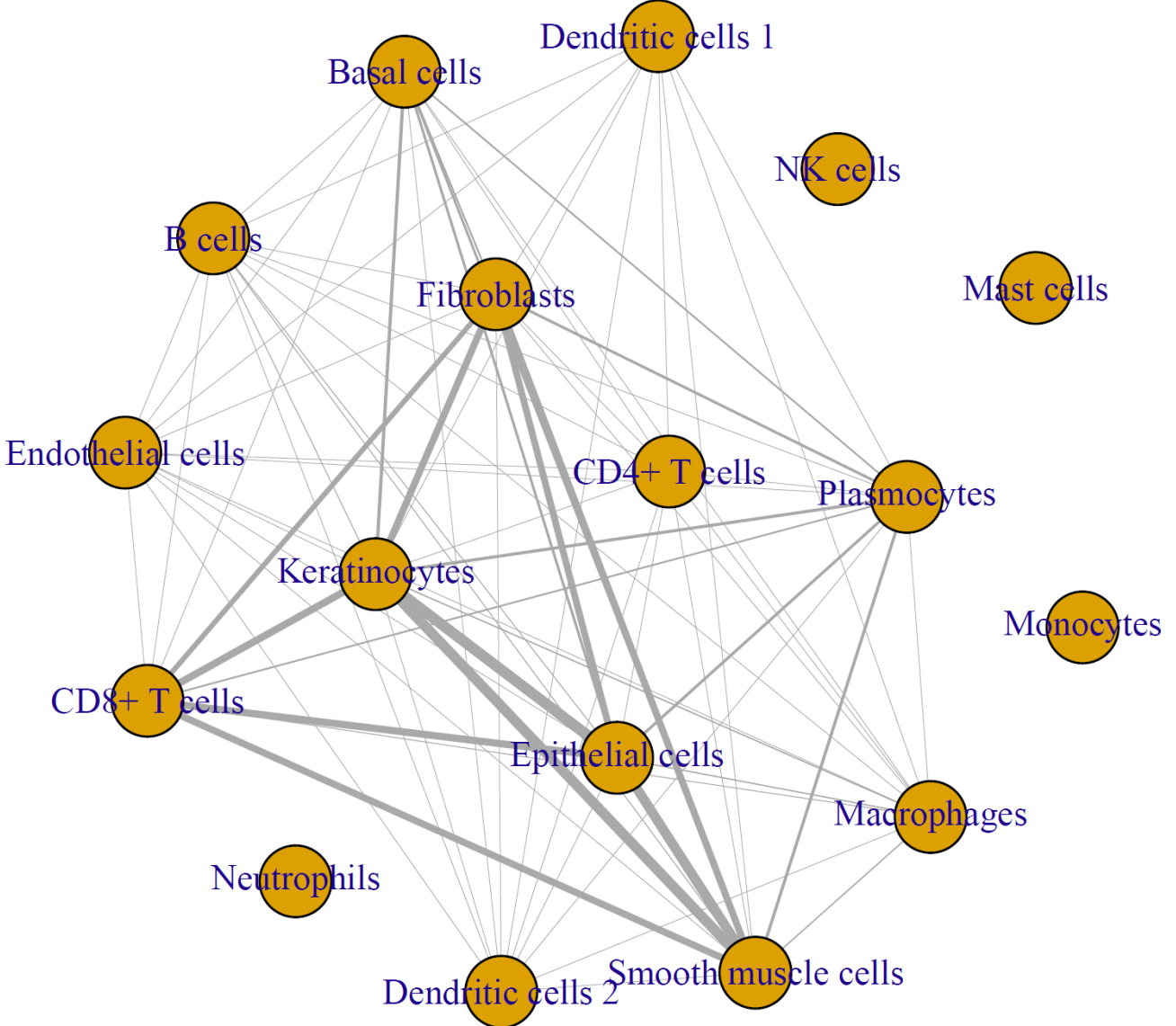

BCST-6

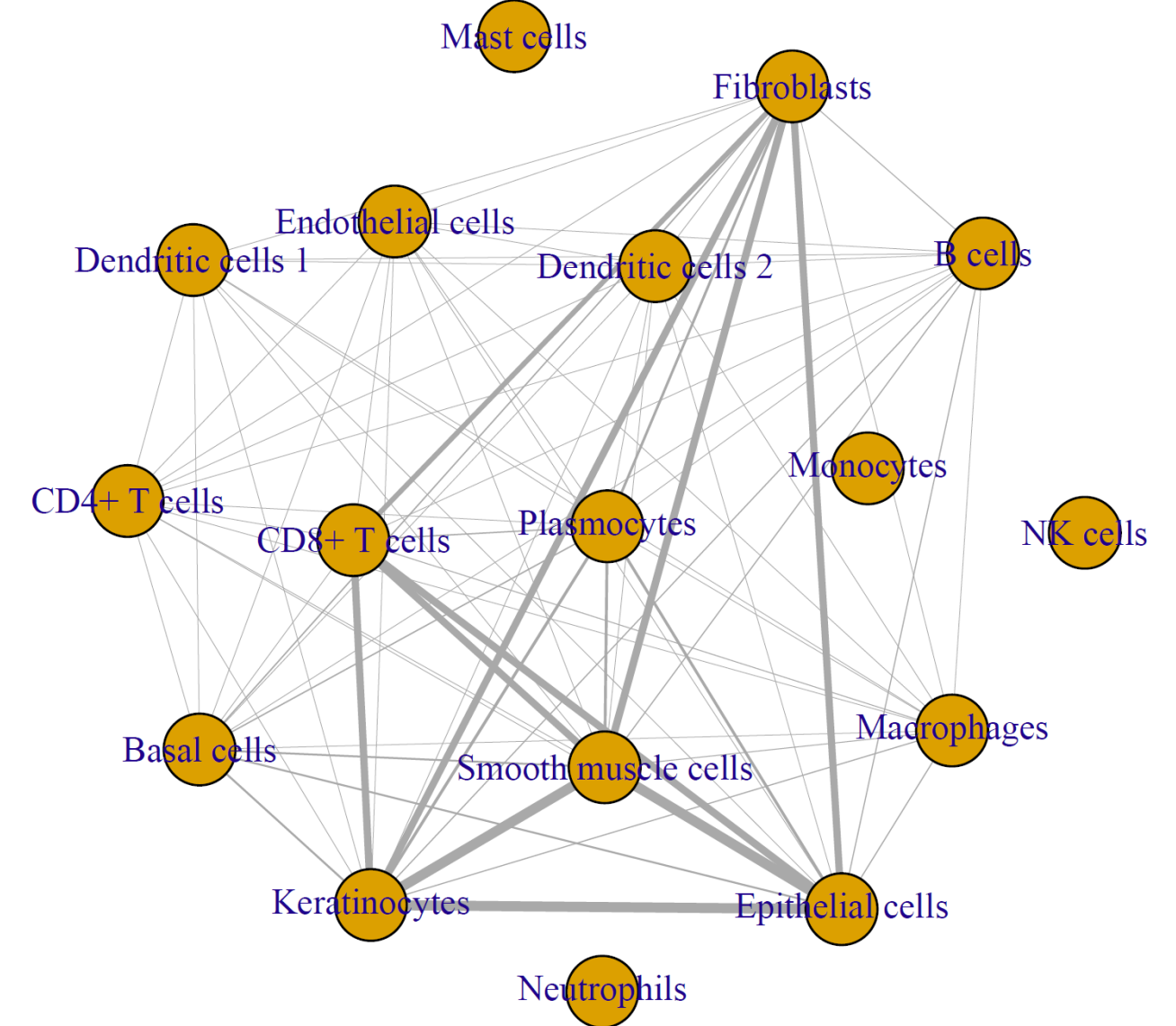

Supplement: Supplementary file 10 — Supporting Information [file CTM2-13-e1338-s006.pdf]

BCST-1

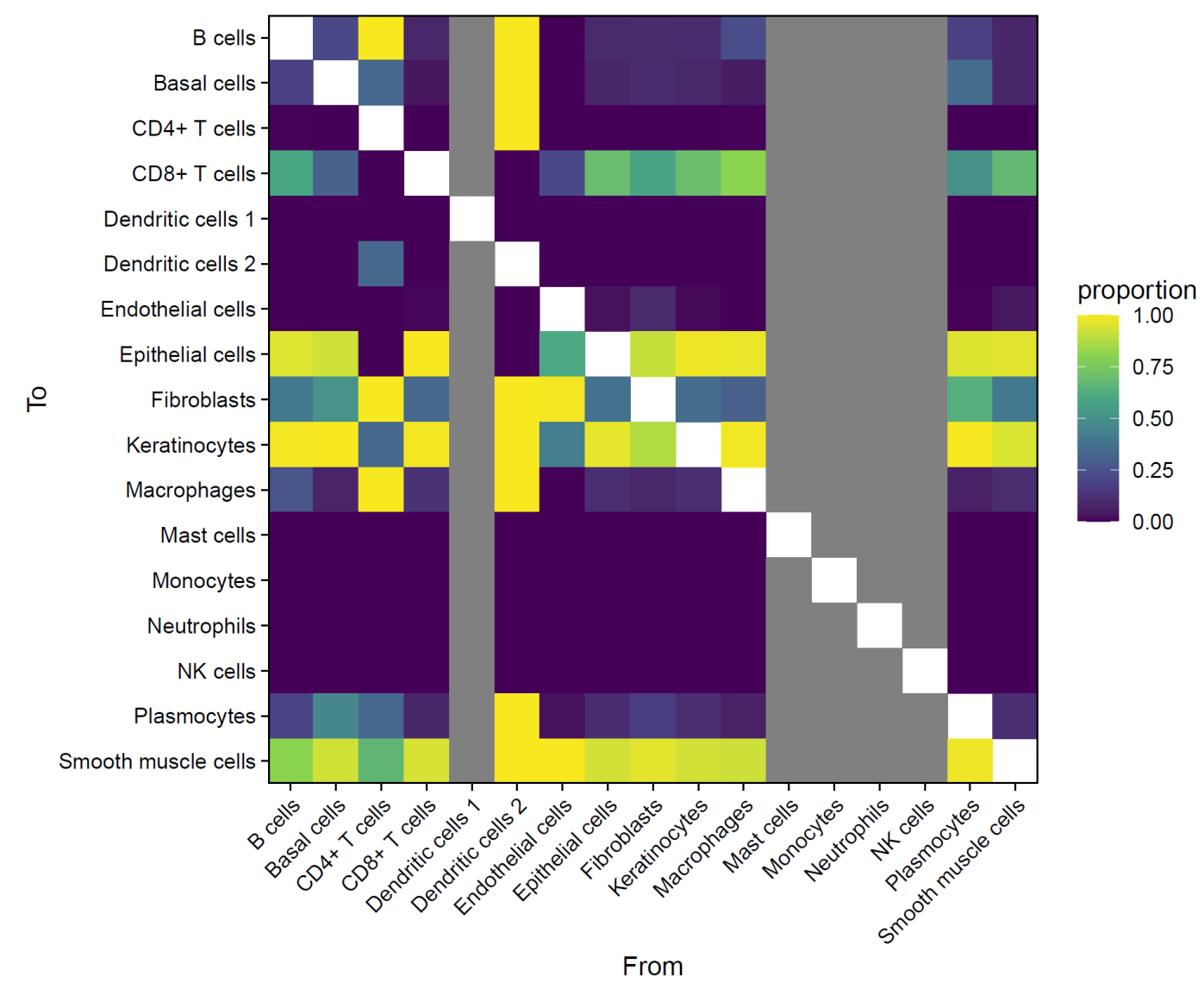

BCST-2

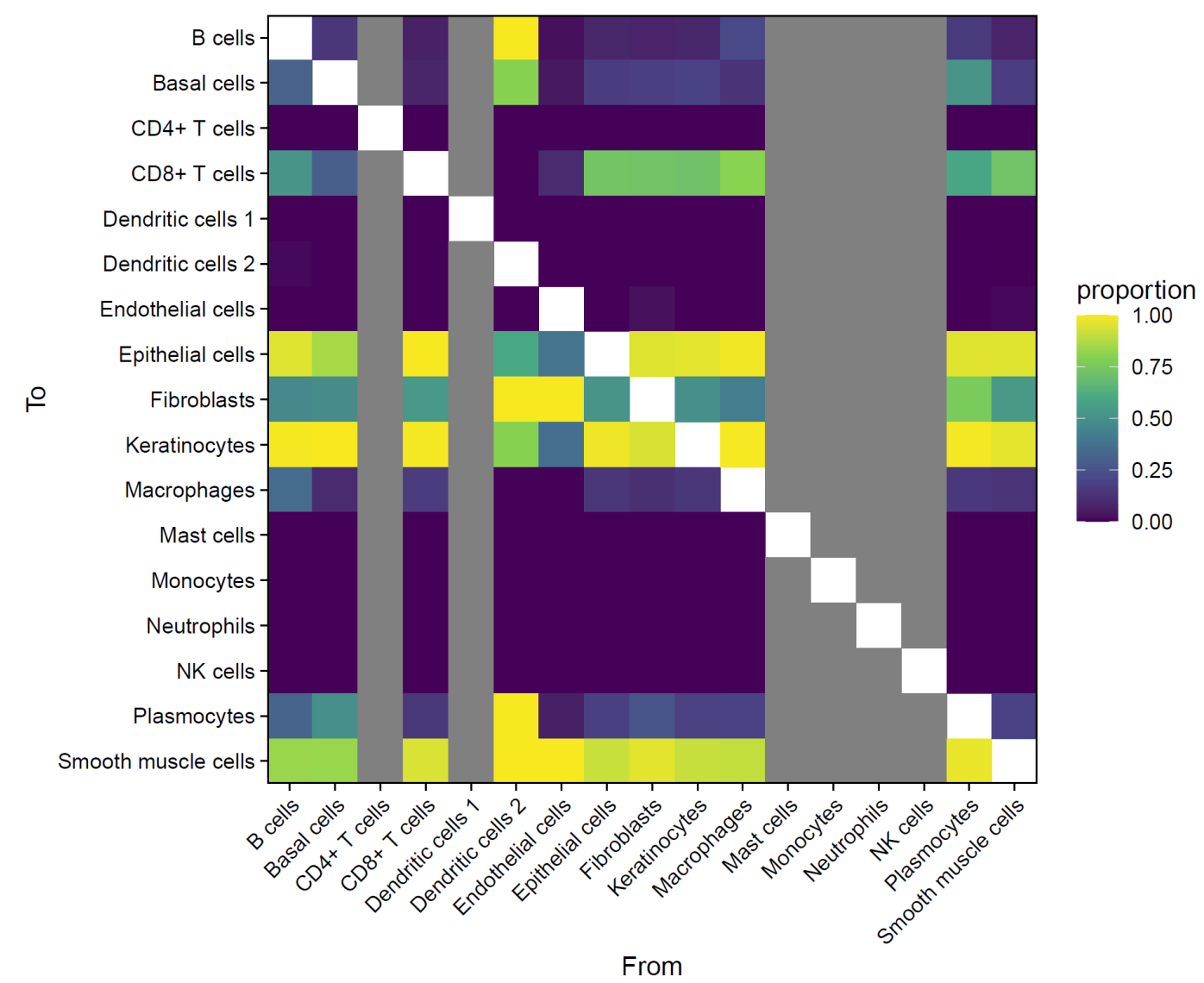

BCST-3

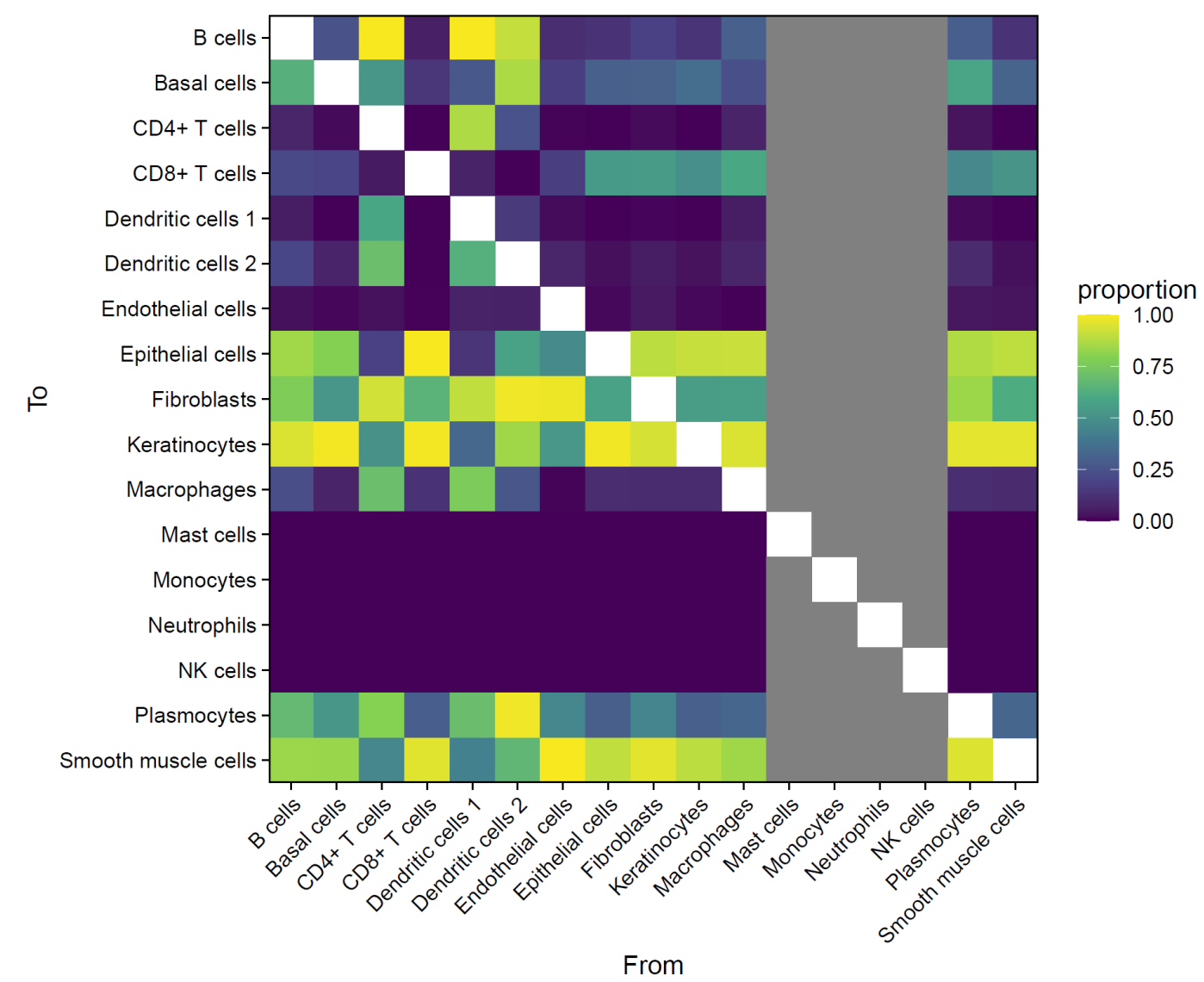

BCST-4

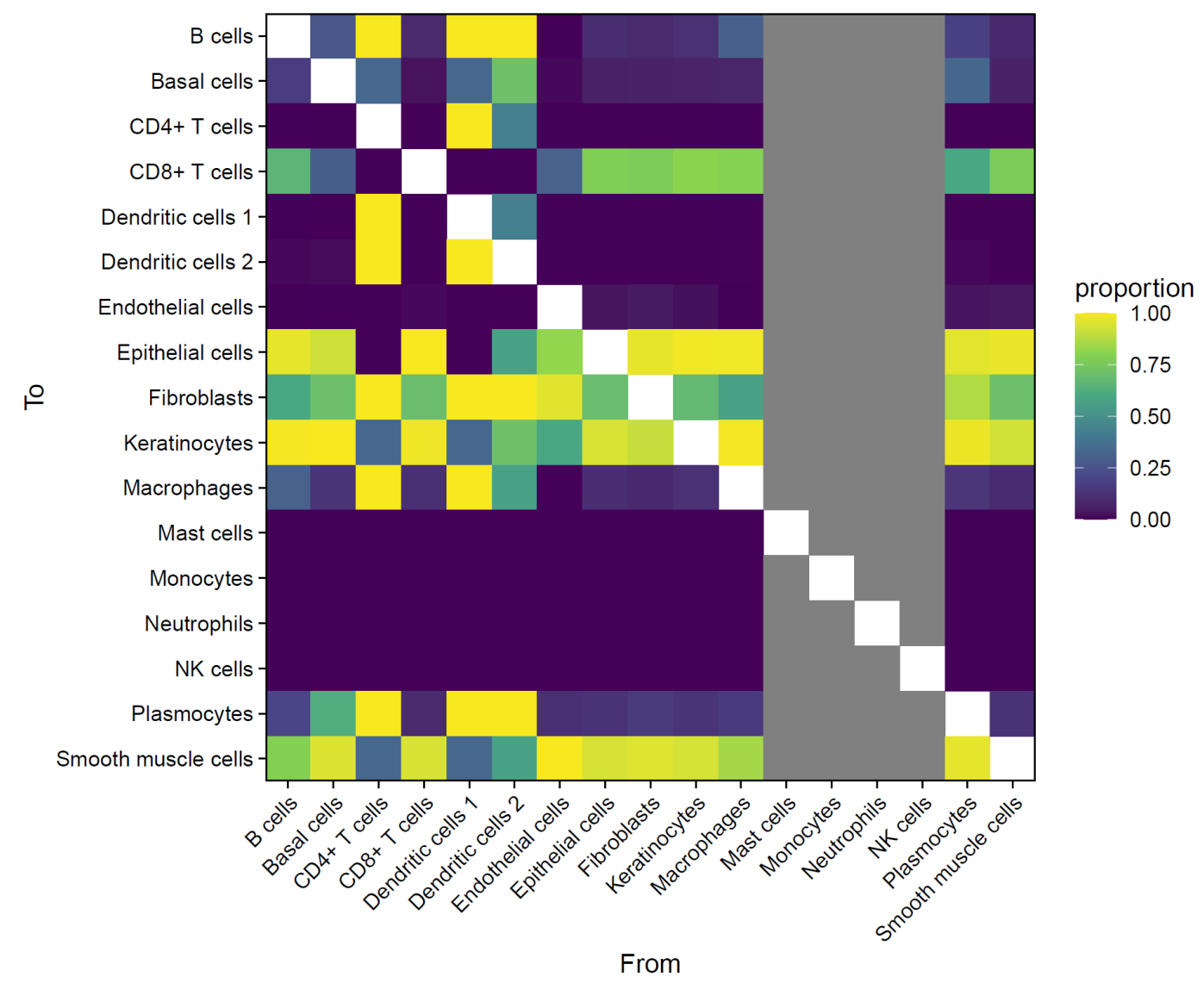

BCST-5

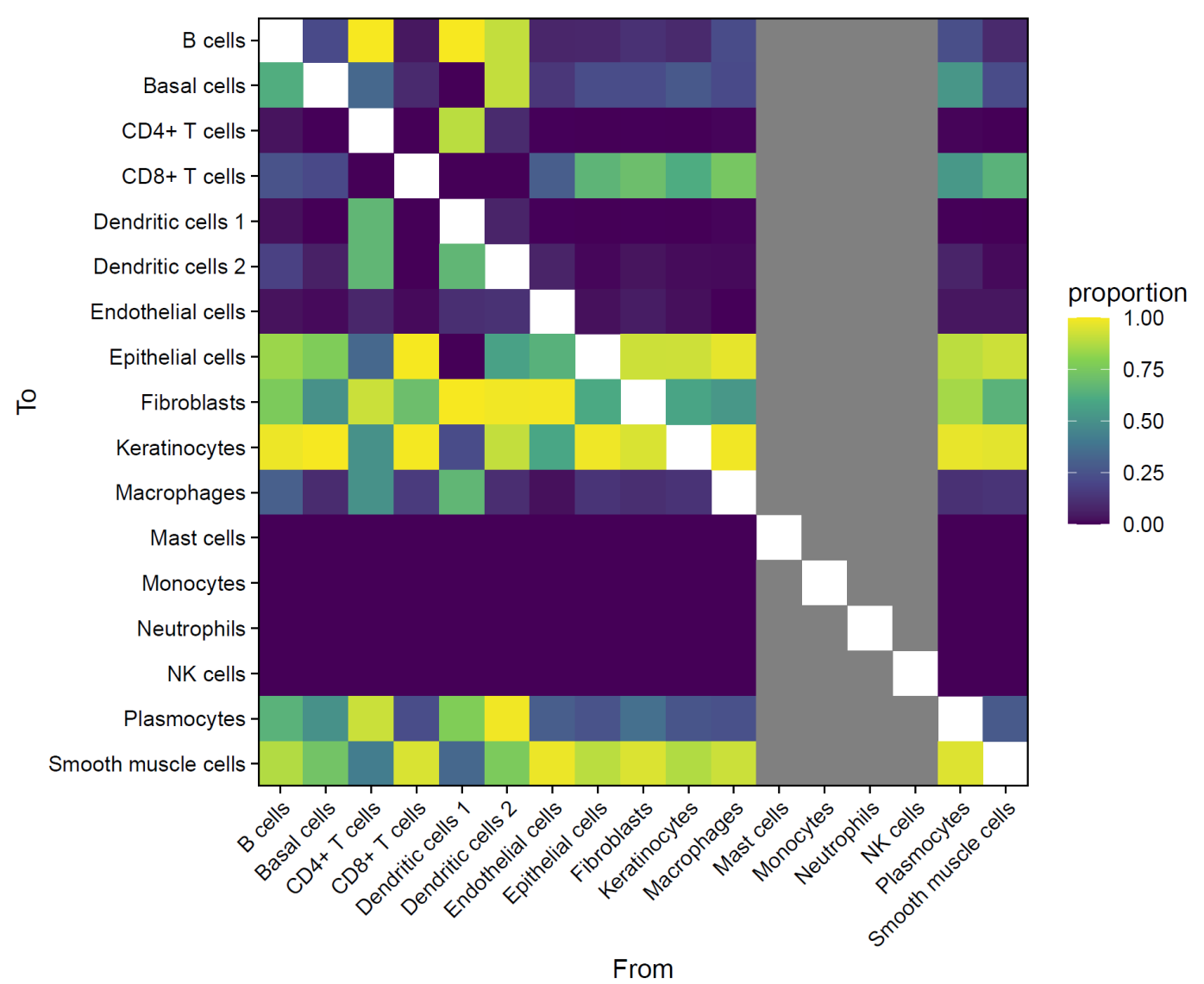

BCST-6

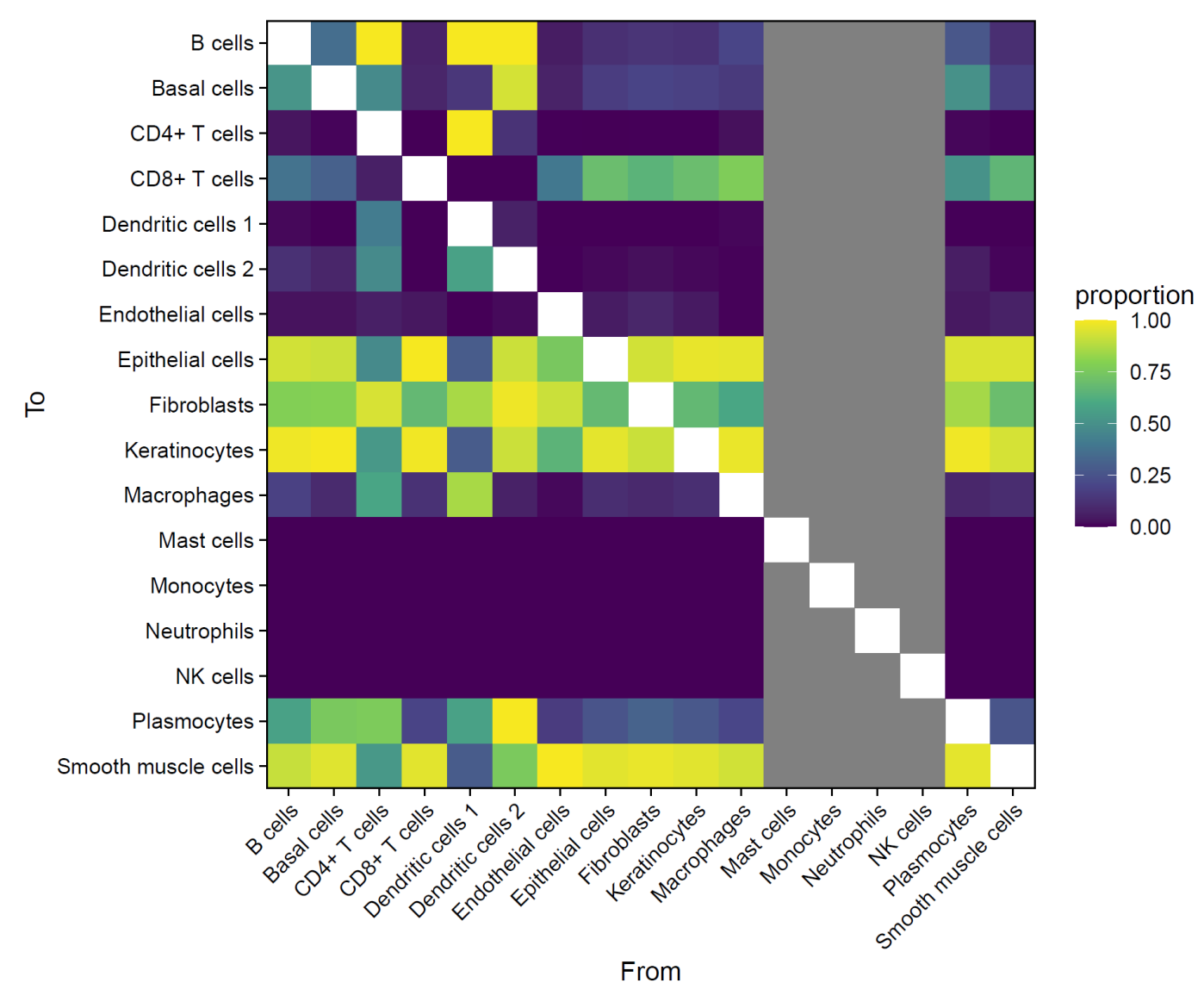

Supplement: Supplementary file 11 — Supporting Information [file CTM2-13-e1338-s011.pdf]

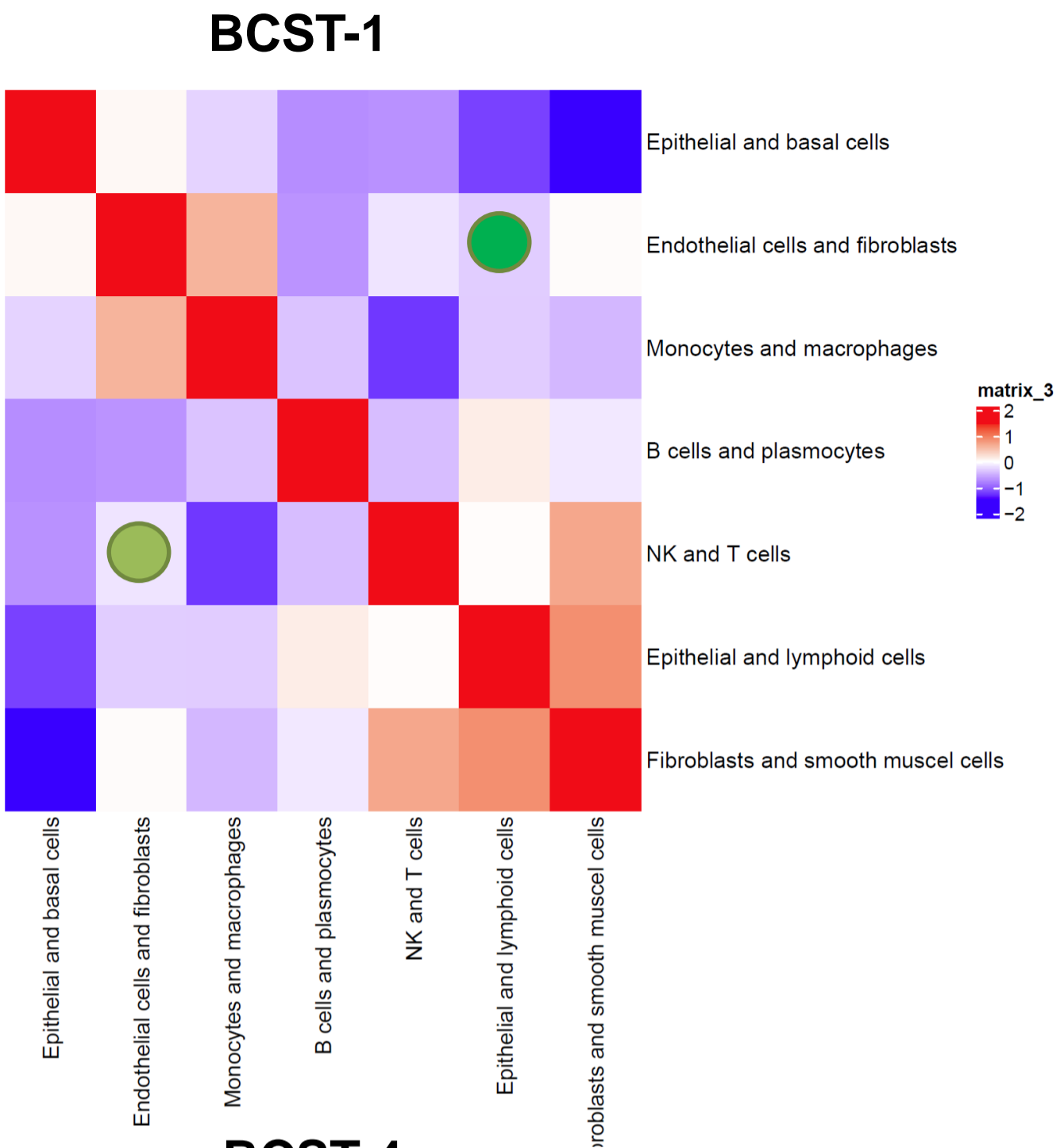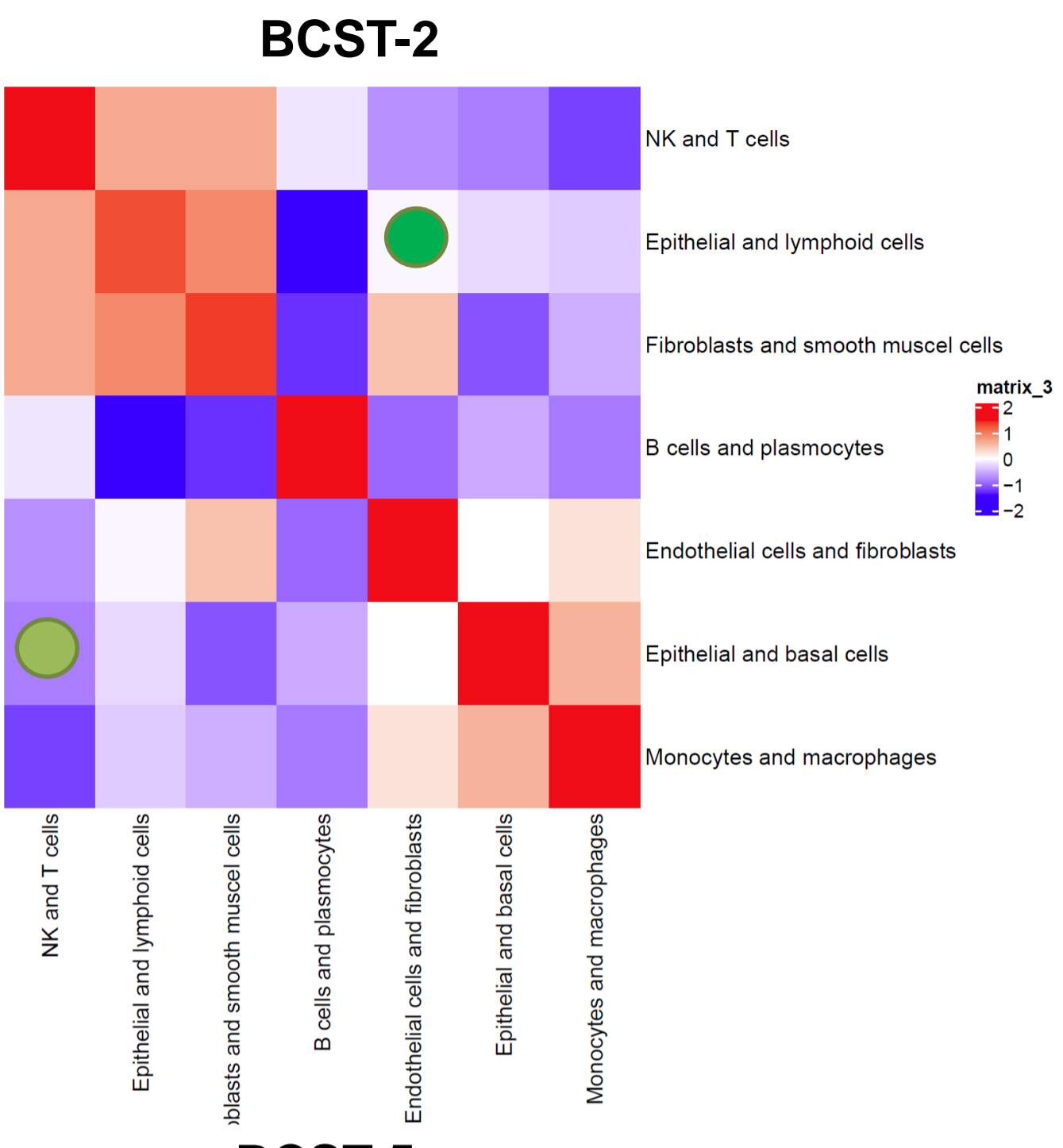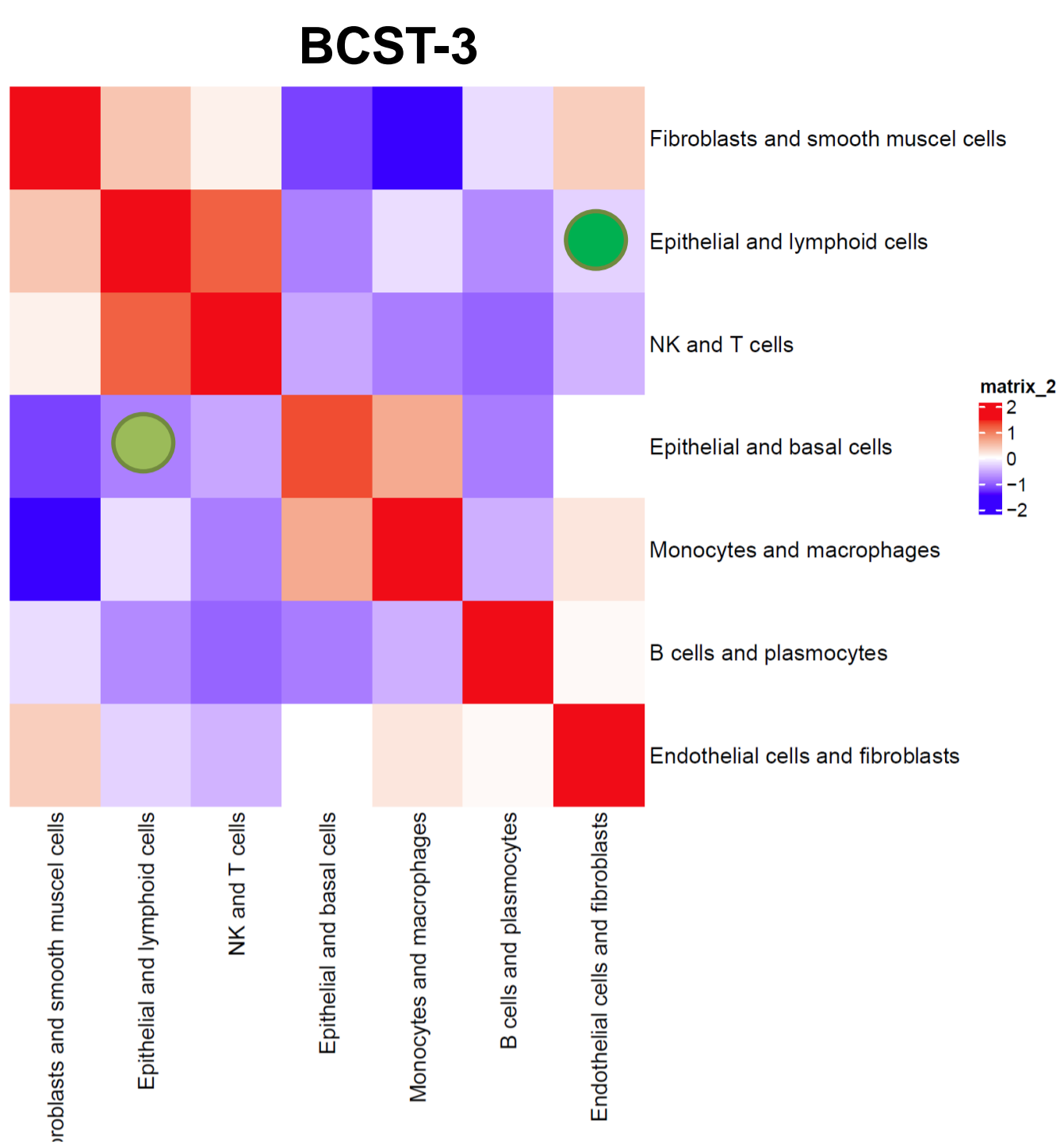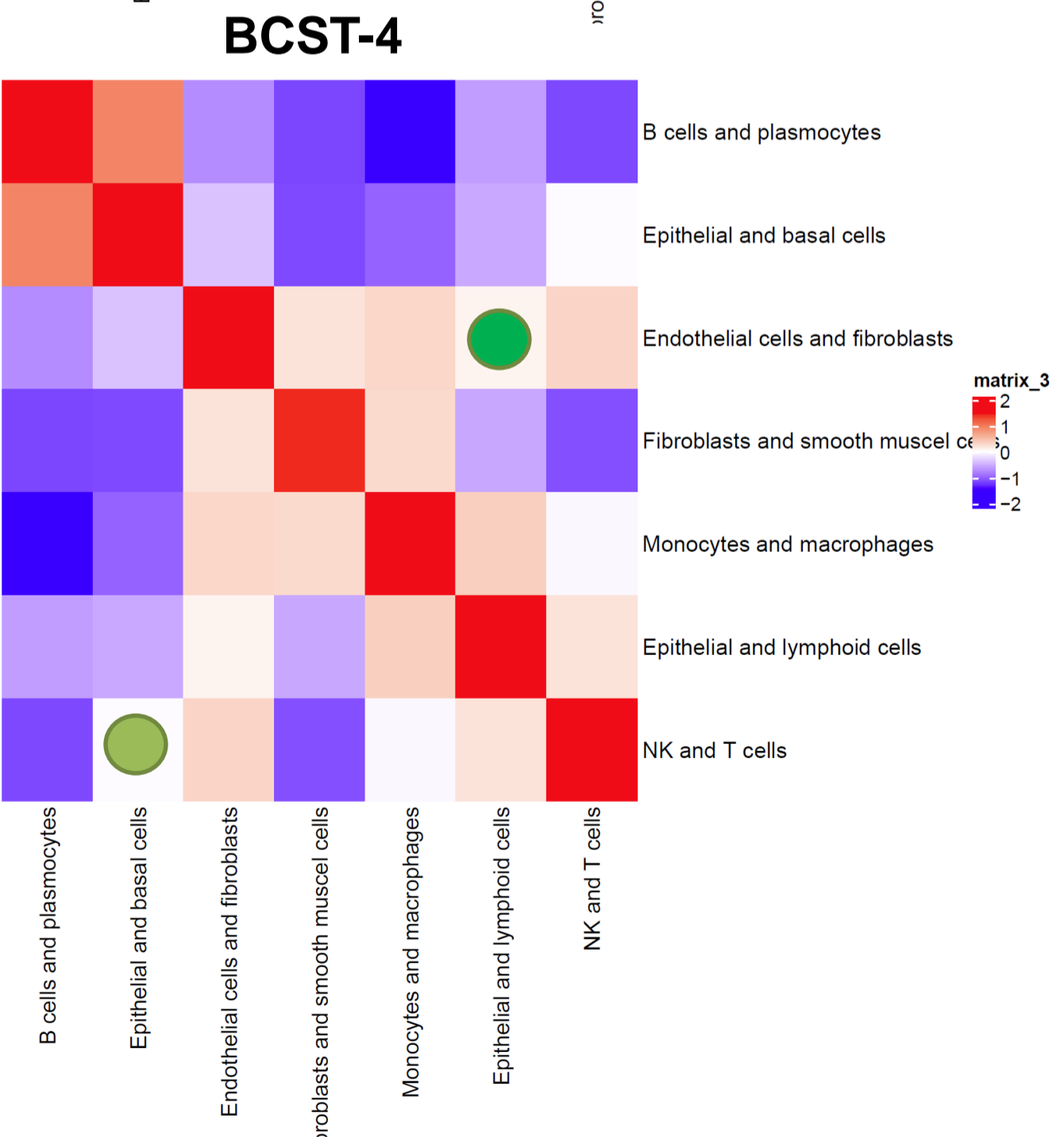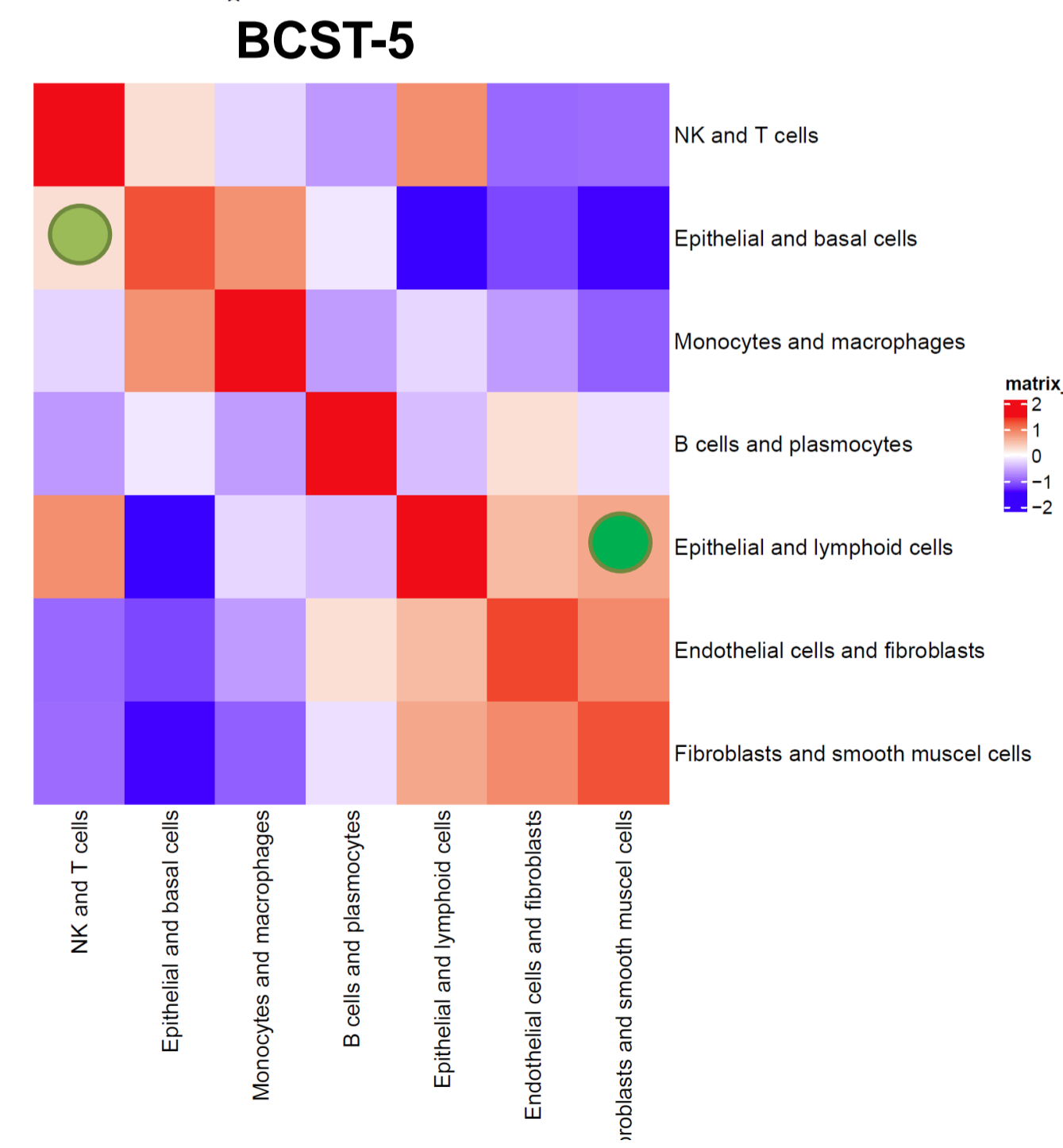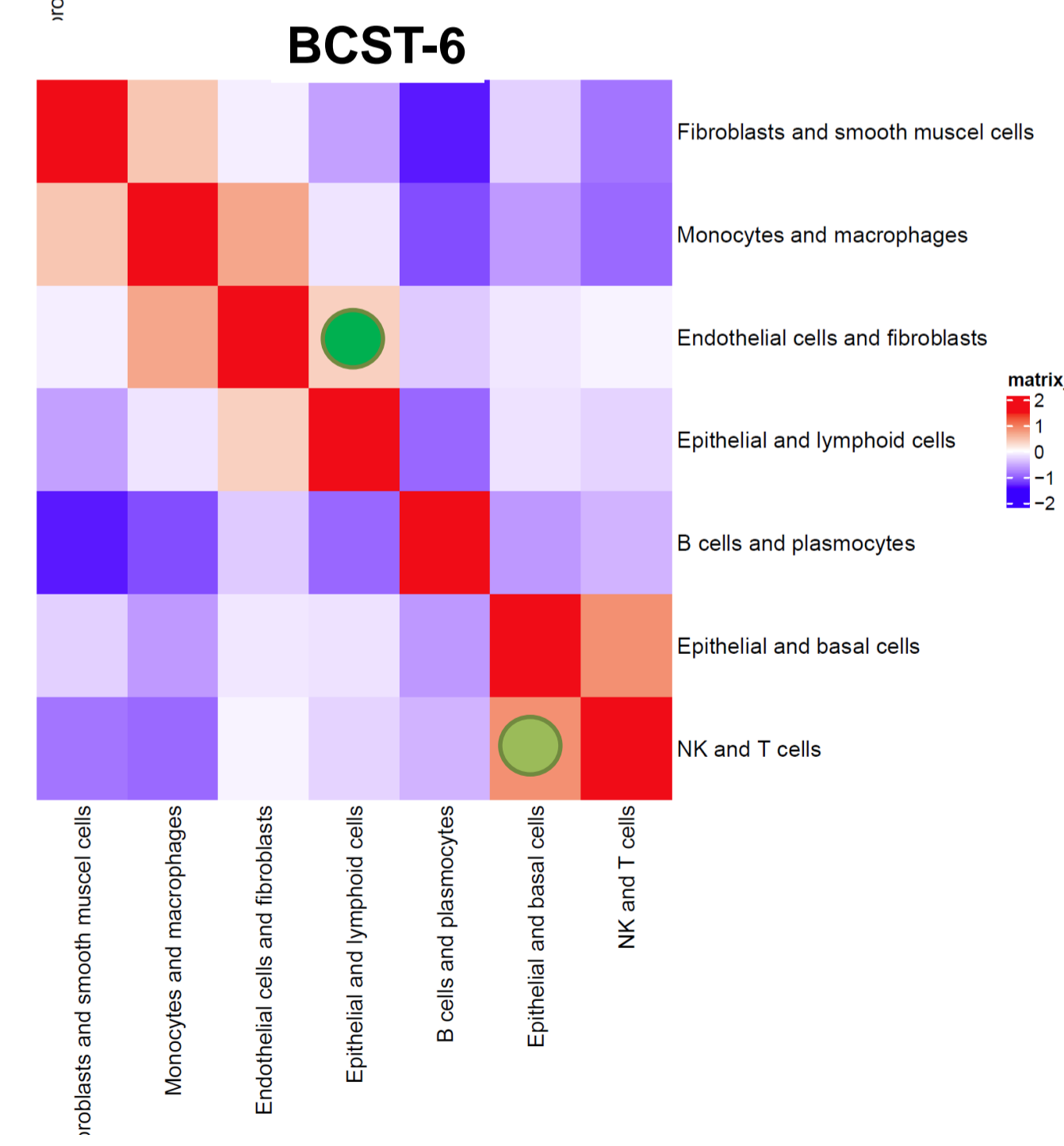

Supplement: Supplementary file 12 — Supporting Information [file CTM2-13-e1338-s008.pdf]

# BCST-1

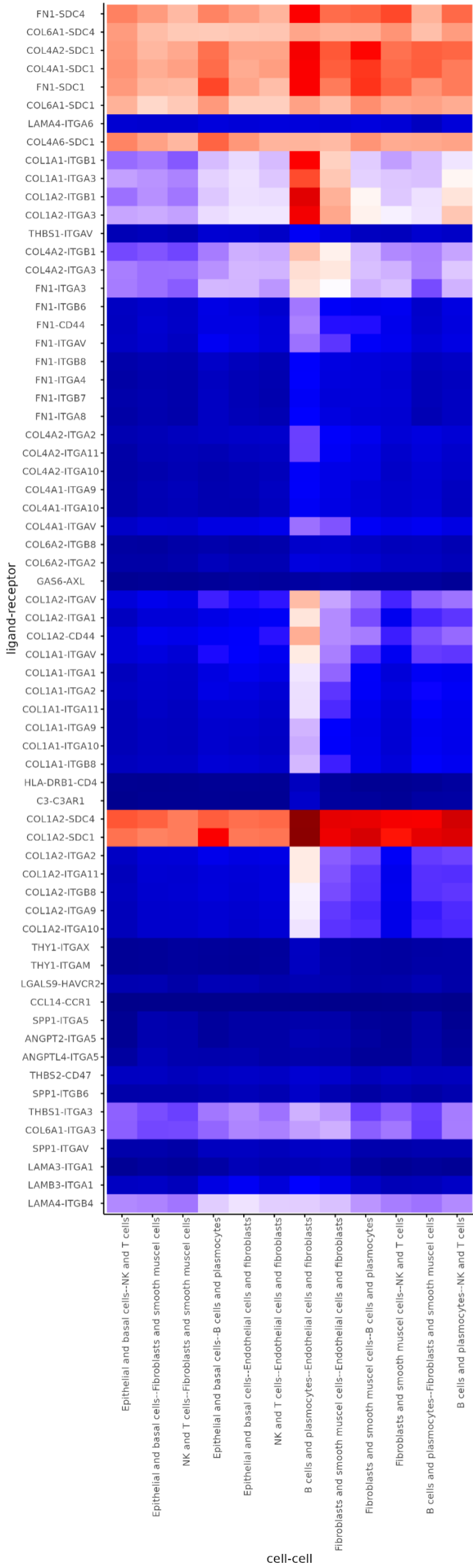

# BCST-2

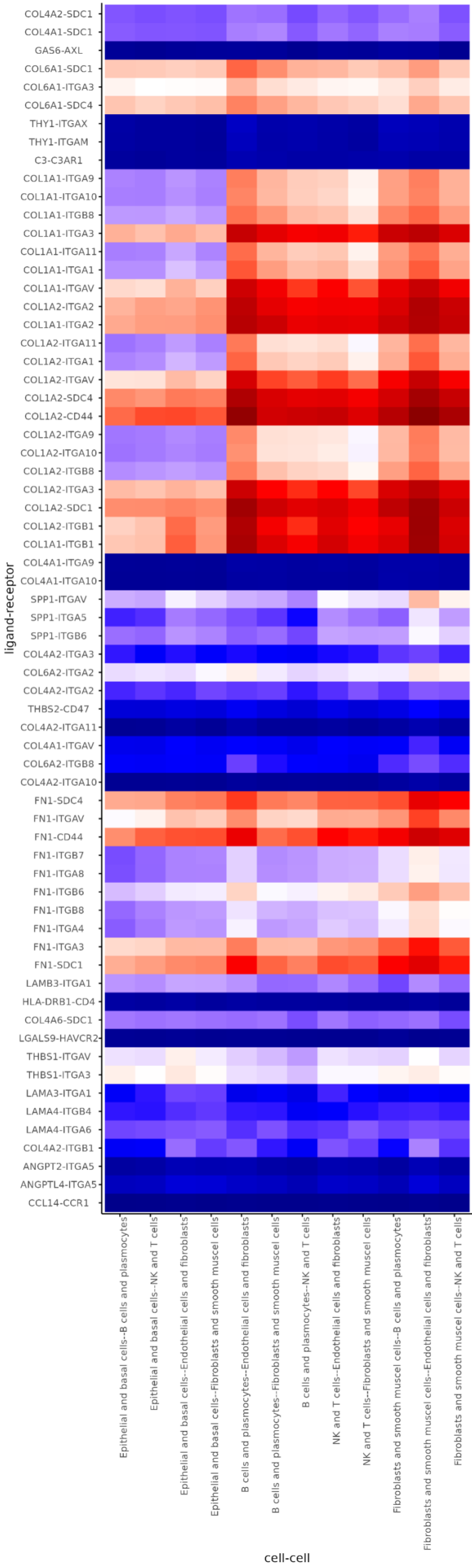

# BCST-3

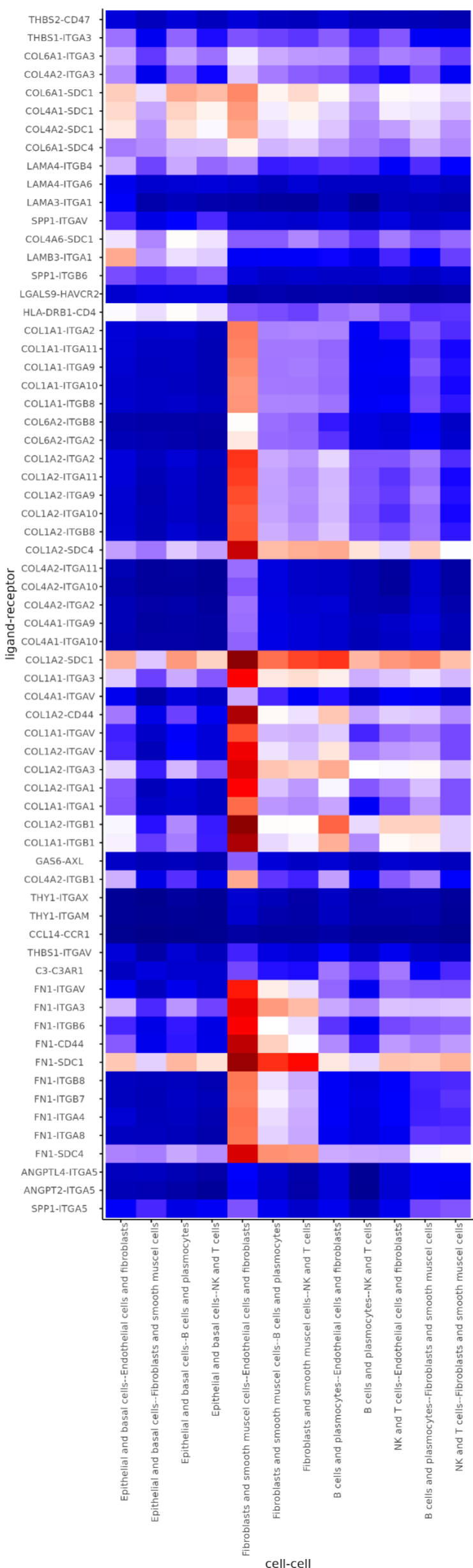

# BCST-4

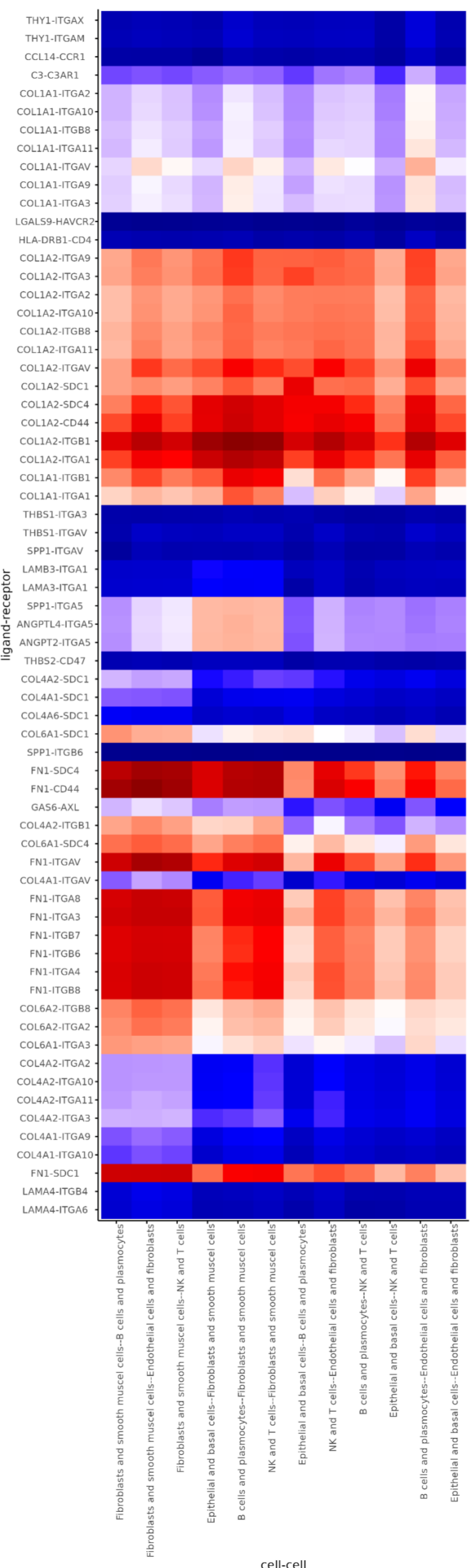

# BCST-5

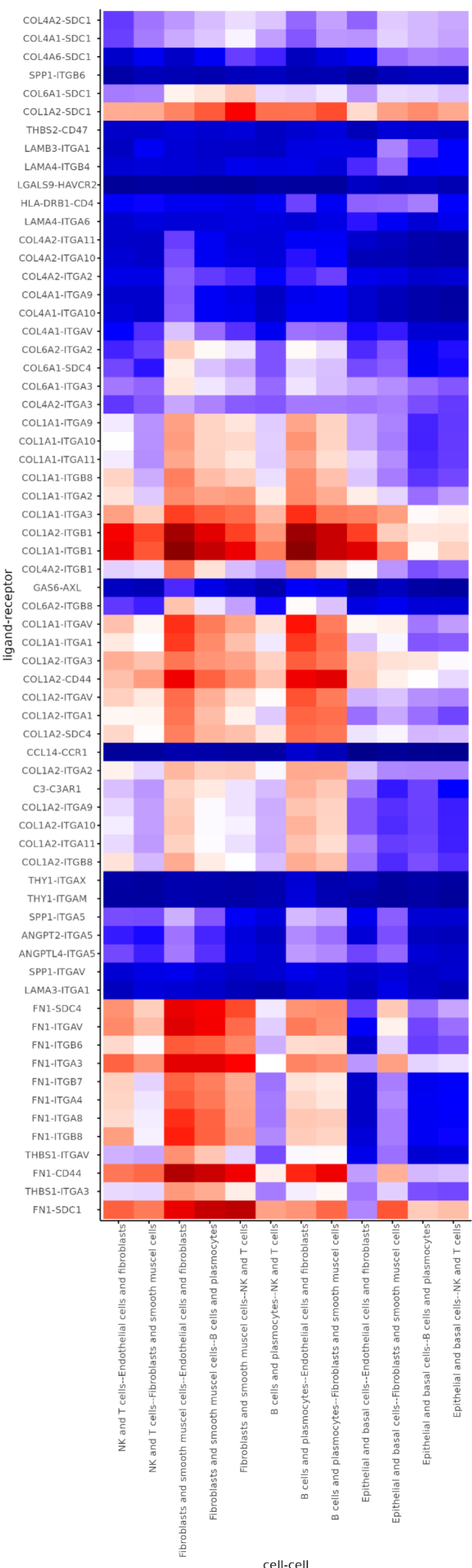

# BCST-6

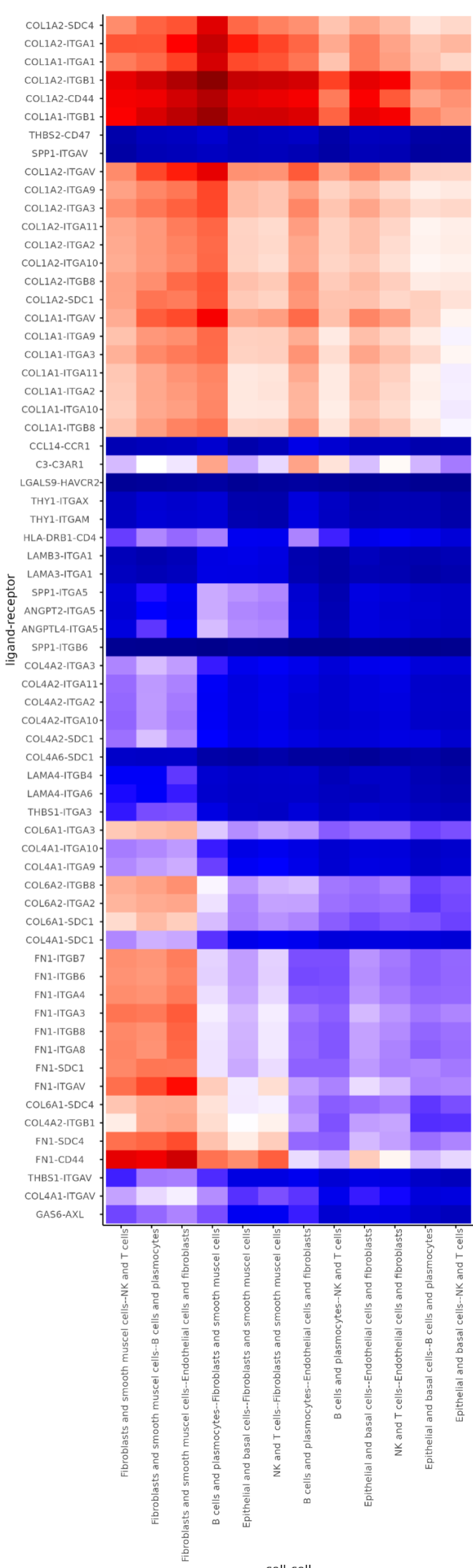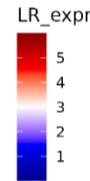

Supplement: Supplementary file 13 — Supporting Information [file CTM2-13-e1338-s014.pdf]

# BCST-1

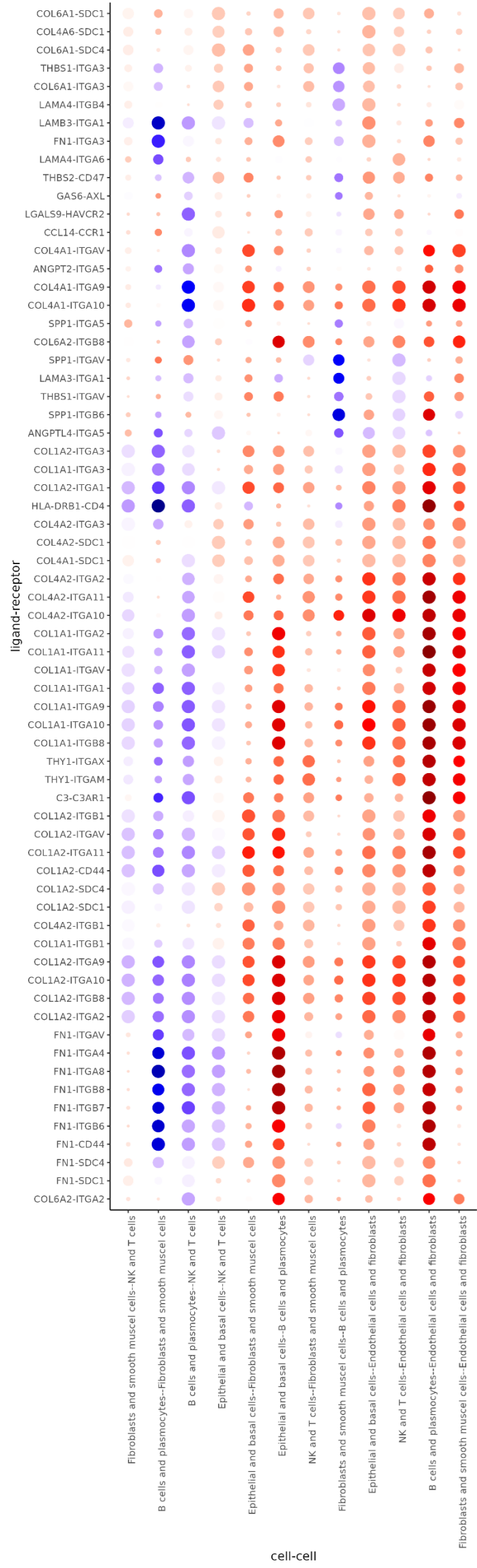

# BCST-2

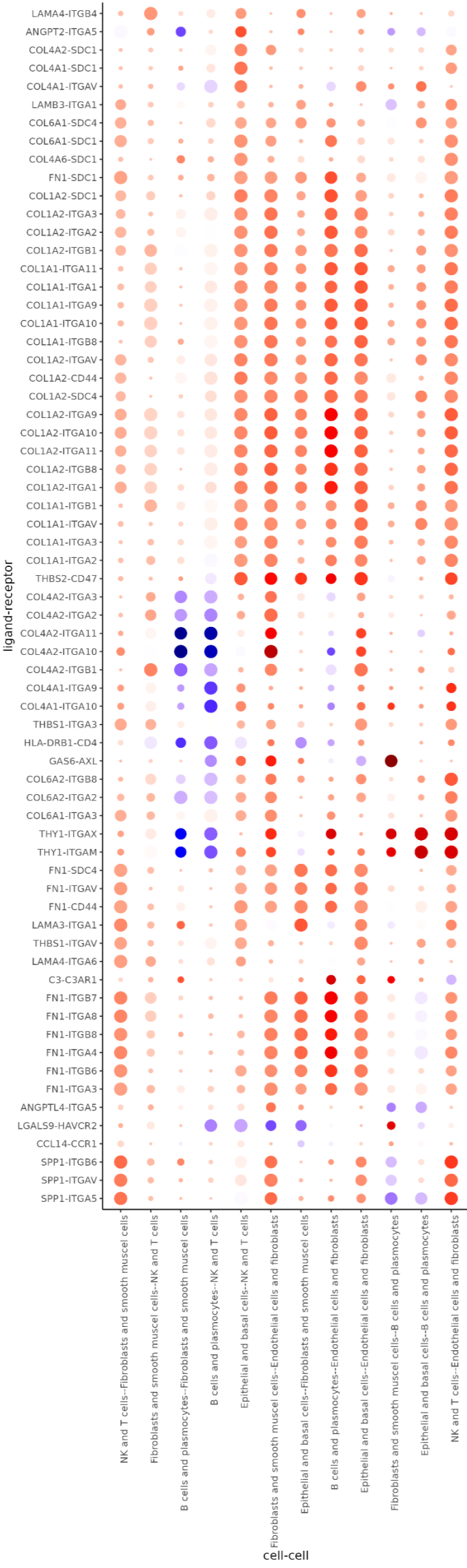

# BCST-3

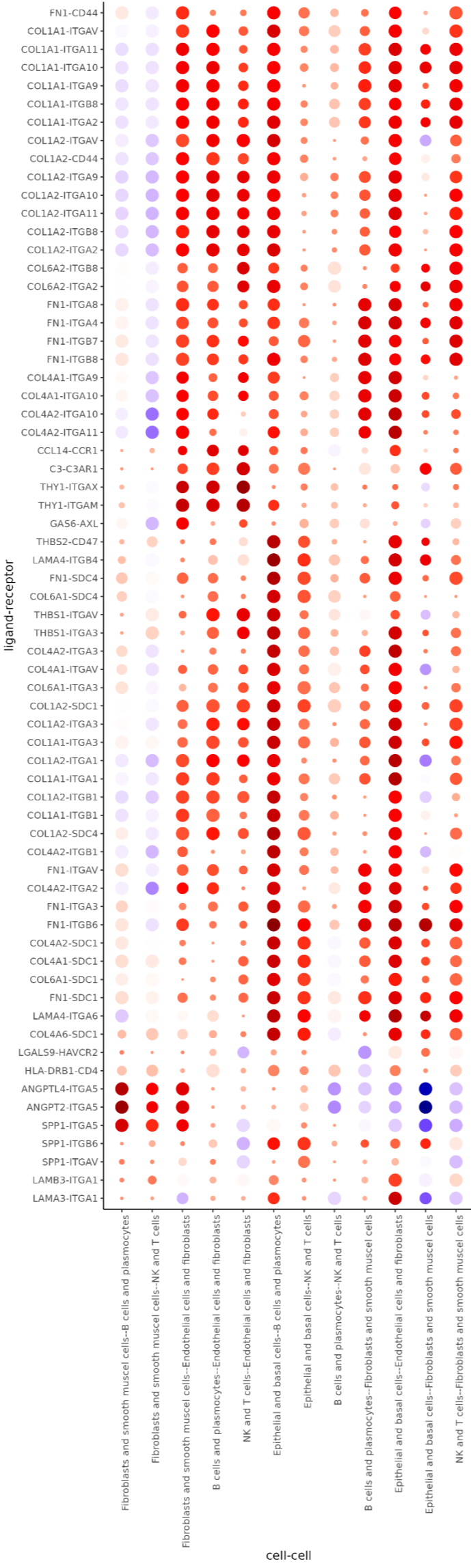

# BCST-4

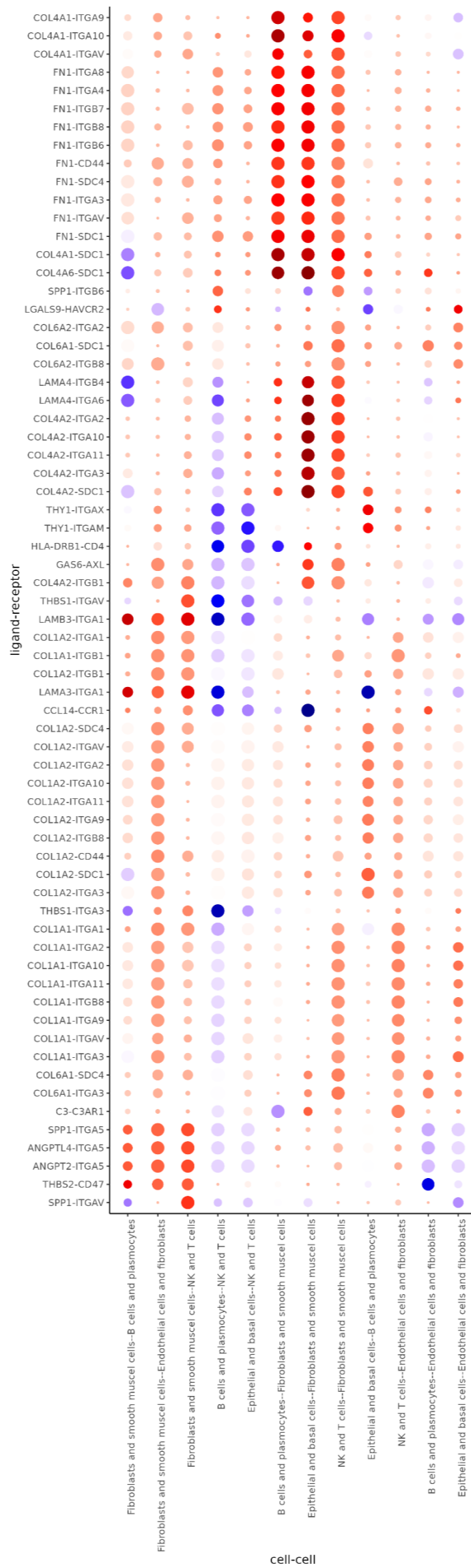

# BCST-5

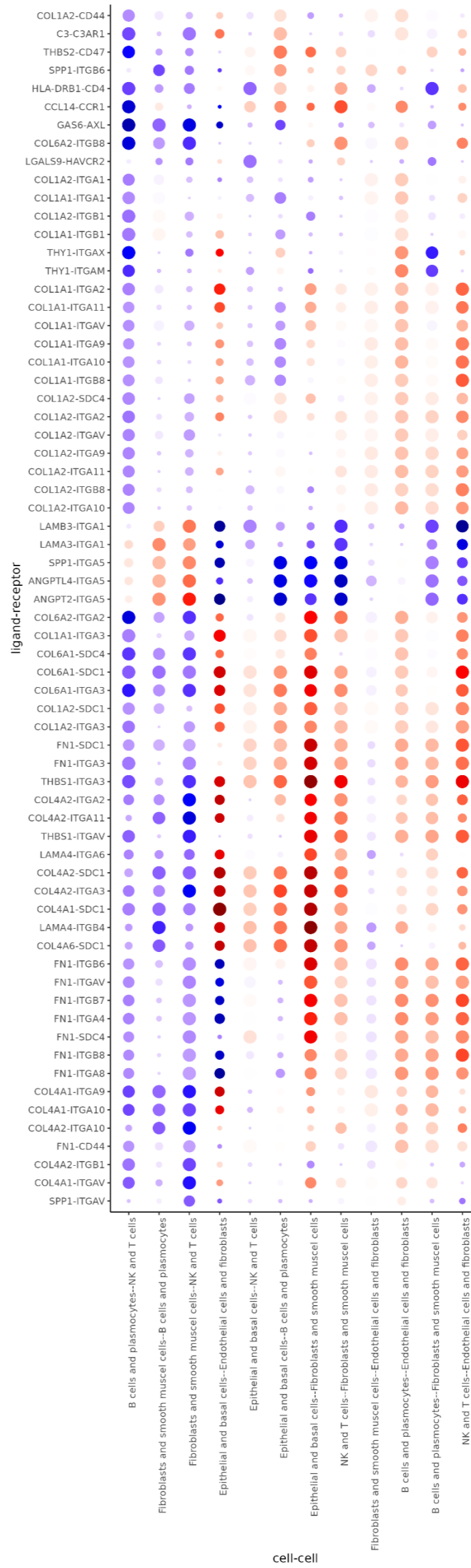

# BCST-6

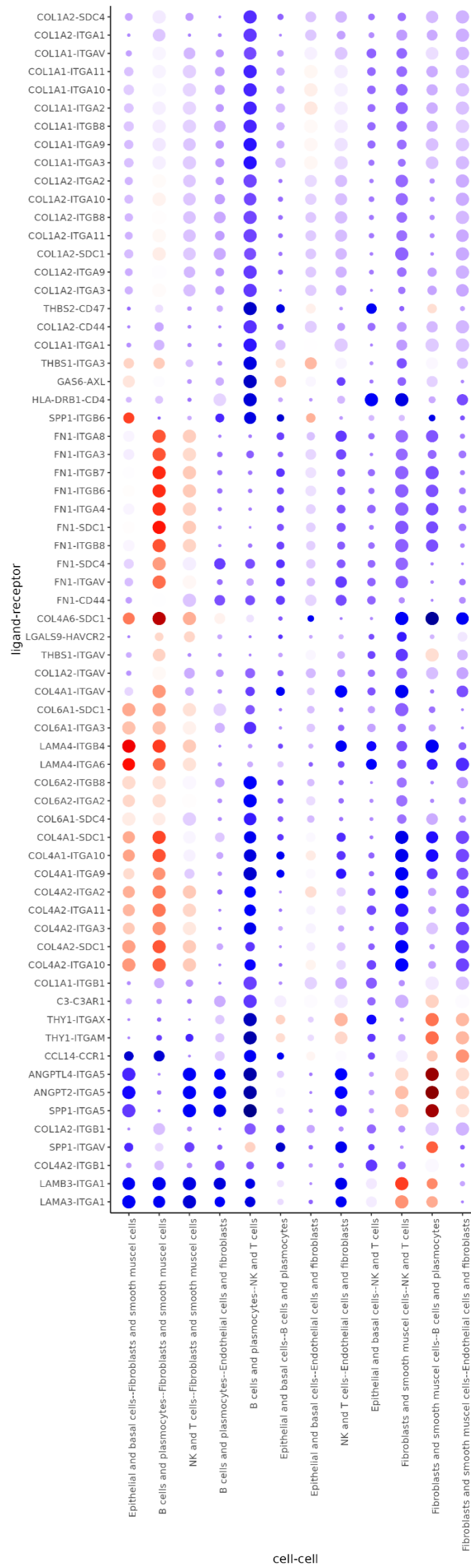

Supplement: Supplementary file 14 — Supporting Information [file CTM2-13-e1338-s012.pdf]
