# Supplementary material for: Integrated single‐cell and spatial transcriptomic profiling reveals higher intratumour heterogeneity and epithelial–fibroblast interactions in recurrent bladder cancer
Source: Clin Transl Med. 2023 Jul 24;13(7):e1338. doi: 10.1002/ctm2.1338 (PMC10366350; doi:10.1002/ctm2.1338)
Supplement: Supplementary file 8 — Supporting Information [file CTM2-13-e1338-s009.pdf]

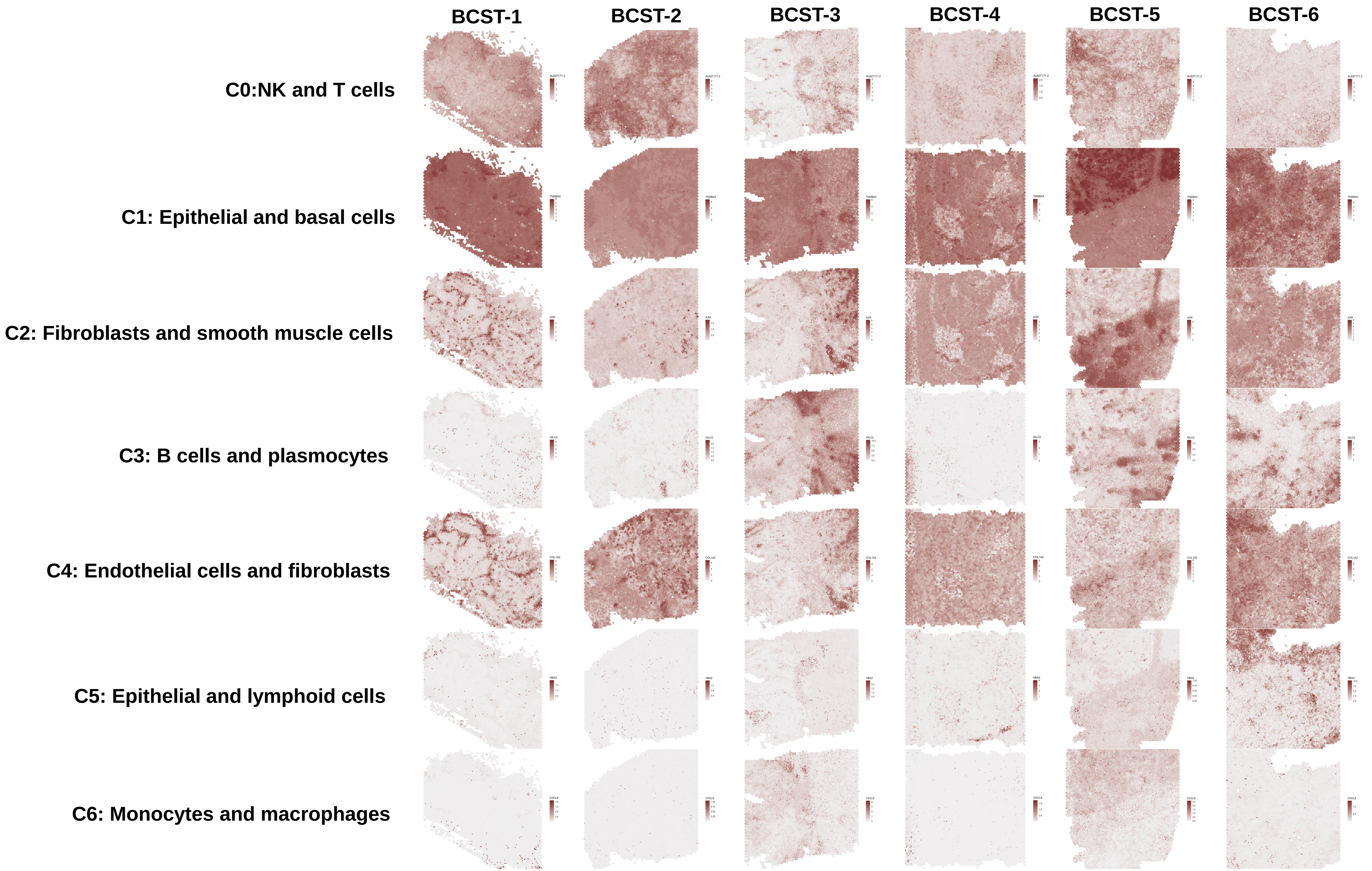
BCST-1
BCST-2
BCST-3
BCST-4
BCST-5
BCST-6
C0:NK and T cells
C1: Epithelial and basal cells
C2: Fibroblasts and smooth muscle cells
C3: B cells and plasmocytes
C4: Endothelial cells and fibroblasts
C5: Epithelial and lymphoid cells
C6: Monocytes and macrophages
