# Supplementary material for: Integrated single‐cell and spatial transcriptomic profiling reveals higher intratumour heterogeneity and epithelial–fibroblast interactions in recurrent bladder cancer
Source: Clin Transl Med. 2023 Jul 24;13(7):e1338. doi: 10.1002/ctm2.1338 (PMC10366350; doi:10.1002/ctm2.1338)
Supplement: Supplementary file 9 — Supporting Information [file CTM2-13-e1338-s013.pdf]

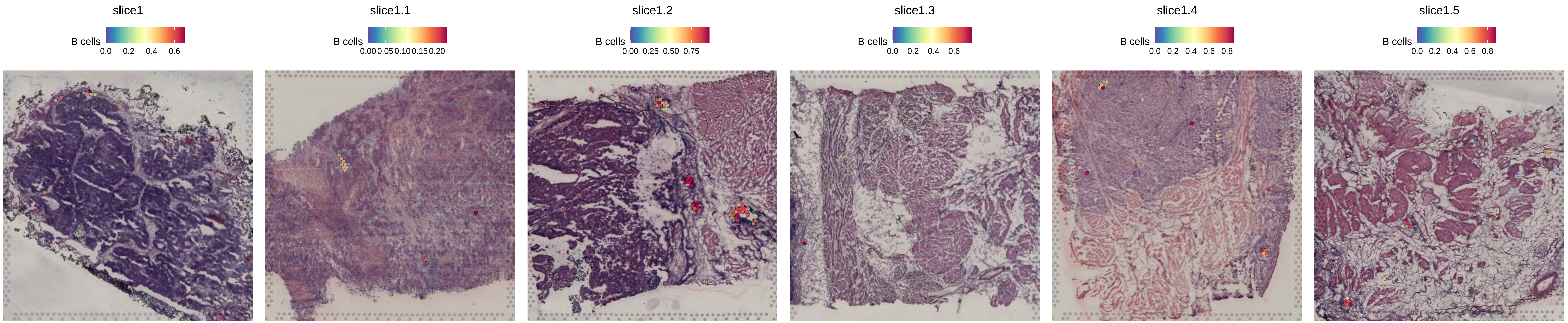

slice1
B cells
0.0
0.2
0.4
0.6
slice1.1
B cells
0.00
0.05
0.10
0.15
0.20
slice1.2
B cells
0.00
0.25
0.50
0.75
slice1.3
B cells
0.0
0.2
0.4
0.6
slice1.4
B cells
0.0
0.2
0.4
0.6
0.8
slice1.5
B cells
0.0
0.2
0.4
0.6
0.8

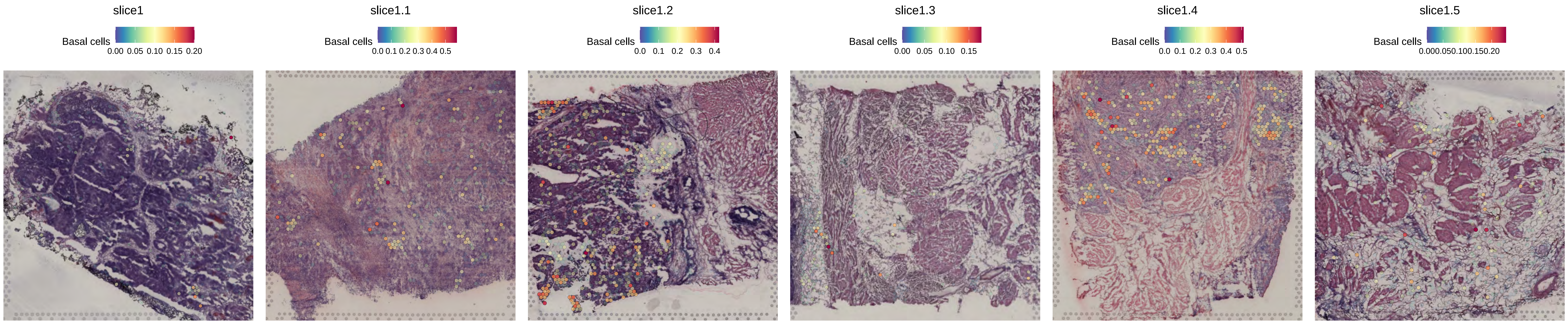
slice1
Basal cells
0.00 0.05 0.10 0.15 0.20
slice1.1
Basal cells
0.0 0.1 0.2 0.3 0.4 0.5
slice1.2
Basal cells
0.0 0.1 0.2 0.3 0.4
slice1.3
Basal cells
0.00 0.05 0.10 0.15
slice1.4
Basal cells
0.0 0.1 0.2 0.3 0.4 0.5
slice1.5
Basal cells
0.000.050.100.150.20

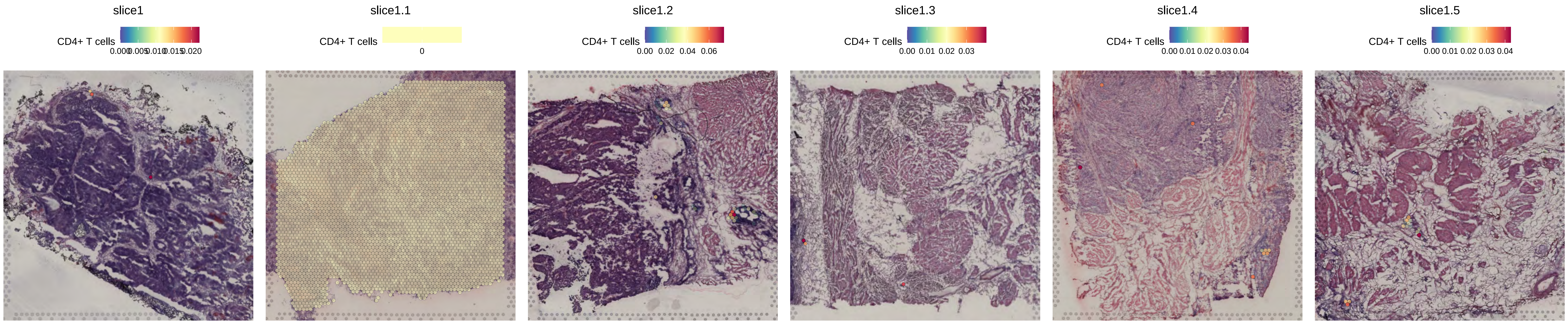

slice1
CD4+ T cells
0.000 0.005 0.010 0.015 0.020
slice1.1
CD4+ T cells
0
slice1.2
CD4+ T cells
0.00 0.02 0.04 0.06
slice1.3
CD4+ T cells
0.00 0.01 0.02 0.03
slice1.4
CD4+ T cells
0.00 0.01 0.02 0.03 0.04
slice1.5
CD4+ T cells
0.00 0.01 0.02 0.03 0.04

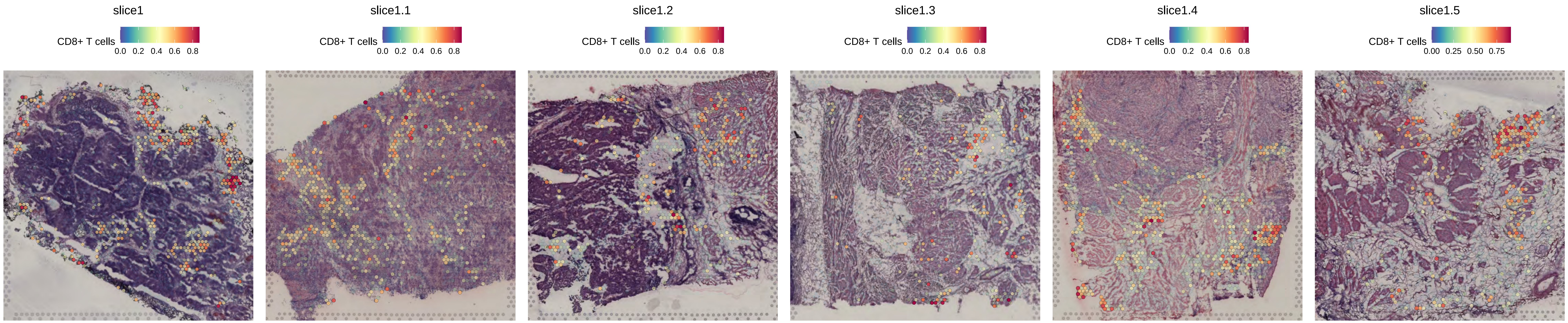
slice1
CD8+ T cells
0.0 0.2 0.4 0.6 0.8
slice1.1
CD8+ T cells
0.0 0.2 0.4 0.6 0.8
slice1.2
CD8+ T cells
0.0 0.2 0.4 0.6 0.8
slice1.3
CD8+ T cells
0.0 0.2 0.4 0.6 0.8
slice1.4
CD8+ T cells
0.0 0.2 0.4 0.6 0.8
slice1.5
CD8+ T cells
0.00 0.25 0.50 0.75

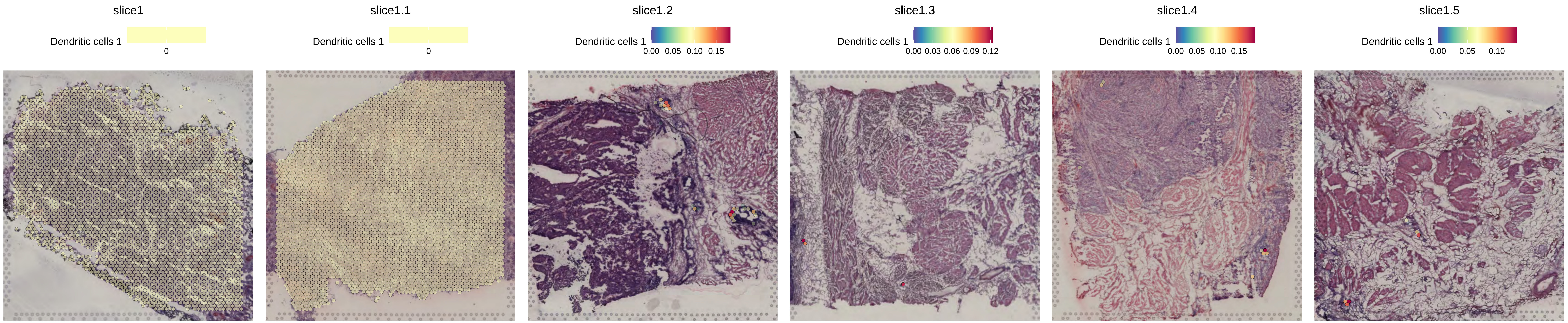
slice1
Dendritic cells 1
0
slice1.1
Dendritic cells 1
0
slice1.2
Dendritic cells 1
0.00 0.05 0.10 0.15
slice1.3
Dendritic cells 1
0.00 0.03 0.06 0.09 0.12
slice1.4
Dendritic cells 1
0.00 0.05 0.10 0.15
slice1.5
Dendritic cells 1
0.00 0.05 0.10

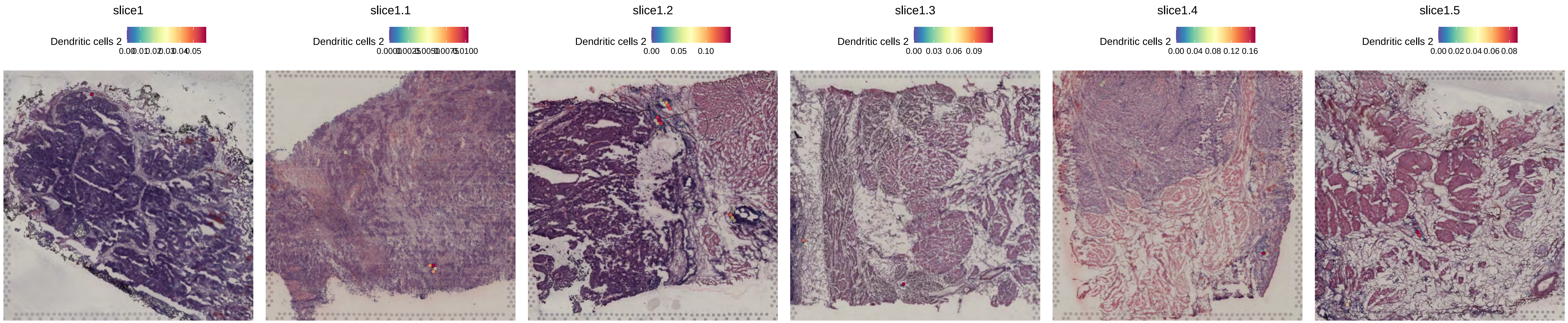
slice1
Dendritic cells 2
slice1.1
Dendritic cells 2
slice1.2
Dendritic cells 2
0.00 0.05 0.10
slice1.3
Dendritic cells 2
0.00 0.03 0.06 0.09
slice1.4
Dendritic cells 2
0.00 0.04 0.08 0.12 0.16
slice1.5
Dendritic cells 2
0.00 0.02 0.04 0.06 0.08

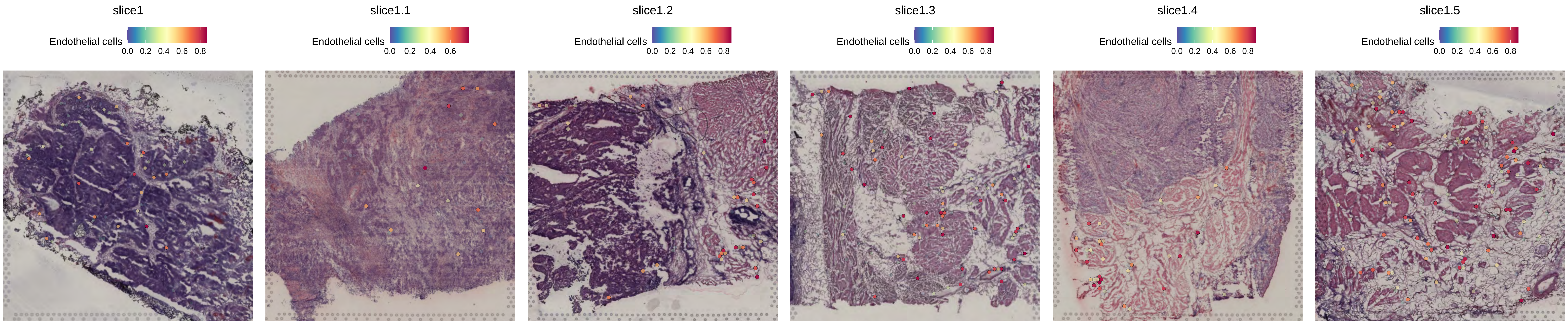
slice1
Endothelial cells
0.0 0.2 0.4 0.6 0.8
slice1.1
Endothelial cells
0.0 0.2 0.4 0.6
slice1.2
Endothelial cells
0.0 0.2 0.4 0.6 0.8
slice1.3
Endothelial cells
0.0 0.2 0.4 0.6 0.8
slice1.4
Endothelial cells
0.0 0.2 0.4 0.6 0.8
slice1.5
Endothelial cells
0.0 0.2 0.4 0.6 0.8

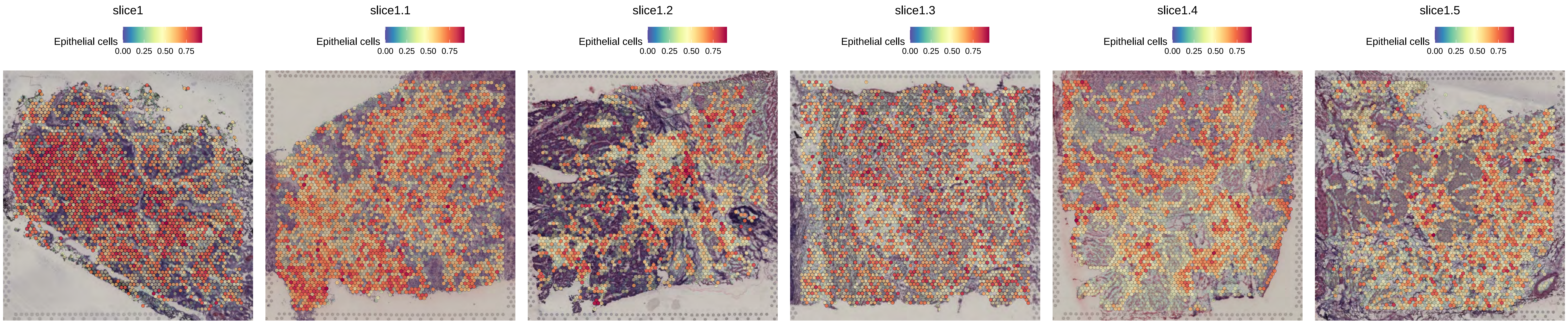

slice1
Epithelial cells
0.00 0.25 0.50 0.75
slice1.1
Epithelial cells
0.00 0.25 0.50 0.75
slice1.2
Epithelial cells
0.00 0.25 0.50 0.75
slice1.3
Epithelial cells
0.00 0.25 0.50 0.75
slice1.4
Epithelial cells
0.00 0.25 0.50 0.75
slice1.5
Epithelial cells
0.00 0.25 0.50 0.75

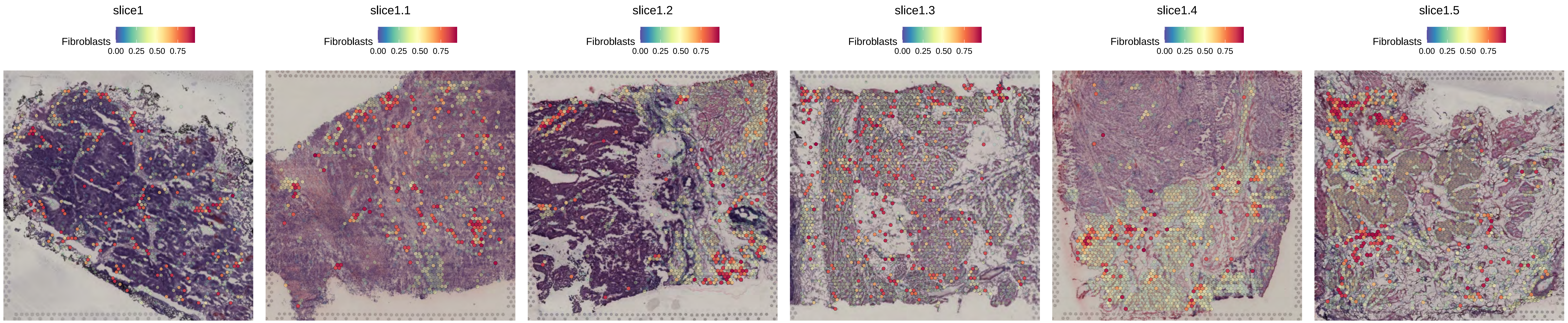
slice1
Fibroblasts
0.00 0.25 0.50 0.75
slice1.1
Fibroblasts
0.00 0.25 0.50 0.75
slice1.2
Fibroblasts
0.00 0.25 0.50 0.75
slice1.3
Fibroblasts
0.00 0.25 0.50 0.75
slice1.4
Fibroblasts
0.00 0.25 0.50 0.75
slice1.5
Fibroblasts
0.00 0.25 0.50 0.75

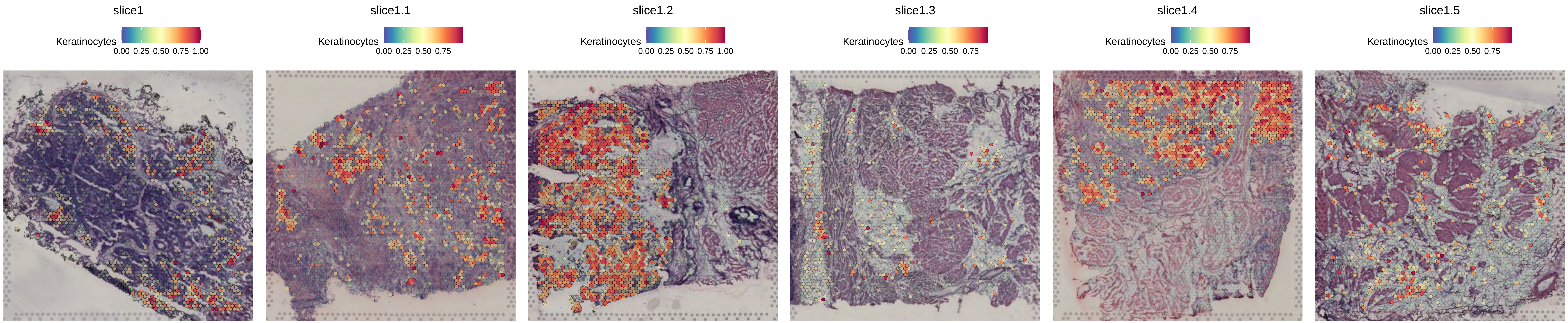
slice1
Keratinocytes
0.00 0.25 0.50 0.75 1.00
slice1.1
Keratinocytes
0.00 0.25 0.50 0.75
slice1.2
Keratinocytes
0.00 0.25 0.50 0.75 1.00
slice1.3
Keratinocytes
0.00 0.25 0.50 0.75
slice1.4
Keratinocytes
0.00 0.25 0.50 0.75
slice1.5
Keratinocytes
0.00 0.25 0.50 0.75

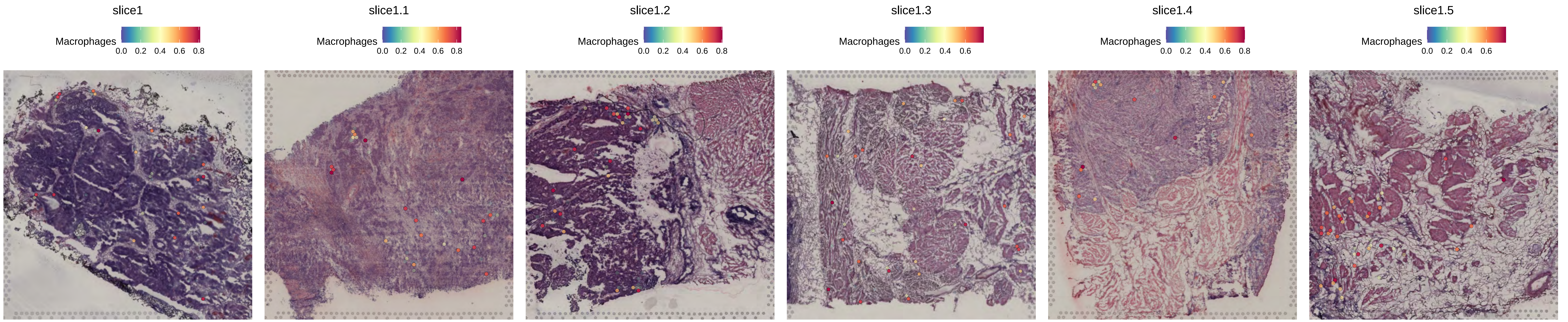

slice1
Macrophages
0.0 0.2 0.4 0.6 0.8
slice1.1
Macrophages
0.0 0.2 0.4 0.6 0.8
slice1.2
Macrophages
0.0 0.2 0.4 0.6 0.8
slice1.3
Macrophages
0.0 0.2 0.4 0.6
slice1.4
Macrophages
0.0 0.2 0.4 0.6 0.8
slice1.5
Macrophages
0.0 0.2 0.4 0.6

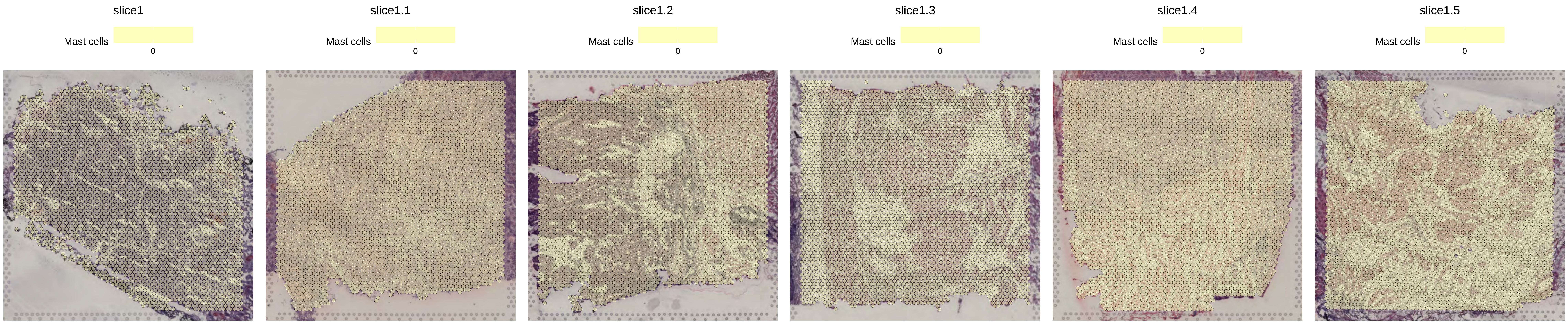
slice1
Mast cells
0
slice1.1
Mast cells
0
slice1.2
Mast cells
0
slice1.3
Mast cells
0
slice1.4
Mast cells
0
slice1.5
Mast cells
0

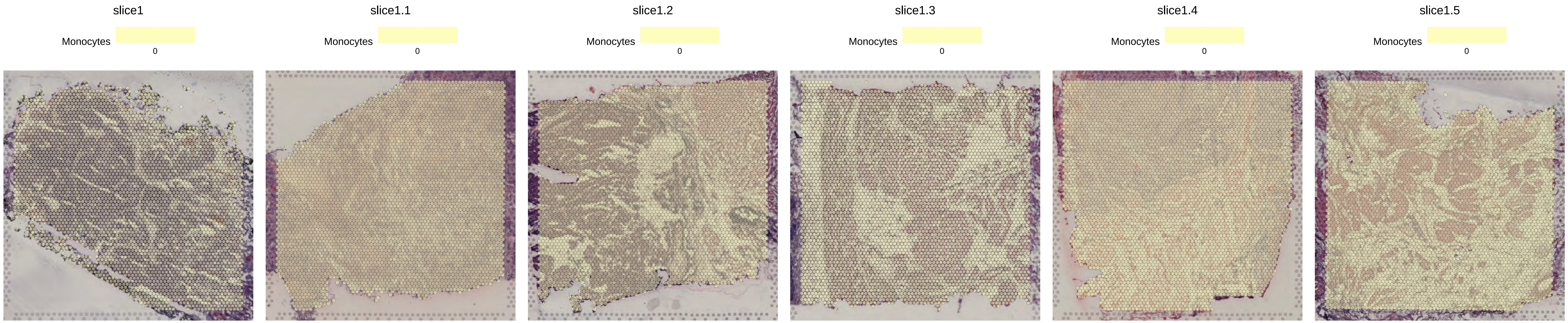

slice1
Monocytes
0
slice1.1
Monocytes
0
slice1.2
Monocytes
0
slice1.3
Monocytes
0
slice1.4
Monocytes
0
slice1.5
Monocytes
0

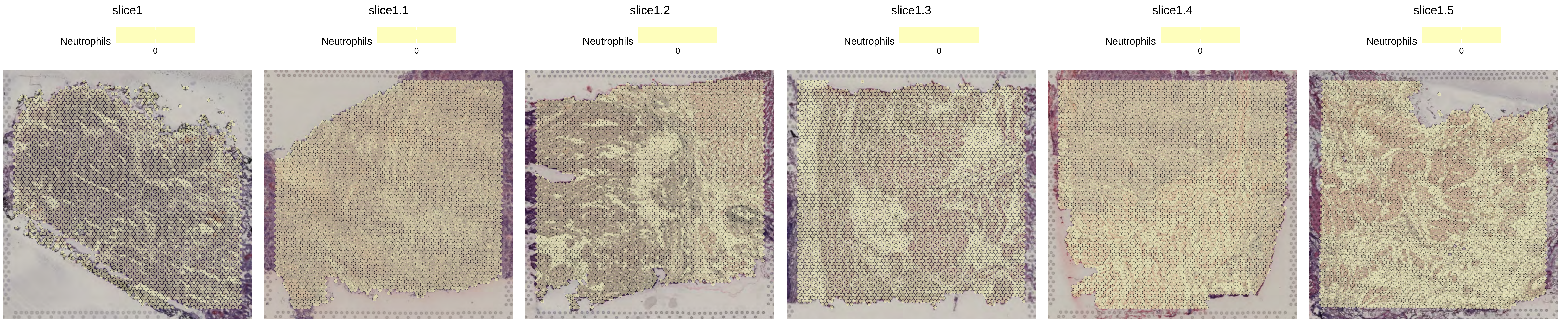

slice1
Neutrophils
0
slice1.1
Neutrophils
0
slice1.2
Neutrophils
0
slice1.3
Neutrophils
0
slice1.4
Neutrophils
0
slice1.5
Neutrophils
0

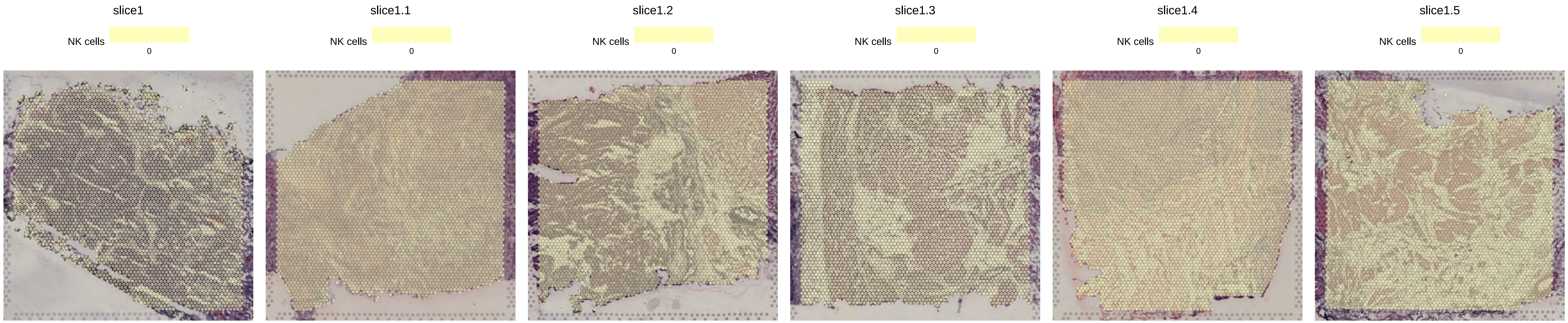
slice1
NK cells
0
slice1.1
NK cells
0
slice1.2
NK cells
0
slice1.3
NK cells
0
slice1.4
NK cells
0
slice1.5
NK cells
0

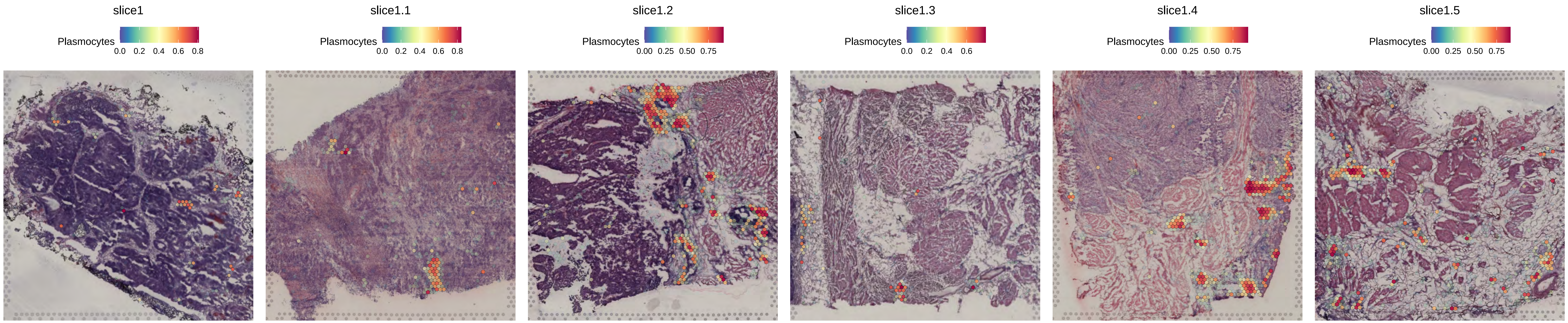
slice1
Plasmocytes
0.0 0.2 0.4 0.6 0.8
slice1.1
Plasmocytes
0.0 0.2 0.4 0.6 0.8
slice1.2
Plasmocytes
0.00 0.25 0.50 0.75
slice1.3
Plasmocytes
0.0 0.2 0.4 0.6
slice1.4
Plasmocytes
0.00 0.25 0.50 0.75
slice1.5
Plasmocytes
0.00 0.25 0.50 0.75

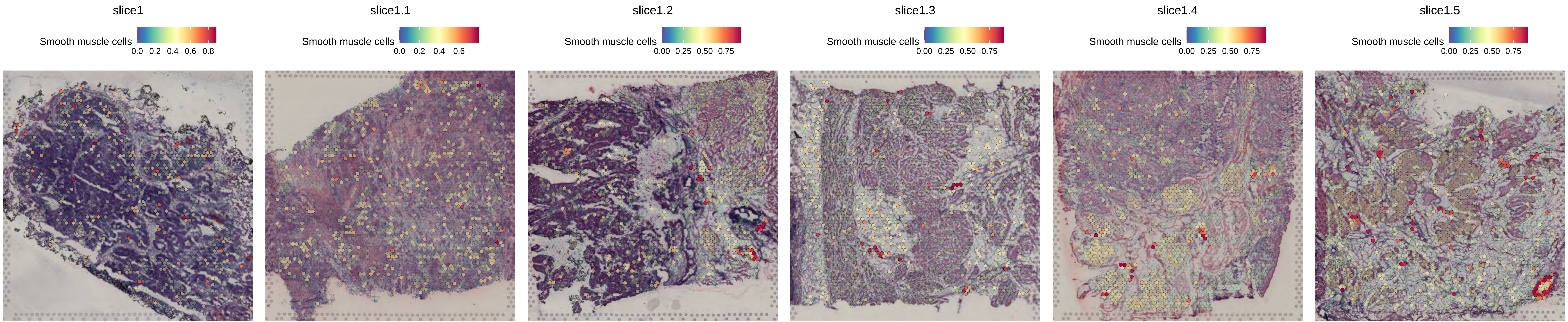
slice1
Smooth muscle cells
0.0 0.2 0.4 0.6 0.8
slice1.1
Smooth muscle cells
0.0 0.2 0.4 0.6
slice1.2
Smooth muscle cells
0.00 0.25 0.50 0.75
slice1.3
Smooth muscle cells
0.00 0.25 0.50 0.75
slice1.4
Smooth muscle cells
0.00 0.25 0.50 0.75
slice1.5
Smooth muscle cells
0.00 0.25 0.50 0.75
